# Benefit from dose-dense adjuvant chemotherapy for breast cancer: subgroup analyses from the randomised phase 3 PANTHER trial

**DOI:** 10.1016/j.lanepe.2024.101162

**Published:** 2024-12-03

**Authors:** Alexios Matikas, Andri Papakonstantinou, Sibylle Loibl, Günther G. Steger, Michael Untch, Hemming Johansson, Nikos Tsiknakis, Mats Hellström, Richard Greil, Volker Möbus, Michael Gnant, Jonas Bergh, Theodoros Foukakis

**Affiliations:** a Karolinska Institutet, Oncology/Pathology Department, Stockholm, Sweden; b Breast Center, Karolinska Comprehensive Cancer Center, Stockholm, Sweden; c German Breast Group, Neu-Isenburg, Germany; d Department of Internal Medicine I, Medical University of Vienna, Vienna, Austria; e Helios Klinikum Berlin-Buch, Berlin, Germany; f 3rd Medical Department, Paracelsus Medical University, Salzburg Austria, Salzburg Cancer Research Institute and AGMT, Salzburg, Austria; g Department of Medicine II, Hematology & Oncology University of Frankfurt, Frankfurt, Germany; h Comprehensive Cancer Center, Medical University of Vienna, Vienna, Austria

**Keywords:** Adjuvant chemotherapy, Amenorrhea, Breast cancer, Dose-dense, PREDICT, STEPP

## Abstract

**Background:**

It is unclear whether some patients with high-risk breast cancer do not warrant adjuvant dose-dense chemotherapy due to small expected absolute benefit.

**Methods:**

The phase 3 PANTHER trial (NCT00798070) compared adjuvant sequential epirubicin/cyclophosphamide (EC) and docetaxel (D) administered in either tailored dose-dense (tDD EC/D) or standard interval schedule (FEC/D) to patients with high-risk resected early breast cancer (n = 2003). We compared outcomes across key subgroups of interest, evaluated the performance of the online prognostication and treatment benefit estimation tool PREDICT and conducted a subpopulation treatment effect pattern plot (STEPP) analysis. Primary endpoint was breast cancer recurrence free survival (BCRFS).

**Findings:**

Median follow-up was 10.3 years. Treatment with tDD EC/D improved 10-year BCRFS across all subgroups including according to menopausal status, with an absolute benefit of 2% or more, as well as in luminal (Hazard Ratio [HR] = 0.83, 95% Confidence Interval [CI] 0.65–1.05) and Human Epidermal Growth Factor Receptor 2 (HER2) positive (HR = 0.53, 95% CI 0.30–0.93), but not triple negative breast cancer patients (HR = 1.02, 95% CI 0.66–1.57). PREDICT underestimated overall survival in the entire population and across all subgroups. In STEPP analysis, absolute benefit from tDD EC/D in BCRFS was stable across risk-defined subpopulations, from 3.8% in the lowest risk patients to 3.6% in the highest risk ones. There was no differential treatment effect over time.

**Interpretation:**

We could not reliably identify any subgroup not benefiting from dose-dense treatment, which should be considered for patients with primary resected high-risk breast cancer.

**Funding:**

10.13039/501100002794Cancerfonden, Bröstcancerförbundet, 10.13039/501100007232Radiumhemmets Forskningsfonder, 10.13039/100002429Amgen, 10.13039/100004337Roche, 10.13039/100004339sanofi-aventis.

**Research in context**

**Evidence before this study**

The results of prior clinical trials investigating the added benefit from dose-dense compared to standard interval adjuvant chemotherapy for non-metastatic breast cancer have been summarized in an overview from the Early Breast Cancer Trialists’ Collaborative Group (EBCTCG), with no new randomised trials identified through a search of the Medline and Embase databases performed in September 2024 using the following search strategy: (((((breast cancer [MeSH Terms]) OR breast neoplasm [MeSH Terms]) OR (“breast cancer” OR “breast tumor” OR “breast tumour” OR “breast adenocarcinoma” OR “mammary adenocarcinoma” OR “breast malignancy” OR “breast malignancies” OR “mammary cancer”)) AND adjuvant chemotherapy [MeSH Terms]) OR (“adjuvant chemotherapy” OR “postoperative chemotherapy” OR “postoperative adjuvant” OR “postoperative” OR “postresection” OR “postresection chemotherapy”)) AND (“dose dense” OR “dose-dense” OR “biweekly” OR “weekly” OR “every two weeks” OR “every 2 weeks” OR “bi-weekly” OR “accelerated”). The EBCTCG meta-analysis showed that the proportional risk reduction by dose-dense regimens is similar regardless of clinical or pathologic factors. However, most trials used control treatment such as paclitaxel every three weeks that was later shown to be inferior to other options, adjuvant trastuzumab was not used in most trials, and endocrine treatment was less optimized than the contemporary standard. These observations have questioned the applicability of the results of the EBCTCG overview to modern practice.

**Added value of this study**

PANTHER is a European phase 3 trial that included patients with high-risk non-metastatic breast cancer and randomised to either dose-dense adjuvant chemotherapy, or optimal standard interval treatment with adequately dosed sequential epirubicin and docetaxel, while all patients received trastuzumab and optimal endocrine treatment as indicated. With long-term follow-up, PANTHER was the first trial to show the superiority of dose-dense regimens regardless of comparator. With the present study, we attempted to identify subgroups that don't require dose-dense treatment. Subgroup analyses according to prognostic factors, breast cancer subtypes, menopausal status (the latter two not previously reported by EBCTCG), the use of PREDICT and exploratory subpopulation treatment effect pattern plot (STEPP) analysis failed to identify any patient group not benefiting from dose-dense chemotherapy.

**Implications of all the available evidence**

Based on end-of-study results of PANTHER and on this analysis and considering that no other trials that examine this issue are ongoing, dose-dense adjuvant chemotherapy should be considered for patients with primary resected, high-risk breast cancer and no medical contraindications.

## Introduction

Compressing time intervals between adjuvant chemotherapy cycles as suggested by the Norton—Simon hypothesis improves patient outcomes compared with standard interval chemotherapy in early breast cancer.^1^^,^^2^ The proportional risk reduction is the same across subgroups defined by tumour size, oestrogen receptor (ER) expression, human epidermal growth factor receptor 2 (HER2) expression, histological type, grade, Ki67, age and nodal status according to a meta-analysis of individual patient data conducted by the Early Breast Cancer Trialists’ Collaborative Group (EBCTCG), although most patients in these analyses were node-positive.^1^ However, the absolute benefit a patient derives from treatment is a function of both the relative risk reduction, in this case similar regardless of any prognostic factors, and the absolute risk of recurrence the patient has. It is thus conceivable that patients at low risk of recurrence do not derive a substantial enough absolute benefit from dose-dense chemotherapy and could potentially represent candidates for standard interval treatment.

The lack of predictive clinical and pathological factors makes it difficult to tailor dose-dense therapy for specific patient groups, a fact that is reflected by contemporary guidelines that either designate both dose-dense and standard interval regimens as preferred options,^3^ or don't give any specific recommendations.^4^ Thus, current clinical practice is largely based on estimation of absolute risk of recurrence and death due to breast cancer in order to identify patients as candidates for omission of chemotherapy or escalation to a dose-dense regimen. In the former case, decision-making is mainly supported by prognostic gene expression profiles that select patients at low risk of recurrence and low to no benefit from adjuvant chemotherapy.^4^ In the latter case however, no such signatures have been validated and estimation of prognosis is commonly based on standard clinical and pathological variables, with or without the help of tools such as PREDICT (www.breast.predict.nhs.uk), which have not been specifically validated for patients treated with dose-dense chemotherapy. In addition, three major randomised trials investigating the use of gene expression profiles for patient selection for adjuvant chemotherapy have shown an interaction between menopausal status and treatment benefit.^5^, ^6^, ^7^ Benefit from chemotherapy in these luminal, lower risk patient populations was restricted to premenopausal women. This observation has sparked a discussion whether chemotherapy effect in premenopausal women mainly depends on ovarian suppression and chemotherapy-induced amenorrhoea (CIA). However, it is unclear whether a similar interplay between menopausal status and chemotherapy effect is noted at the highest end of the risk spectrum.

It is thus clear that patient selection for dose-dense adjuvant chemotherapy is an imprecise art. We have previously reported the primary efficacy analysis and the 10-year update of the Pan-European Tailored Chemotherapy (PANTHER) trial,^8^^,^^9^ an international phase 3 trial that compared tailored dose-dense to standard adjuvant chemotherapy for patients with non-metastatic high risk breast cancer, as described below. Conceivably, in the risk continuum as it is shaped by overlapping prognostic factors and predicted by PREDICT, there might be patients that do not derive sufficient benefit to warrant dose-dense treatment. With the present study, we present long-term survival in key subgroups of interest, investigate the effect of dose-dense treatment based on patient risk and over time, as well as evaluate the performance of PREDICT for this patient population.

## Methods

### Study design

PANTHER (ClinicalTrials.gov identifier NCT00798070) is a prospective, randomised, open-label, multicentre phase III trial, which was conducted in 86 centers in Sweden, Germany and Austria. The trial protocol was approved by ethics review boards at the participating sites and health authorities in all countries. All patients provided written informed consent before inclusion. The study was conducted according to the Declaration of Helsinki and the principles of good clinical practice.

### Study participants

Details regarding the trial design and study population have been previously presented.^8^^,^^9^ Women aged 18–65 years were eligible for the study if they had undergone primary surgery for non-metastatic breast cancer. Eligible patients had either histologically confirmed regional lymph node metastasis, or had node-negative, hormone receptor negative breast cancer at least 20 mm in size and grade 3, or were younger than 35 years regardless of receptor status. Other key inclusion criteria included radical surgery (lumpectomy or mastectomy) with tumour-free margins, removal of at least 5 lymph nodes (or negative sentinel node biopsy for node-negative patients), less than 60 days from surgery to study enrolment, Eastern Cooperative Oncology Group (ECOG) Performance Status 0 or 1, and no major cardiovascular morbidity or other serious medical conditions precluding the safe administration of chemotherapy. All patients provided written informed consent before inclusion.

### Randomisation and masking

Investigators identified and enrolled patients into the trial. Patients were randomly assigned (1:1) into the standard or the experimental treatment groups described hereunder. Randomisation was conducted at one central office per country. Random assignment was stratified by country, participating site and oestrogen receptor status. Random permuted blocks of different sizes (block size of 2, 4 or 6) were used to allocate the patients to each treatment group. PANTHER had an open label design, thus both patients and investigators were aware of treatment allocation.

### Administered treatment and outcomes

Patients allocated to the experimental group received intravenously epirubicin and cyclophosphamide every two weeks for four cycles, followed by 4 cycles of docetaxel every two weeks (tDD EC/D). Dose tailoring for each subsequent cycle in the experimental group was decided according to a predefined algorithm based on leukocyte and platelet nadirs after the previous cycle, but also specific non-hematologic toxicities as previously described in detail.^8^ Patients allocated to standard treatment received intravenously three cycles of 5-fluorouracil and EC every three weeks, followed by three cycles of docetaxel every three weeks (FEC/D). Patients with HER2-positive breast cancer were treated with adjuvant trastuzumab for one year. Adjuvant radiotherapy was administered according to national and local guidelines. Patients with hormone receptor positive breast cancer were treated with endocrine therapy (aromatase inhibitors or tamoxifen) for at least five years, whereas ovarian function suppression (OFS) was recommended for patients who continued to menstruate following the completion of chemotherapy.

The primary end point is breast cancer recurrence free survival (BCRFS), defined as time from randomisation to local, regional, or distant breast cancer relapse, or to death due to breast cancer, whichever occurred first. Secondary efficacy endpoints include distant disease-free survival (DDFS), defined as time from randomisation to distant metastasis or death due to breast cancer; event-free survival (EFS), defined as time from randomisation to breast cancer relapse, contralateral breast cancer, other cancers, or death from any cause; and overall survival (OS), defined as time from randomisation to death from any cause.

### Statistical analysis

The overall goal of this analysis of the PANTHER trial was to study the benefit of dose-dense compared with standard interval adjuvant chemotherapy.

The first aim was to investigate the efficacy of dose-dense compared with standard interval adjuvant chemotherapy across subgroups defined by clinical and pathological factors, as well as in the three immunohistochemistry-defined subtypes, as assessed at each site: hormone receptor positive and HER2-negative (luminal), HER2-positive, or hormone receptor negative and HER2-negative (triple negative). In addition, we investigated the interaction between treatment and menopausal status, both in the intention-to-treat population and per breast cancer subtype, and the associated risk of gonadal toxicity, defined in this study as the cessation of menstruation following treatment and up until two years after treatment completion in patients who were premenopausal at baseline, without receiving OFS.

We then evaluated the effect of tDD EC/D across patient risk groups. Subpopulation Treatment Effect Pattern Plot (STEPP) analysis is a method that plots treatment effect for overlapping patient groups according to a continuous variable,^10^ which in this case was a clinical composite risk score. This score was calculated as previously described,^11^ using a Cox regression model including six clinical and pathologic variables (age, hormone receptor status, HER2 status, tumour size, nodal status and grade) and adding the LogHR scores for each variable minus the average LogHR score. The net result is the clinical composite risk score, which ranges from −1.12 (lowest risk) to 1.89 (highest risk). For illustrative purposes, the distribution of patient characteristics when dividing the total population into four groups according to the clinical composite risk score is shown in Supplementary Table S1, whereas patient outcomes per group are shown in Supplementary Figure S1. We then performed a sliding window version of STEPP,^12^ as previously described and employed in adjuvant breast cancer trials,^11^^,^^13^ for the primary endpoint of BCRFS. The overall population was divided into seven subpopulations, consisting of 400 patients per subpopulation (r2 = 400) and up to 200 patients common in two neighboring subpopulations (r1 = 200). Finally, we investigated the effect of tDD EC/D compared with FEC/D over time. Follow-up was divided into four time bands (0–3 years, 3–6 years, 6–9 years, 9+ years). We estimated the effect of allocated treatment on survival outcomes (BCRFS, EFS, DDFS, OS) in each time band using Cox regression and tested for interaction between time band and treatment group. We also plotted the failure rates per treatment group (tDD EC/D versus FEC/D) in annual intervals during the first five years of follow-up and following the fifth year.

The second part of this analysis concerned the validation of the PREDICT online tool for patients treated with adjuvant dose-dense chemotherapy. PREDICT is a well-validated online tool used to aid decision making in the adjuvant setting.^14^, ^15^, ^16^ Its accuracy, however, for calculating chemotherapy benefit when patients are exposed to dose-dense treatment is unclear. The following programs, libraries and packages were used to calculate PREDICT version 2.1 calculation: R version 4.2.2, nhs.predict version 1.4.0, dplyr version 1.1.2. We assessed the agreement between predicted overall and observed survival rates (calibration) at 5 and 10 years. In addition, we investigated the ability of PREDICT to identify patients with an event (death) or not at 5 and 10 years (discrimination), using area under the receiver operator characteristic curve (AUC under ROC) for 5-year and 10-year predicted OS. Subgroup analyses for calibration and discrimination were performed in subgroups of interest according to tumour size, nodal status, hormone receptor and HER2 expression.

Fisher's exact test was used to compare categorical variables and t-test for continuous ones. Time for event-free patients in all survival analyses was calculated from the date of randomisation to the date of last clinical visit. Survival was estimated with the Kaplan–Meier method. The graphs represent time to failure. Proportional hazards regression models were used to estimate the effect of treatment, menopausal status and the interaction between treatment and menopausal status on time to failure. Results from the models are presented as hazard ratios (HR) together with 95% confidence intervals (CI). p values from these models refer to Wald tests and values of less than 0.05 (two-sided) were considered to be statistically significant. All analyses were performed using the intention-to-treat principle.

### Role of the funding source

The funding sources had no access or input to any of the following: study design; the collection, analysis, and interpretation of data; the writing of the report; and the decision to submit the paper for publication.

## Results

### Patient characteristics and administered therapies

Between February 2007 and September 2011, 2017 patients were enrolled in the trial. Of those, 11 withdrew consent shortly after randomisation and 3 were lost to follow-up at day 0. As such, 2003 patients comprise the intention-to-treat population (ITT). The patients’ clinical and demographic characteristics according to allocated treatment are presented in Supplementary Table S2 while the Consolidated Standards of Reporting Trails (CONSORT) diagram is shown in Supplementary Figure S2. Median follow-up is 10.3 years (interquartile range, 9.1–10.5 years).

Details on administered chemotherapy cycles, median cumulative doses and reasons for premature treatment discontinuation have been previously described.^8^^,^^17^^,^^18^ Adjuvant trastuzumab was administered to 97.9% (335 of 342) of all HER2-positive breast cancer patients, with most (91.3%, 306 of 335) completing one year of treatment. Reasons for non-administration were withdrawal from study (n = 3), lost to follow-up (n = 2) and other reasons (n = 2). Trastuzumab was started after the last docetaxel cycle in 59% (202 of 342) of patients (after a median of 32 days) and concomitantly in 41% (140 of 342) of patients.

In total, 1600 patients with hormone receptor positive breast cancer were enrolled, of which 1579 had ER-positive tumours and 1561 had available information on administered endocrine therapy. During the first five postoperative years, 547 of 1561 patients (35%) received tamoxifen, with or without OFS, 570 of 1561 patients received aromatase inhibitors with or without OFS (36.5%), 435 of 1561 patients received both tamoxifen and aromatase inhibitors (27.8%), whereas 9 of 1561 patients were treated with OFS alone (0.5%). In addition, 813 ER-positive patients were premenopausal at enrolment, prior to chemotherapy start. OFS was routinely recommended to patients who remained premenopausal following chemotherapy and was administered during the first five postoperative years to 202 of 813 patients (24.8% of all patients premenopausal at diagnosis). At the time of the sixth annual follow-up, 1361 patients (698 in tDD EC/D and 663 in FEC/D group) were alive and distant recurrence free. Of those, 590 of 1361 patients (43.5%) were still under endocrine therapy, 283 in the experimental and 307 in the control group. Choice of endocrine treatment, and use of OFS and extended endocrine treatment were balanced between the two treatment groups.

### Cumulative incidence of breast cancer relapse events in key subgroups of interest

Table 1 presents the cumulative incidence of BCRFS events in the two treatment groups and the corresponding difference between them. The experimental treatment consistently improved ten-year BCRFS with no evidence of differential benefit depending on any clinicopathologic factor. The largest observed difference was according to HER2 status, where HER2-positive breast cancer patients experienced a numerically greater absolute benefit from tDD EC/D in terms of cumulative BCRFS events compared to HER2-negative ones (10.9% versus 2.2% respectively, p_interaction_= 0.093). The absolute difference between the two treatments was around or higher than 2% in all subgroups of interest. Supplementary Table S3 presents the cumulative incidence of deaths in the two treatment groups and the corresponding difference between them. As previously reported,^9^ the difference in OS in the intention-to-treat population was not statistically significant. Subgroups benefiting the most from tDD EC/D (4% or more absolute OS gain) were patients with ER-negative tumours, with HER2-positive tumours and with grade 3 tumours.

**Table 1:** Cumulative incidence % of breast cancer recurrence free survival events at 10 years with corresponding 95% confidence intervals, per treatment arm.

Factor	Group	FEC/D	tDD EC/D	Difference	p_interaction_
All patients		22.3 (19.7–25.1)	18.6 (16.1–21.3)	−3.7 (−7.4 to 0.1)	
Age, years	<50	21.9 (18.2–25.8)	20.1 (16.3–24.2)	−1.8 (−7.2 to 3.7)	0.32
	≥50	22.8 (19.1–26.7)	17.4 (14.2–21.0)	−5.3 (−10.5 to −0.2)	
Positive nodes, number	0–3	14.3 (11.5–17.4)	12.4 (9.8–15.4)	−1.9 (−5.9 to 2.2)	0.84
	≥4	33.5 (28.8–38.3)	29.0 (24.1–33.9)	−4.6 (−11.4 to 2.3)	
Tumour size, mm	≤20	14.8 (11.4–18.6)	11.7 (8.6–15.2)	−3.1 (−8.0 to 1.8)	0.67
	>20	27.5 (23.8–31.3)	23.8 (20.2–27.6)	−3.7 (−9.0 to 1.7)	
Hormone receptor status	Negative	28.8 (22.7–35.1)	26.3 (20.2–32.9)	−2.4 (−11.4 to 6.5)	0.53
	Positive	20.6 (17.7–23.6)	16.7 (14.0–19.6)	−3.9 (−8.0 to 0.2)	
HER2 status	Negative	22.4 (19.5–25.5)	20.2 (17.4–23.2)	−2.2 (−6.4 to 2.0)	0.093
	Positive	21.8 (15.9–28.3)	10.9 (6.6–16.4)	−10.9 (−18.8 to −3.0)	
Tumour grade	1–2	19.3 (16.0–22.9)	16.5 (13.2–20.1)	−2.8 (−7.7 to 2.1)	0.96
	3	26.0 (21.9–30.4)	21.7 (17.9–25.7)	−4.3 (−10.1 to 1.4)	
Ki-67 positive cells, %	≤20	19.1 (14.6–24.1)	16.2 (11.9–21.1)	−2.9 (−9.5 to 3.7)	0.92
	>20	25.2 (20.6–30.0)	21.3 (16.9–26.0)	−3.9 (−10.4 to 2.7)	

Abbreviations: FEC, 5-fluorouracil; epirubicin, cyclophosphamide; D, docetaxel; tDD, tailored and dose-dense; EC, epirubicin, cyclophosphamide.

For the relative benefit in each subgroup the reader is referred to reference.^9^

**Fig. 1:**
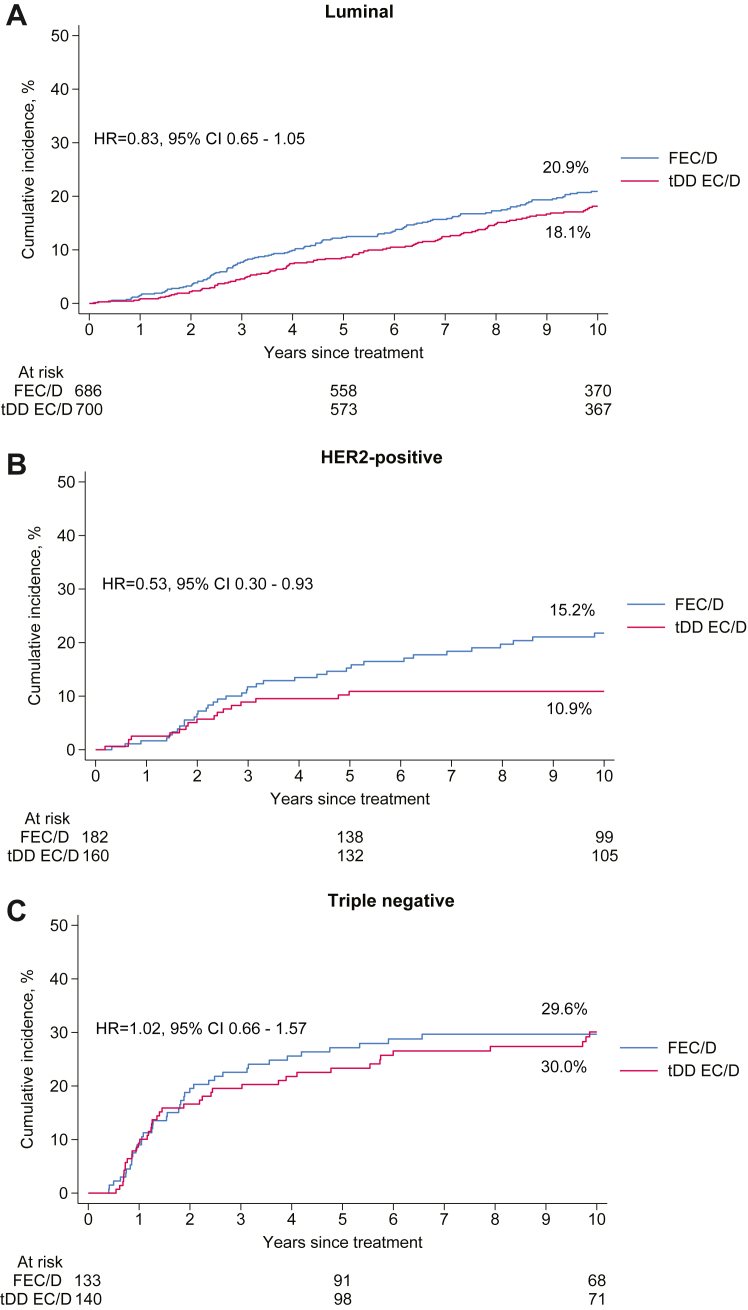
Cumulative incidence curves of breast cancer recurrence free survival events per treatment group and corresponding ten-year event rates in patients with hormone receptor positive and HER2-negative (A), HER2-positive (B) and triple negative breast cancer (C). p value for interaction is 0.182. Abbreviations: FEC, 5-fluorouracil, epirubicin, cyclophosphamide; D, docetaxel; tDD, tailored dose-dense; EC, epirubicin, cyclophosphamide; HR, hazard ratio; CI, confidence interval.

Among patients with luminal and patients with HER2-positive breast cancer, tDD EC/D improved BCRFS compared to FEC/D (HR = 0.83, 95% CI 0.65–1.05, p = 0.12 and HR = 0.53, 95% CI 0.30–0.93, p = 0.026, respectively). Within the HER2-positive subgroup, tDD EC/D improved BCRFS regardless of HR status (positive: HR = 0.49, 95% CI 0.22–1.08; negative: HR = 0.62, 95% CI 0.28–1.37; p_interaction_ = 0.71). There was no difference between the two treatment groups among patients with triple negative disease (HR = 1.02, 95% CI 0.66–1.57, p = 0.93), though there was no interaction between breast cancer subtype and treatment (p_interaction_ = 0.18). The corresponding cumulative incidence curves of BCRFS events per subtype are shown in Fig. 1. No interaction was observed between subtype and treatment for the OS endpoint either (p_interaction_ = 0.33), with HR point estimates favoring tDD EC/D in luminal (HR = 0.86, 95% CI 0.64–1.16), HER2-positive (HR = 0.54, 95% CI 0.30–0.99), but less so in triple negative breast cancer patients (HR = 0.90, 95% CI 0.57–1.41).

### Interaction between treatment and menopausal status

Data on baseline menopausal status were available from 1913 patients, which is the study population of this analysis (Supplementary Figure S3). The patient characteristics of the 1036 premenopausal and 877 postmenopausal patients are presented in Supplementary Table S4. Tumour size, nodal burden, hormone receptor status, HER2 status and allocation to treatment were balanced between the two groups.

In the intention-to-treat population there was no interaction between menopausal status and treatment (p_interaction_ = 0.64), with the proportional risk reduction from tDD EC/D compared to FEC/D being similar in premenopausal (HR = 0.85, 0.64–1.12, p = 0.23) and postmenopausal patients (HR = 0.77, 95% CI 0.57–1.03, p = 0.076). Similar results were noted when focusing on HR-positive patients only (premenopausal: HR = 0.71, 95% CI 0.51–1.00, p = 0.052; postmenopausal: HR = 0.81, 95% CI 0.58–1.13, p = 0.22; p_interaction_ = 0.58; Supplementary Figure S4).

At the two-year timepoint following the completion of adjuvant chemotherapy, 128 premenopausal at baseline and hormone receptor positive patients who were treated with tdd EC/D and 127 treated with standard interval treatment did not menstruate, without receiving OFS. The odds ratio [OR] for CIA was OR = 1.04 (95% CI 0.77–1.39).

There were 592 patients who were premenopausal at study inclusion without menstruation at two years regardless of reason and 57 that continued to menstruate. The mean age was 45.7 versus 40.9 years (p < 0.001). Numerically fewer BC relapse events were observed among patients with no menstruation at two years in both treatment arms compared with those who continued to menstruate (14.7% versus 19.3%), although the difference was not statistically significant (p = 0.35).

### Effect of dose-dense adjuvant chemotherapy across risk groups

**Fig. 2:**
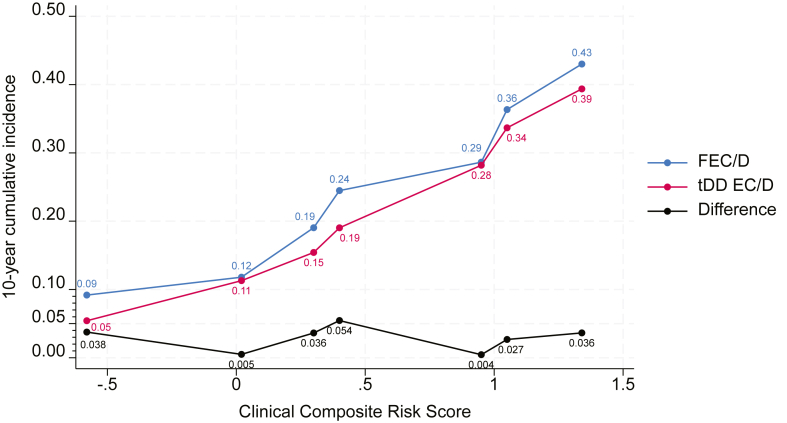
Subpopulation treatment effect pattern plot (STEPP). The y axis depicts cumulative incidence of events for the primary endpoint of breast cancer recurrence free survival. The x axis depicts continuous clinical composite risk score, from lowest to highest. Abbreviations: FEC, 5-fluorouracil, epirubicin, cyclophosphamide; D, docetaxel; tdd, tailored dose-dense; EC, epirubicin, cyclophosphamide.

The clinical composite risk score as a continuous variable was prognostic for BCRFS (HR = 2.63, 95% CI 2.13–3.25, p < 0.001) and there was no interaction with treatment (p = 0.67). The results of the STEPP analysis according to composite risk score are shown in Fig. 2. The plot depicts the cumulative 10-year incidence of BCRFS events in each of the seven overlapping subpopulations defined for the STEPP analysis per treatment group (tDD EC/D and FEC/D), as well as the effect of dose-dense therapy, expressed as the difference between the two treatments in 10-year BCRFS percentages for each subpopulation. There was no evidence of variability in absolute benefit from dose-dense treatment depending on clinical composite risk score, with the difference in 10-year BCRFS rates in favour of tDD EC/D ranging from 3.8% in the lowest risk patients to 3.6% in the highest risk ones. Moreover, no interaction based on cumulative incidence estimates was observed between treatment and overlapping subpopulations (p = 0.90). Sensitivity analysis by removing prognostic factors that were also predictive for background therapies, but not for dose-dense chemotherapy as shown in Table 1 (ER, HER2), yielded unchanged results, with a numerically higher benefit from dose-dense chemotherapy for the highest risk patients, though no continuously increasing benefit with increasing risk and no interaction with treatment arm, as described in Supplementary Figure S5.

### Effect of dose-dense adjuvant chemotherapy over time

Supplementary Table S5 presents hazard ratios for the comparison of tdd EC/D with FEC/D (reference) for each of the four survival endpoints in each of the four time periods, together with interaction tests between treatment and time period. In summary, there was no evidence of differential effect of dose-dense compared to standard interval adjuvant chemotherapy over time. The point estimates in the period of nine years after study enrolment or later reflect the small number of events, considering that median follow-up in the trial was 10.3 years. Visual inspection of the failure rate plots showed that the peak seen in the control group in terms of breast cancer recurrence and distant recurrence at 2–3 years postoperatively seems to be avoided following dose-dense treatment (Supplementary Figure S6), however this was not mirrored in the OS plot.

### Validation of PREDICT for dose-dense adjuvant chemotherapy

We evaluated the performance of PREDICT in terms of model calibration by comparing the predicted and observed OS rates for the 1961 patients out of 2003 who comprise the ITT with all available data to generate the PREDICT output. Of the missing 42 patients, 33 lacked information on adjuvant endocrine therapy and/or bisphosphonates, 6 on grade, 2 on HR status and 1 on tumour size. Patient characteristics are shown in Supplementary Table S2.

**Fig. 3:**
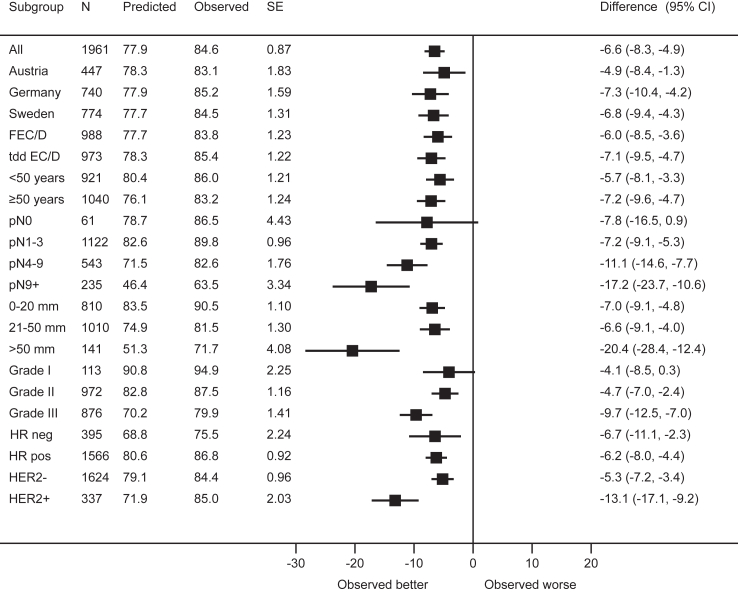
Estimated according to the PREDICT model and observed overall survival rates at 10 years. Abbreviations: SE: standard error; CI: confidence interval; FEC, 5-fluorouracil, epirubicin, cyclophosphamide; D, docetaxel; tdd, tailored dose-dense; EC, epirubicin, cyclophosphamidel; HR, hormone receptor; HER2, human epidermal growth factor receptor 2.

The predicted 10-year OS in the entire cohort was 77.9% compared to the observed 84.6% (Δ = −6.6%, 95% Confidence Interval [CI] −8.3% to −4.9%). Across clinically relevant subgroups, the predicted 10-year OS rates were worse than the observed ones, with underestimation ranging from 4.1% (95% CI 0.3%–8.5%) for grade 1 tumours to 20.4% (95% CI 12.5%–28.4%) for tumours larger than 5 cm. In general, the absolute difference between predicted and observed rates was higher in high-risk subgroups. These results are summarized in Fig. 3. PREDICT underestimated 5-year OS as well, in the entire cohort (90.4% predicted versus 91.8% observed survival) and across all subgroups, although the absolute difference with observed survival was lower (Supplementary Figure S7).

We then assessed model discrimination for 5-year and 10-year OS in the entire cohort and subgroups of interest (Supplementary Table S6). For the 5-year timepoint, AUC under the ROC curve in the entire cohort was 0.786 (95% CI 0.748–0.824) and ranged from 0.705 to 0.843 in relevant subgroups. Discriminatory accuracy was lower for 10-year OS in the entire cohort with an AUC of 0.728 (95% CI 0.696–0.760), and across all subgroups of interest ranging from 0.611 to 0.789.

Finally, we validated the performance of PREDICT following dose-dense chemotherapy in terms of 10-year OS, both in the entire population and in subgroups of interest. PREDICT consistently underperformed for the prediction of outcomes in the experimental group compared to the standard group across all subgroups defined by clinical and pathologic factors (Table 2).

**Table 2:** Discriminatory accuracy of PREDICT model expressed as area under the receiver operator characteristic curve and corresponding 95% confidence interval for 10-year overall survival per allocated treatment.

	FEC/D	tDD EC/D
**Entire population**	0.740 (0.696–0.784)	0.714 (0.668–0.761)
**Country**		
Austria	0.774 (0.702–0.847)	0.772 (0.672–0.871)
Germany	0.728 (0.646–0.811)	0.741 (0.658–0.825)
Sweden	0.738 (0.663–0.811)	0.675 (0.606–0.744)
**Age**		
<50 years	0.757 (0.694–0.820)	0.749 (0.683–0.815)
≥50 years	0.720 (0.657–0.783)	0.688 (0.621–0.755)
**Number of positive nodes**		
0	0.659 (0.464–0.855)	0.586 (0.217–0.954)
1–3	0.662 (0.586–0.737)	0.682 (0.608–0.756)
4–9	0.746 (0.670–0.823)	0.617 (0.526–0.708)
>9	0.708 (0.611–0.806)	0.624 (0.512–0.736)
**Tumour size (cm)**		
≤2	0.760 (0.682–0.839)	0.740 (0.652–0.828)
2–5	0.690 (0.627–0.753)	0.649 (0.584–0.714)
>5	0.798 (0.673–0.922)	0.793 (0.682–0.903)
**Tumour grade**		
1	0.653 (0.521–0.786)	0.543 (0.000–1.000)
2	0.726 (0.655–0.797)	0.714 (0.639–0.789)
3	0.715 (0.653–0.778)	0.681 (0.615–0.747)
**Hormone receptor status**		
ER or PR positive	0.717 (0.661–0.774)	0.704 (0.647–0.761)
ER and PR negative	0.723 (0.640–0.807)	0.685 (0.600–0.770)
**HER2 status**		
Negative	0.747 (0.699–0.795)	0.733 (0.683–0.783)
Positive	0.722 (0.608–0.835)	0.618 (0.476–0.759)

Abbreviations: FEC: 5-fluorouracil, epirubicin, cyclophosphamide; D, docetaxel; tDD, tailored and dose-dense; EC, epirubicin, cyclophosphamide; ER, oestrogen receptor; PR, progesterone receptor; HER2, human epidermal growth factor receptor 2.

## Discussion

We aimed to identify patient groups where the expected absolute benefit would be so low that dose-dense treatment should not be used or, in contrast, to identify patients benefiting particularly from dose-dense treatment. Expectedly, the observed absolute benefit from tDD EC/D was higher in higher risk groups, such as grade 3 tumours, those with multiple positive nodes or high Ki67. Although the definition of sufficient absolute benefit in relation to toxicity is not strictly defined and such a threshold should be individualised according to each patient's values and preferences, the expected additional toxicity with dose-dense treatment is small according to the EBCTCG meta-analysis^1^ and prior results from PANTHER.^9^^,^^17^^,^^19^ Moreover, we could not find evidence supporting an interaction between menopausal status and dose-dense chemotherapy, corroborating previous findings from a pooled analysis of two trials.^20^ In both the pooled analysis and in PANTHER dose-dense treatment was not associated with an increased risk of CIA, although possible confounding factors are the non-randomised allocation to OFS following adjuvant chemotherapy and self-reported menopausal status. These observations, which are in line with the lack of interaction between dose-dense treatment and age,^1^ suggest that the benefit of high-risk patients from dose-dense treatment goes beyond the mere induction of menopause.

Furthermore, the EBCTCG overview reported only on treatment effect according to individual prognostic factors and not per immunohistochemistry-based subtype. While no formal interaction between subtype and treatment was observed, the lack of benefit from tDD EC/D in triple negative patients is notable. This finding is in contrast with results reported by the Gruppo Italiano Mammella 2 (GIM2) trial where the superiority of dose-dense treatment to a control of three-weekly EC and paclitaxel was consistent across subtypes.^21^ Whether our results are due to a chance finding, an inherent disadvantage of dose tailoring for triple negative disease, or true absence of further improvement of outcomes in triple negative patients over standard interval, but otherwise optimal sequential anthracycline and taxane is unclear. In support of the latter are the observed ten-year disease-free survival rates in GIM2 (approximately 70% with dose-dense treatment versus just over 50% with standard treatment) and PANTHER (70% in both groups), difficulties in cross-trial comparisons notwithstanding. These findings imply that a 30% long-term risk for recurrence is expected following treatment with anthracycline and taxane, with further improvements stemming from response-based postneoadjuvant treatment^22^ and addition of carboplatin^23^ and pembrolizumab.^24^ On the other hand, the fact that tDD EC/D improved outcomes of primary resected HER2-positive breast cancer treated with adjuvant trastuzumab, underscores the importance of incorporating dose-dense regimens into the management of patients with no contraindication to anthracyclines.

A disadvantage of evaluating treatment effect in dichotomized groups according to prognostic factors, besides risk for misleading associations which is not mitigated in exploratory STEPP analyses, is the difficulty in translating their results to clinical practice. The constellation of prognostic tumour characteristics overlaps and forms a risk spectrum, making the exact treatment benefit a patient derives unclear. For this reason, STEPP analyses have been previously employed in large adjuvant breast cancer trials to visualise treatment effect in overlapping subpopulations. For example, such an analysis from the Suppression of Ovarian Function Trial (SOFT) and the Tamoxifen and Exemestane Trial (TEXT) trials showed increasing absolute benefit from ovarian function suppression and exemestane with increasing risk for recurrence,^13^ while in APHINITY no subpopulation with lower benefit from pertuzumab could be identified within the node-positive subgroup.^11^ In PANTHER, we could not identify a patient subpopulation that could safely de-escalate to standard interval treatment. Thus, we conclude that dose-dense adjuvant chemotherapy should be considered for all patients with primary resected high-risk breast cancer. Importantly, after long-term follow-up the proportional risk reduction in all four time-to-failure endpoints examined in PANTHER was stable over time with an early decrease in event rate that sustained for a decade following breast surgery.

In PANTHER, a cohort of patients with high-risk breast cancer treated with effective systemic therapies in accordance with modern standards, we observed that PREDICT systematically overestimated 10-year risk for death across all subgroups, more so in very high-risk patients such as those with large tumours or multiple positive nodes. Possible reasons why PREDICT underperformed in our study include weak external validity of randomised controlled trials,^25^ underestimation by PREDICT of survival of patients with HER2-positive breast cancer that fared particularly well in PANTHER,^26^ improved systemic therapy (chemotherapy and endocrine therapy) in PANTHER, overperformance of control arm,^27^ overestimation by PREDICT of competing causes of death in this high-risk cohort and a minority of patients that hadn't completed ten years of follow-up. While there were small differences in outcome prediction between the two treatment groups implying that no specific adjustments for dose-dense therapy should be made to the model, our results corroborate previous findings from clinical trials^26^ and population based studies.^28^ In light of these results, further accentuated by the approval of adjuvant therapies specifically for high-risk populations that are not currently included in PREDICT such as abemaciclib^29^ and pertuzumab,^30^ the clinical validity and utility of PREDICT should be reevaluated.

A limitation of our study is that the treatment approach to early breast cancer has developed significantly since PANTHER was conducted, including the introduction of new agents such as immune checkpoint inhibitors, inhibitors of cyclin dependent kinases 4/6 and olaparib, the optimization of risk stratification, and the emergence of complex neoadjuvant strategies.^4^ Due to these advances, the results of the EBCTCG meta-analysis were met with some scepticism, due to outdated control treatments and the availability of gene expression profiling.^31^ The optimal control treatment in PANTHER instead of the inferior three-weekly paclitaxel regimen previously employed,^21^^,^^32^ universal use of trastuzumab, modern endocrine treatment strategies and use of bisphosphonates, facilitate the translation of our results to contemporary practice, considering also the ubiquitous use of a chemotherapy backbone of anthracyclines and taxanes in most clinical scenarios of high risk breast cancer and the ongoing predominance of primary surgery over neoadjuvant strategies.^33^^,^^34^ Another limitation that should be acknowledged is the open label nature of the study, which may have influenced outcomes considering the subjective nature of some endpoints.

In conclusion, dose-dense treatment improved outcomes compared to standard interval treatment consistently regardless of prognostic factors, breast cancer subtypes, menopausal status or continuous risk, at the cost of more acute toxicity. In addition, PREDICT was inadequately accurate for prognostic estimation for the trial population. Considering the relevant EBCTCG meta-analysis,^1^ but also the lack of OS benefit in PANTHER,^9^ dose-dense treatment should be considered as an option for all patients with primarily resected high-risk breast cancer and no contraindications due to age or comorbidity.

## Contributors

Concept and design: Matikas, Papakonstantinou, Steger, Untch, Gnant, Loibl, Möbus, Bergh, Foukakis. Acquisition, analysis, or interpretation of data: Matikas, Möbus, Steger, Untch, Hellström, Greil, Gnant, Loibl, Bergh, Foukakis. Drafting of the initial manuscript: Matikas, Papakonstantinou, Johansson, Tsiknakis, Bergh, Foukakis. Critical revision of the manuscript for important intellectual content: all authors, Statistical analysis: Matikas, Johansson, Tsiknakis. Obtained funding: Steger, Gnant, Loibl, Bergh. Administrative, technical, or material support: Papakonstantinou, Steger, Untch, Hellström, Greil, Gnant, Loibl, Bergh, Foukakis. Study supervision: Matikas, Möbus, Steger, Untch, Gnant, Loibl, Bergh, Foukakis.

## Data sharing statement

Study data are not publicly available due to lack of permission from the Swedish, German and Austrian national ethical review authorities. Data sharing will be reviewed in a case-by-case basis, approved by the steering committee and ethical permit will be applied for.

## Declaration of interests

Alexios Matikas: speaker/consultancy (no personal fees) to Veracyte, Roche, Seagen; research funding paid to institution by Merck, AstraZeneca, Novartis, Veracyte.

Sibylle Loibl: employment as Chief Executive Officer (CEO) at German Breast Group (GBG) Forschungs GmbH; institutional fees for advisory board membership for AbbVie, Amgen, AstraZeneca, Bristol Myers Squibb (BMS), Celgene, DSI, EirGenix, Gilead, GSK, Lilly, Merck, Novartis, Olema, Pfizer, Pierre Fabre, Relay Therapeutics, Roche, Sanofi and Seagen; institutional fees as an invited speaker for AstraZeneca, DSI, Gilead, Medscape, Novartis, Pfizer, Roche, Seagen and Stemline-Menarini; institutional research grants from AbbVie, AstraZeneca, BMS/Celgene, Daiichi Sankyo, Immunomedics/Gilead, Molecular Health, Stemline-Menarini, Novartis, Pfizer and Roche; institutional funding from Greenwich Life Sciences; institutional licensing fees from VMscope GmbH; a role as a steering committee member (non-financial interest) for AstraZeneca, Daiichi Sankyo, Immunomedics/Gilead, Novartis, Pfizer, Roche and Seagen; a role as a Principal Investigator (PI) for Pfizer, AstraZeneca (non-financial interest).

Günther Steger: personal fees and non-financial support from Roche, personal fees and non-financial support from AstraZeneca, personal fees and non-financial support from Novartis, personal fees from Lilly, non-financial support from TEVA, personal fees and non-financial support from Pfizer.

Michael Untch: personal fees for lectures and/or consultancy from Agendia, AstraZeneca, Daiichi Sankyo, Eisai Gilead, Lilly Deutschland, MSD, Myriad Genetics, Novartis, Pierre Fabre, Pfizer, Roche, Sanofi Aventis, Seagen, Stemline

Richard Greil: institutional grants for research and studies from Celgene, Roche, Merck, Takeda, AstraZeneca, Novartis, Amgen, BMS, MSD, Sandoz, Abbvie, Gilead, and Daiichi Sankyo, and honoraria for lectures or consultancy from Celgene, Roche, Merck, Takeda, AstraZeneca, Novartis, Amgen, BMS, MSD, Sandoz, Abbvie, Gilead, Daiichi Sankyo, and Sanofi.

Michael Gnant: personal fees for advisory board membership for Eli Lilly, MSD, Novartis and Menarini-Stemline; personal fees as an invited speaker for AstraZeneca, Daiichi Sankyo, Eli Lilly, EPQ Health, Novartis and Pierre Fabre; personal fees for an expert testimony for Veracyte; membership of the Board of Directors at Austrian Breast and Colorectal Cancer Study Group (ABCSG) GmbH and ABCSG Research Services GmbH; a role as a steering committee member for AstraZeneca (non-financial interest) and Eli Lilly (non-financial interest); a role as trial Chair for Pfizer (non-financial interest); and spouse employment at Sandoz.

Theodoros Foukakis: institutional fees for consultancy to AstraZeneca, Daiichi Sankyo, Gilead and Roche; personal fees for consultancy to Affibody, Pfizer, Novartis, Veracyte, Exact Sciences; honoraria from UpToDate; research funding to institution from Pfizer, AstraZeneca, Novartis and Veracyte.

Jonas Bergh: research funding to institution from Amgen, AstraZeneca, Bayer, Merck, Pfizer, Roche and Sanofi; honoraria from UpToDate paid to Asklepios Cancer Research AB; head of advisory board at Stratipath AB; Coronis and Asklepios Cancer Research AB hold shares of Stratipath AB; honoraria for lectures/educational conferences for postgraduates courses from AstraZeneca paid to Coronis and Asklepios Cancer Research AB.

All the other authors had no potential conflicts of interest to disclos

## Appendix A Supplementary data

Supplementary material

Protocol
