# Supplementary material for: Benefit from dose-dense adjuvant chemotherapy for breast cancer: subgroup analyses from the randomised phase 3 PANTHER trial
Source: Lancet Reg Health Eur. 2024 Dec 3;49:101162. doi: 10.1016/j.lanepe.2024.101162 (PMC11652897; doi:10.1016/j.lanepe.2024.101162)

**Supplementary material to**

**Benefit from dose dense adjuvant chemotherapy for breast cancer: secondary analyses from the randomized phase 3 PANTHER trial**

Alexios Matikas, Andri Papakonstantinou, Sibylle Loibl, Günther G. Steger, Michael Untch, Hemming Johansson, Nikolaos Tsiknakis, Mats Hellström, Richard Greil, Volker Möbus MD, Michael Gnant, Jonas Bergh^#^, Theodoros Foukakis ^#^

# JB and TF contributed equally to this work

**Table of Contents**

**Table S1.** Patient characteristics according to clinical composite risk score**2**

**Table S2.** Patient characteristics per treatment arm and with PREDICT estimate**3**

**Table S3.** Cumulative incidence of deaths at 10 years per treatment arm**4**

**Table S4.** Patient characteristics according to menopausal status**5**

**Table S5.** Treatment effect over time**6**

**Table S6.** Discriminatory accuracy of PREDICT**7**

**Figure S1.** Cumulative risk curves for BCRFS according to clinical composite risk score**8**

**Figure S2.** CONSORT diagram**9**

**Figure S3.** Patient flowchart describing disposition according to menopausal status**10**

**Figure S4.** Kaplan-Meier curves for BCRFS according to menopausal status**11**

**Figure S5.** Sensitivity analysis for STEPP**12**

**Figure S6.** Plots of failure rates for time-to-event endpoints**13**

**Figure S7.** Predicted by PREDICT and observed 5-year OS**14**

| **Supplementary Table 1.** Distribution of clinical factors in groups according to clinical composite risk score | | | | |
| --- | --- | --- | --- | --- |
|  | Group 1 (-1.12 to -0.25) | Group 2 (-0.25 to 0.29) | Group 3 (0.29 to 0.95) | Group 4 (0.95 to 1.89) |
| **N** | 453 | 476 | 515 | 545 |
| **Age**  <50  ≥50 | 186 (41.1%)  267 (58.9%) | 254 (53.4%)  222 (46.6%) | 221 (42.9%)  294 (57.1%) | 270 (49.5%)  275 (50.5%) |
| **Nodal status**  0-3  ≥4 | 453 (100%)  0 (0%) | 451 (94.7%)  25 (5.3%) | 238 (46.2%)  277 (53.8%) | 57 (10.5%)  488 (89.5%) |
| **Tumor size**  0-20  >20 | 429 (94.7%)  24 (5.3%) | 162 (34%)  314 (66%) | 210 (40.8%)  305 (59.2%) | 20 (3.7%)  525 (96.3%) |
| **ER and PR**  Positive  Negative | 427 (94.3%)  26 (5.7%) | 387 (81.3%)  89 (18.7%) | 398 (77.3%)  117 (22.7%) | 168 (30.8%)  377 (69.2%) |
| **HER2**  Positive  Negative | 346 (76.4%)  107 (23.6%) | 80 (16.8%)  396 (83.2%) | 121 (23.5%)  394 (76.5%) | 33 (6.1%)  512 (93.9%) |
| **Grade**  1-2  3 | 359 (79.2%)  94 (20.8%) | 294 (61.8%)  182 (38.2%) | 179 (34.8%)  336 (65.2%) | 270 (49.5%)  275 (50.5%) |

Abbreviations: ER: estrogen receptor; PR: progesterone receptor; HER2: human epidermal growth factor receptor

| **Supplementary Table 2.** Baseline characteristics of patients included in the intention-to-treat population of PANTHER and of patients with available PREDICT estimate | | | |
| --- | --- | --- | --- |
|  | **Tailored Dose-Dense chemotherapy ITT (%)** | **Standard chemotherapy ITT (%)** | **Patients with PREDICT estimate (%)** |
|  | **n=1001** | **n=1002** | **n=1961** |
|  |  |  |  |
| **Median age (range)** | 51.1 (23.3 – 69.2) | 50.7 (21.4 – 68.6) | 50.9 (21.4 – 69.2) |
|  |  |  |  |
| **Tumor size (cm)** |  |  |  |
| ≤2 | 413 (41.3) | 413 (41.2) | 810 (41.3) |
| 2 – 5 | 507 (50.6) | 520 (51.9) | 1010 (51.5) |
| >5 | 76 (7.6) | 68 (6.8) | 141 (7.2) |
| Missing | 5 (0.5) | 1 (0.1) | 0 |
|  |  |  |  |
| **Number of positive nodes** |  |  |  |
| 0 | 31 (3.1) | 30 (3.0) | 61 (3.1) |
| 1 – 3 | 591 (59.0) | 555 (55.4) | 1122 (57.2) |
| 4 – 9 | 263 (26.3) | 290 (28.9) | 543 (27.6) |
| >9 | 116 (11.6) | 127 (12.7) | 235 (12.0) |
|  |  |  |  |
| **Tumor grade** |  |  |  |
| 1 | 59 (5.9) | 54 (5.4) | 113 (5.7) |
| 2 | 484 (48.3) | 512 (51.1) | 972 (49.5) |
| 3 | 453 (45.2) | 435 (43.4) | 876 (44.6) |
| Missing | 5 (0.5) | 1 (0.1) | 0 |
|  |  |  |  |
| **Hormone receptor status** |  |  |  |
| ER or PR positive | 805 (80.4) | 795 (79.3) | 1566 (79.8) |
| ER and PR negative | 195 (19.5) | 206 (20.6) | 395 (20.1) |
| Missing | 1 (0.1) | 1 (0.1) | 0 |
|  |  |  |  |
| **HER2 status** |  |  |  |
| Negative | 841 (84.0) | 820 (81.8) | 1624 (82.8) |
| Positive | 160 (16.0) | 182 (18.2) | 337 (17.1) |
|  |  |  |  |
| **Allocated treatment** |  |  |  |
| tDD EC/D | 1001 (100.0) | 0 | 973 (49.6) |
| FEC/D | 0 | 1002 (100.0) | 988 (50.4) |

Abbreviations: ITT: intention-to-treat population; ER: estrogen receptor; PR: progesterone receptor; HER2: human epidermal growth factor receptor

| **Supplementary Table 3.** Cumulative incidence % of deaths at 10 years with corresponding 95% confidence intervals, per treatment. | | | | |
| --- | --- | --- | --- | --- |
|  |  |  |  |  |
| **Factor** | **Group** | **FEC/D** | **tDD EC/D** | **Difference** |
|  |  |  |  |  |
| **All patients** |  | 16.6 (14.3; 19.2) | 15.1 (12.9; 17.7) | -1.5 (-4.9; 1.9) |
|  |  |  |  |  |
| **Age, years** | <50 | 13.9 (11.0; 17.5) | 14.7 (11.5; 18.6) | 0.8 (-4.0; 5.5) |
|  | >50 | 19.1 (15.8; 22.9) | 15.4 (12.4; 19.0) | -3.6 (-8.5; 1.2) |
|  |  |  |  |  |
| **Positive nodes, number** | 0-3 | 11.7 (9.2; 14.7) | 9.9 (7.6; 12.7) | -1.8 (-5.5; 2.0) |
|  | >4 | 23.4 (19.5; 28.1) | 23.8 (19.5; 28.9) | 0.4 (-6.0; 6.8) |
|  |  |  |  |  |
| **Tumour size, mm** | <20 | 10.9 (8.2; 14.6) | 8.6 (6.1; 12.1) | -2.3 (-6.6; 2-0) |
|  | >20 | 20.4 (17.2; 24.1) | 19.7 (16.5; 23.5) | -0.7 (-5.6; 4.2) |
|  |  |  |  |  |
| **Hormone receptor status** | Negative | 27.4 (21.7; 34.3) | 22.5 (17.1; 23.3) | -4.9 (-13.7; 3.8) |
|  | Positive | 13.8 (11.4; 16.5) | 13.2 (10.9; 16.0) | -0.6 (4.2; 3.1) |
|  |  |  |  |  |
| **HER2 status** | Negative | 16.1 (13.6; 18.9) | 16.0 (13.5; 18.9) | -0.1 (-3.9; 3.7) |
|  | Positive | 18.7 (13.5; 25.6) | 10.8 (6.7; 17.0) | -7.9 (-15.7; -0.1) |
|  |  |  |  |  |
| **Tumour grade** | 1-2 | 11.8 (9.3; 15.0) | 12.6 (9.8; 16.1) | 0.7 (-3.5; 5.0) |
|  | 3 | 22.5 (18.7; 26.9) | 18.4 (15.0; 22.5) | -4.1 (-9.6; 1.5) |
|  |  |  |  |  |
| **Ki-67 positive cells, %** | <20 | 12.3 (8.9; 16.9) | 12.2 (8.7; 17.1) | -0.1 (-5.8; 5.6) |
|  | >20 | 19.8 (15.9; 24.6) | 20.2 (16.1; 25.2) | 0.4 (-5.9; 6.6) |
| Abbreviations: FEC: 5-fluorouracil, epirubicin, cyclophosphamide; D: docetaxel; tDD: tailored and dose dense; EC: epirubicin, cyclophosphamide | | | | |

| **Supplementary Table 4**. Baseline characteristics of patients randomized in the PANTHER trial according to baseline menopausal status | | | |
| --- | --- | --- | --- |
|  | **Premenopausal patients**  N=1036 (%) | **Postmenopausal patients**  N=877 (%) | **P value** |
| **Median age, years** (range) | 45.2 (21.4 – 65.3) | 58.6 (26.4 – 69.5) | <0.001 ^1^ |
| **Tumor size, mm** |  | | |
| 0-20 | 424 (40.9) | 354 (50.5) | 0.76 ^2^ |
| 21-50 | 538 (51.9) | 457 (52.1) |  |
| >50 | 69 (6.7) | 66 (7.5) |  |
| Missing | 5 (0.5) | 0 (0.0) |  |
| **Positive nodes** |  | | |
| 0 | 32 (3.1) | 28 (3.2) | 0.48 ^2^ |
| 1-3 | 604 (58.3) | 488 (55.6) |  |
| 4-9 | 284 (27.4) | 244 (27.8) |  |
| >9 | 116 (11.2) | 117 (13.3) |  |
| **Tumor grade** |  | | |
| 1 | 64 (6.2) | 44 (5.0) | 0.003 ^2^ |
| 2 | 480 (46.3) | 463 (52.8) |  |
| 3 | 492 (47.5) | 364 (41.5) |  |
| Missing | 0 (0.0) | 4 (0.5) |  |
| **Hormone receptors** |  | | |
| Positive | 828 (80.0) | 700 (79.8) | 0.96 ^2^ |
| Negative | 207 (20.0) | 176 (20.1) |  |
| Missing | 1 (0.1) | 1 (0.1) |  |
| **HER2 status** |  | | |
| Positive | 185 (17.9) | 141 (16.1) | 0.30 ^2^ |
| Negative | 851 (82.1) | 736 (83.9) |  |
| **Ki-67 %** |  | | |
| ≤20 | 288 (27.8) | 267 (30.4) | 0.03 ^2^ |
| >20 | 373 (36.0) | 270 (30.8) |  |
| Missing | 375 (36.2) | 340 (38.7) |  |
| **Type of operation** |  | | |
| BCS | 518 (50.0) | 502 (57.2) | 0.002 ^2^ |
| Mastectomy | 518 (50.0) | 375 (42.8) |  |
| **Treatment** |  | | |
| tDD EC/D | 515 (49.7) | 441 (50.3) | 0.80 ^2^ |
| FEC/D | 521 (50.3) | 436 (49.7) |  |
| Abbreviations: FEC: 5-fluorouracil, epirubicin, cyclophosphamide; D: docetaxel; tDD: tailored and dose dense; EC: epirubicin, cyclophosphamide; BCS: breast conserving surgery  ^1^ t-test  ^2^ Fisher’s exact test | | | |

| **Supplementary Table 5.** Effect of treatment on primary and secondary endpoints per time period since study inclusion, expressed in hazard ratios and corresponding 95% confidence intervals. Reference in proportional hazard models is standard interval treatment, with HR <1 indicating risk reduction with experimental treatment. | | | | |
| --- | --- | --- | --- | --- |
|  | **BCRFS** | **EFS** | **DDFS** | **OS** |
|  |  |  |  |  |
| **Years 0-3** | 0.74 (0.54 – 1.00) | 0.73 (0.55 – 0.97) | 0.68 (0.49 – 0.95) | 0.79 (0.52 – 1.21) |
| **Years 3-6** | 0.86 (0.58 – 1.29) | 0.82 (0.57 – 1.17) | 0.90 (0.61 – 1.35) | 0.69 (0.47 – 1.02) |
| **Years 6-9** | 0.78 (0.49 – 1.25) | 0.72 (0.48 – 1.09) | 0.74 (0.46 – 1.19) | 1.16 (0.68 – 1.96) |
| **Years 9+** | 0.94 (0.48 – 1.85) | 1.05 (0.60 – 1.84) | 1.22 (0.57 – 2.59) | 0.89 (0.44 – 1.78) |
|  |  |  |  |  |
| **P interaction** | 0.89 | 0.69 | 0.47 | 0.49 |

Abbreviations: BCRFS: breast cancer relapse free survival; EFS: event free survival; DDFS: distant disease-free survival; OS: overall survival

| **Supplementary Table 6.** Discriminatory accuracy of PREDICT model expressed as area under the receiver operator characteristic curve and corresponding 95% confidence interval for 5-year and 10-year overall survival | | |
| --- | --- | --- |
|  | **5-year overall survival** | **10-year overall survival** |
|  |  |  |
| **Entire population** | 0.786 (0.748 – 0.824) | 0.728 (0.696 – 0.760) |
|  |  |  |
| **Country** |  |  |
| Austria | 0.797 (0.733 – 0.860) | 0.766 (0.707 – 0.824) |
| Germany | 0.786 (0.718 – 0.855) | 0.734 (0.675 – 0.792) |
| Sweden | 0.781 (0.720 – 0.841) | 0.704 (0.653 – 0.754) |
|  |  |  |
| **Treatment** |  |  |
| FEC/D | 0.792 (0.743 – 0.842) | 0.740 (0.696 – 0.784) |
| tDD EC/D | 0.777 (0.719 – 0.837) | 0.714 (0.668 – 0.761) |
|  |  |  |
| **Age** |  |  |
| <50 years | 0.796 (0.747 – 0.845) | 0.752 (0.707 – 0.797) |
| ≥50 years | 0.782 (0.725 – 0.839) | 0.704 (0.658 – 0.750) |
|  |  |  |
| **Number of positive nodes** |  |  |
| 0 | 0.703 (0.523 – 0.884) | 0.681 (0.535 – 0.827) |
| 1 – 3 | 0.714 (0.647 – 0.781) | 0.671 (0.618 – 0.724) |
| 4 – 9 | 0.806 (0.746 – 0.867) | 0.683 (0.623 – 0.743) |
| >9 | 0.737 (0.658 – 0.815) | 0.662 (0.588 – 0.735) |
|  |  |  |
| **Tumor size (cm)** |  |  |
| ≤2 | 0.797 (0.714 – 0.880) | 0.752 (0.694 – 0.810) |
| 2 – 5 | 0.725 (0.672 – 0.778) | 0.670 (0.624 – 0.715) |
| >5 | 0.843 (0.776 – 0.910) | 0.789 (0.705 – 0.872) |
|  |  |  |
| **Tumor grade** |  |  |
| 1 | 0.705 (NC – 1.000) | 0.611 (0.359 – 0.863) |
| 2 | 0.742 (0.664 – 0.820) | 0.720 (0.669 – 0.771) |
| 3 | 0.748 (0.698 – 0.798) | 0.699 (0.654 – 0.744) |
|  |  |  |
| **Hormone receptor status** |  |  |
| ER or PR positive | 0.755 (0.702 – 0.808) | 0.711 (0.671 – 0.751) |
| ER and PR negative | 0.744 (0.681 – 0.808) | 0.707 (0.648 – 0.766) |
|  |  |  |
| **HER2 status** |  |  |
| Negative | 0.790 (0.749 – 0.832) | 0.740 (0.705 – 0.774) |
| Positive | 0.791 (0.695 – 0.886) | 0.683 (0.595 – 0.771) |

**Supplementary Figure 1.** Cumulative risk curves for the primary endpoint of breast cancer free survival for the four groups according to clinical composite risk score


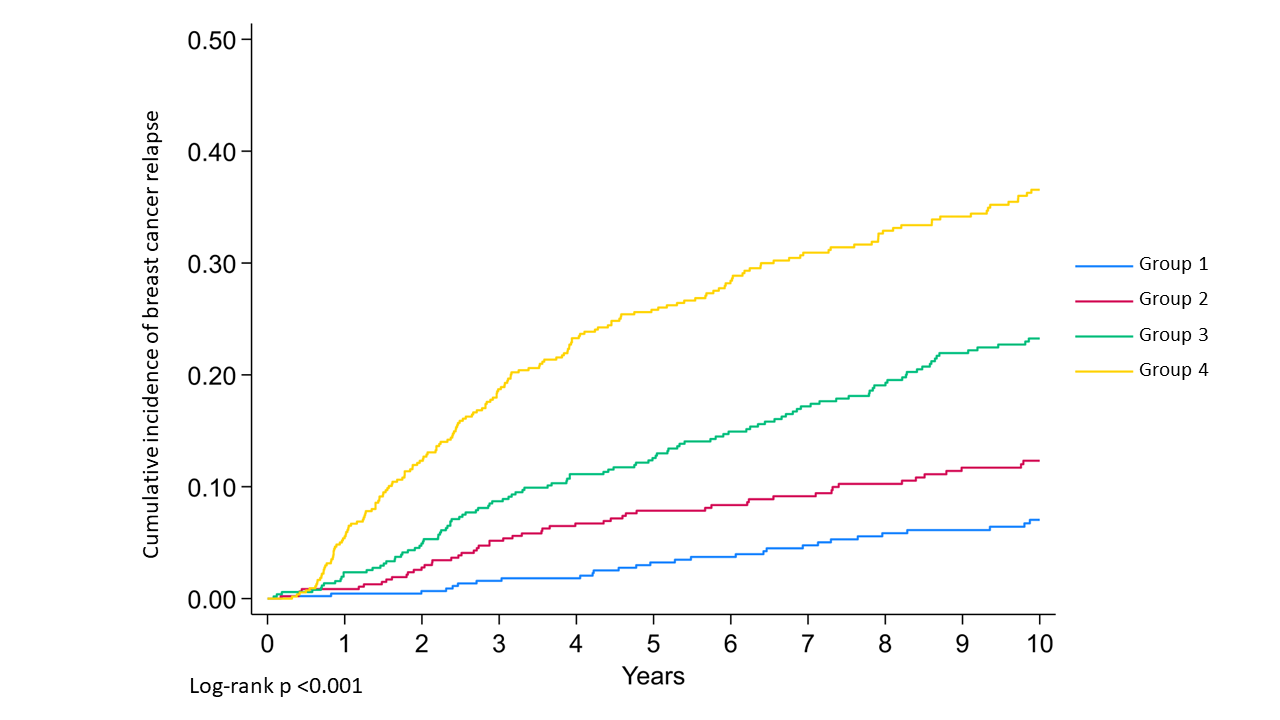


**Supplementary Figure 2.** CONSORT flow diagram of the PANTHER trial showing the 2003 patients who were included in the intention-to-treat analysis. Information on the number of patients screened for eligibility was not collected and is thus not reported.


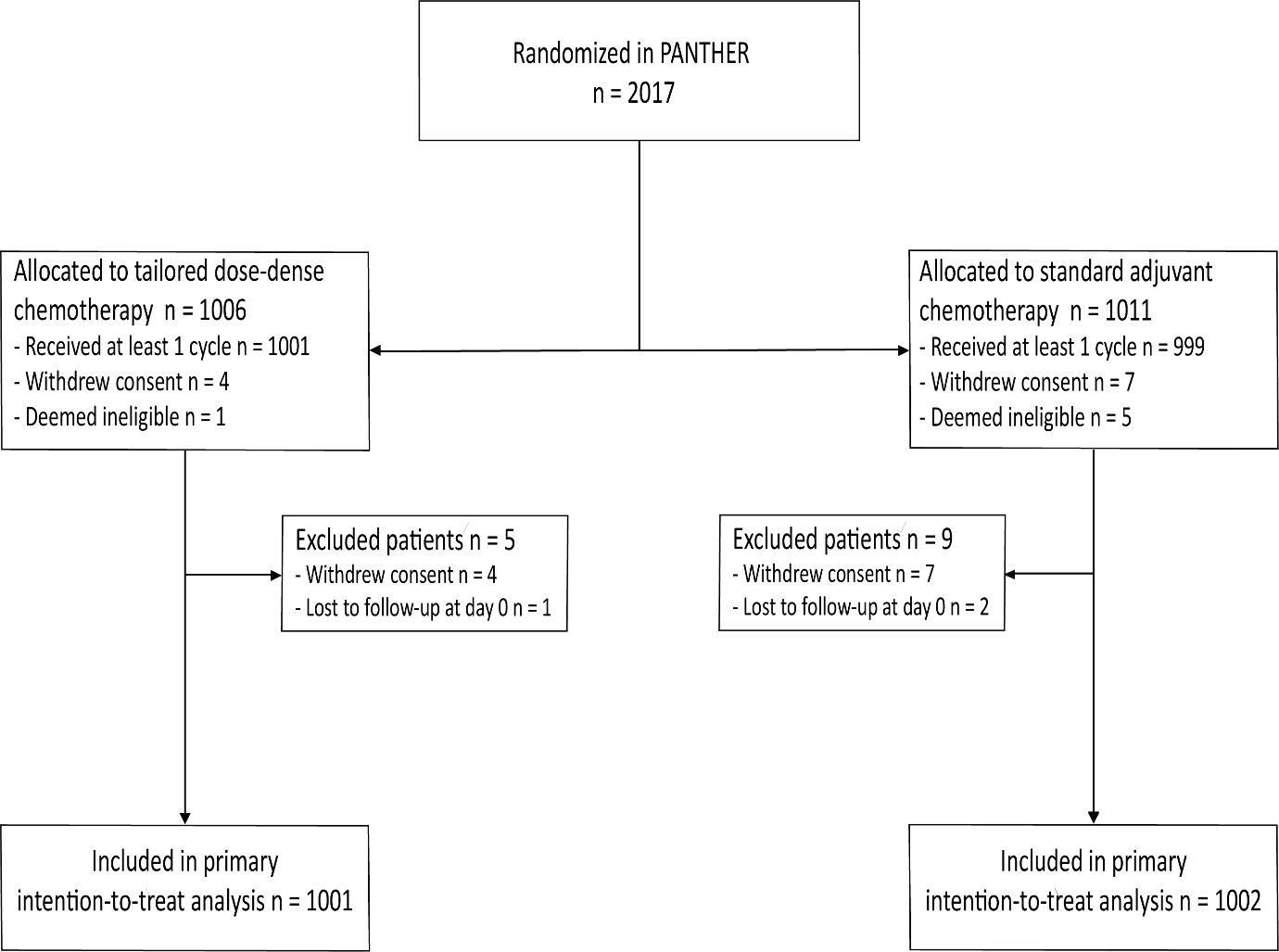


**Supplementary Figure 3.** Patient flowchart of the PANTHER trial describing patient disposition according to menopausal status


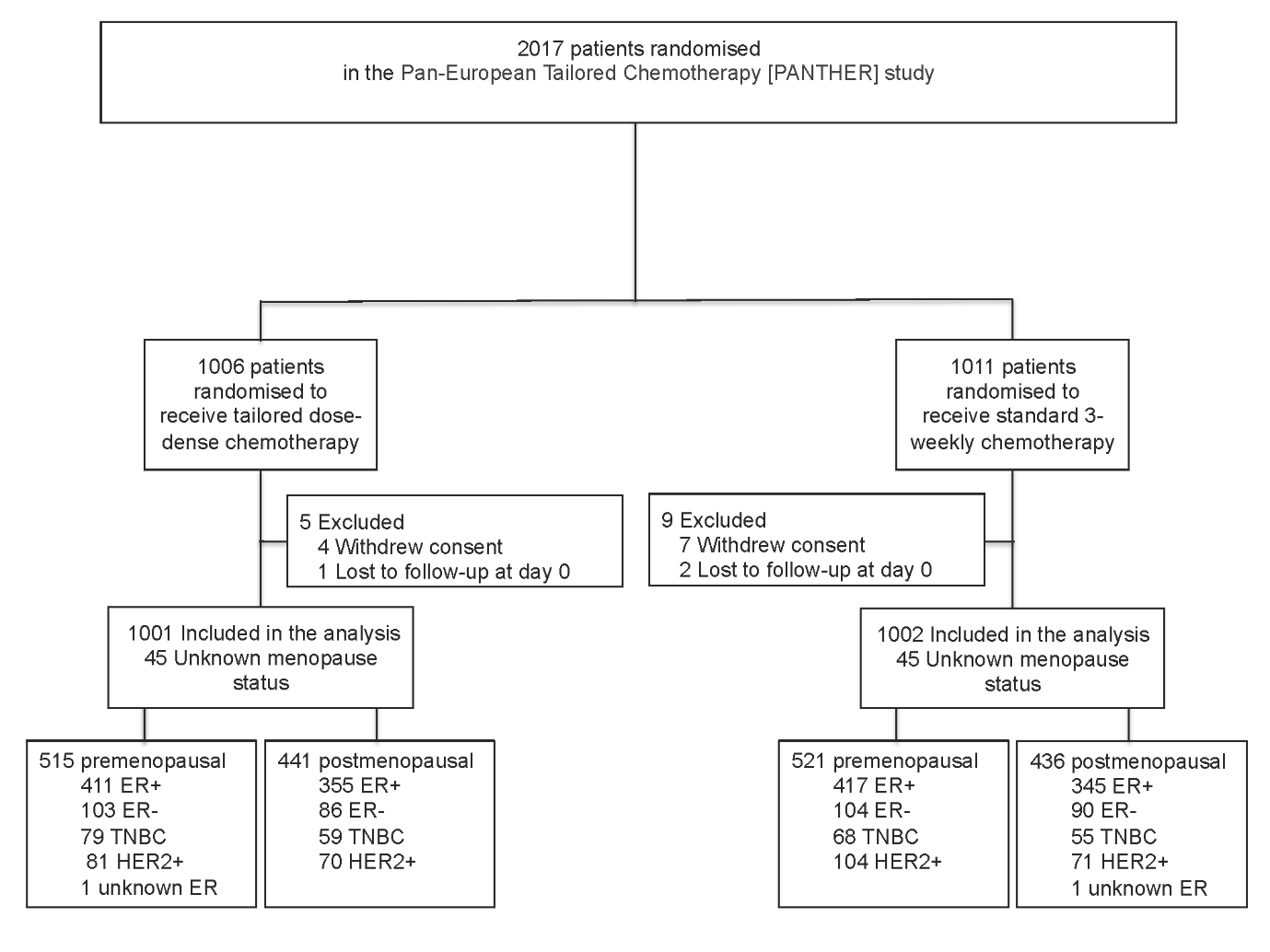


**Supplementary Figure 4.** Cumulative incidence curves of breast cancer relapse free survival events per treatment group and corresponding ten-year event rates in patients with hormone receptor positive breast cancer, premenopausal (A) and postmenopausal (B). Abbreviations: FEC: 5-fluorouracil, epirubicin, cyclophosphamide; D: docetaxel; tDD: tailored dose dense; EC: epirubicin, cyclophosphamide; HR: hazard ratio; CI: confidence interval

**
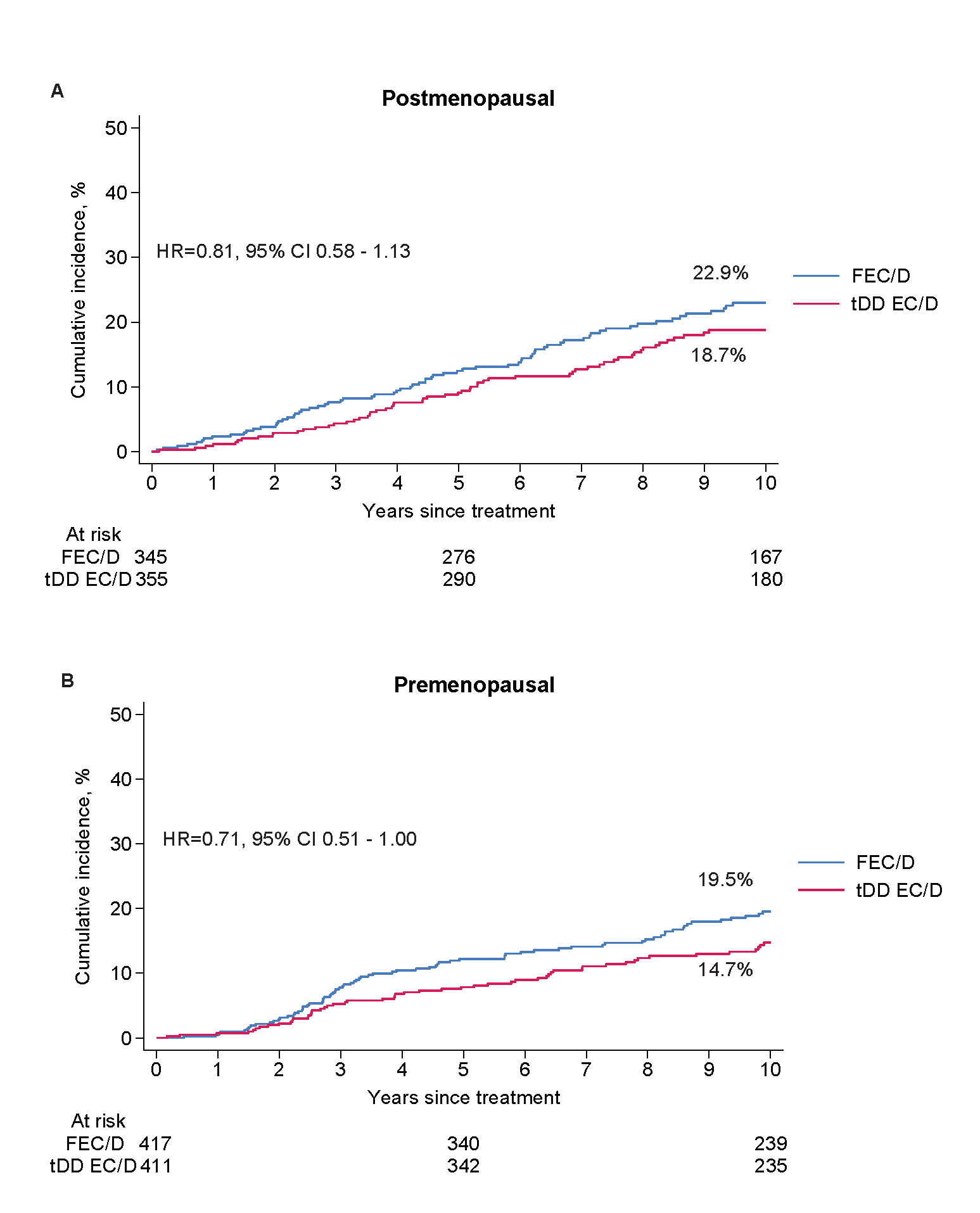
**

**Supplementary Figure 5.** Sensitivity analysis for the subpopulation treatment effect pattern plot (STEPP) by removing HER2 (B) and ER, HER2 (C) from the full model (A). The y axis depicts cumulative incidence of events for the primary endpoint of breast cancer relapse free survival. The x axis depicts continuous clinical composite risk score, from lowest to highest. P value for interaction with treatment arm in the analysis with HER2 removed is 0.180 and in the analysis with ER and HER2 removed 0.210. Abbreviations: FEC: 5-fluorouracil, epirubicin, cyclophosphamide; D: docetaxel; tdd: tailored dose dense; EC: epirubicin, cyclophosphamide

**A B**

**C**

**Supplementary Figure 6.** Plots of failure rates for the endpoints of breast cancer free survival (A; BCRFS), event free survival (B; EFS), distant disease-free survival (C; DDFS) and overall survival (D; OS) per time interval following randomization. Abbreviations: FEC: 5-fluorouracil, epirubicin, cyclophosphamide; D: docetaxel; tdd: tailored and dose dense; EC: epirubicin, cyclophosphamide


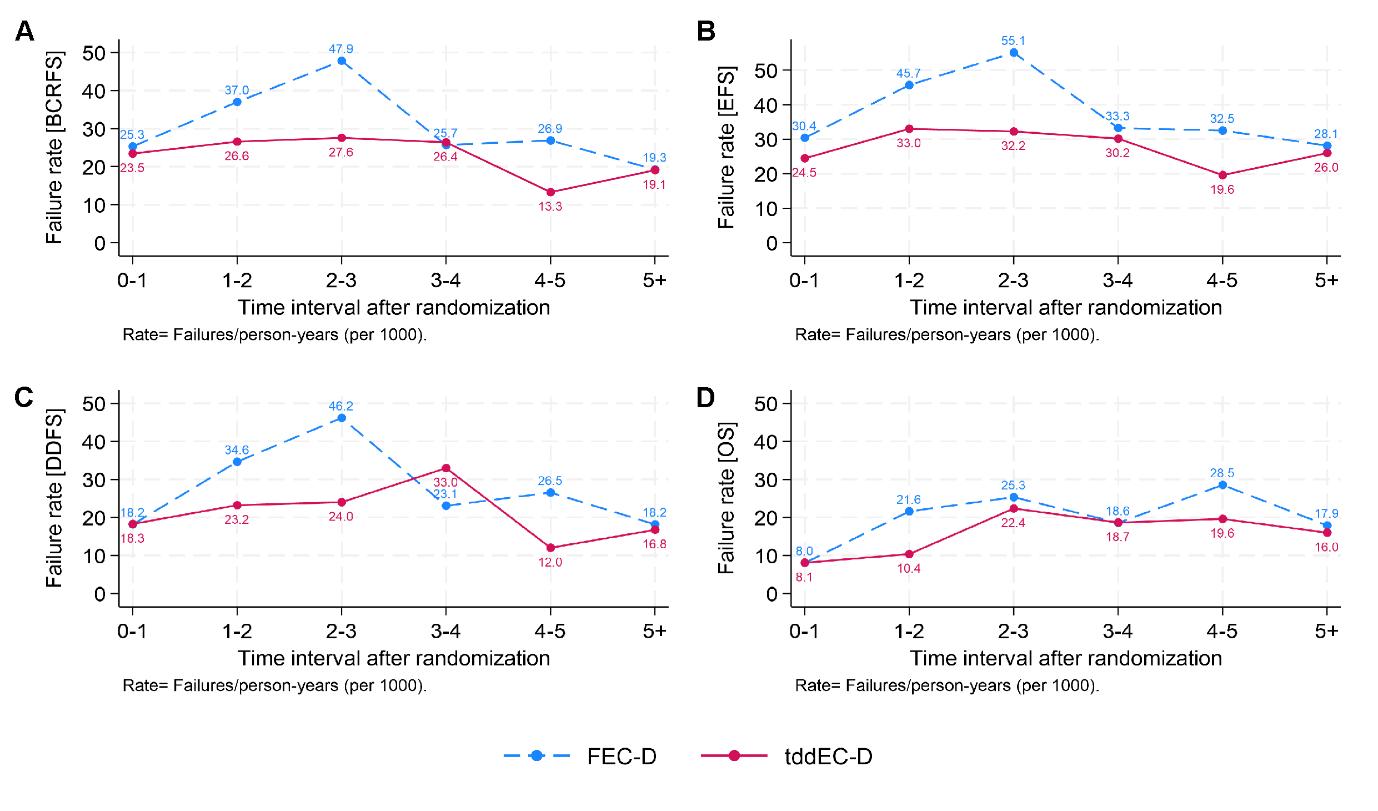


**Supplementary Figure 7.** Estimated according to the PREDICT model and observed overall survival rates at 5 years. Abbreviations: SE: standard error; CI: confidence interval; FEC: 5-fluorouracil, epirubicin, cyclophosphamide; D: docetaxel; tdd: tailored dose dense; EC: epirubicin, cyclophosphamidel HR: hormone receptor; HER2: human epidermal growth factor receptor 2


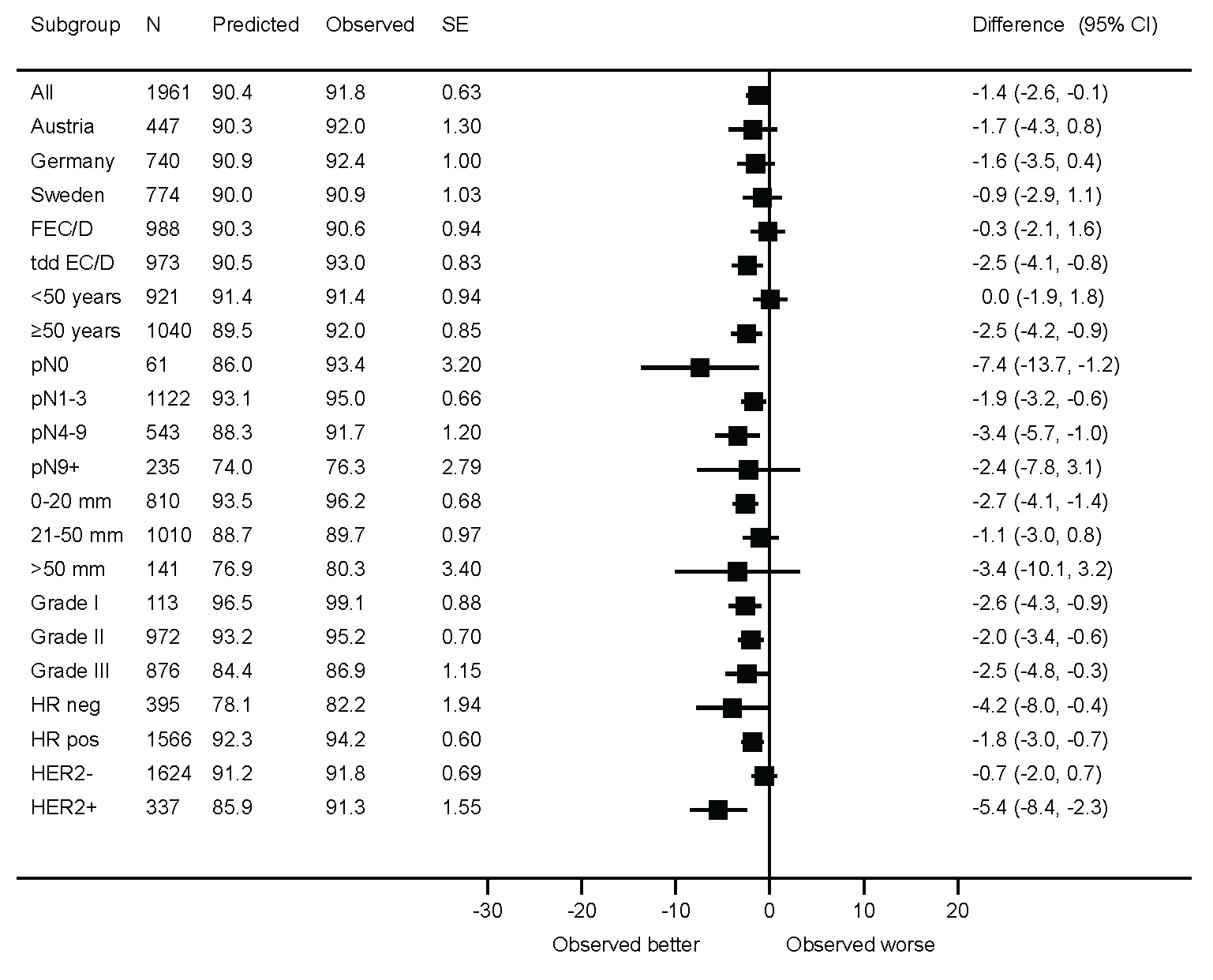

Supplement: Supplementary material [file mmc1.docx]
