# Supplementary material for: Benefit from dose-dense adjuvant chemotherapy for breast cancer: subgroup analyses from the randomised phase 3 PANTHER trial
Source: Lancet Reg Health Eur. 2024 Dec 3;49:101162. doi: 10.1016/j.lanepe.2024.101162 (PMC11652897; doi:10.1016/j.lanepe.2024.101162)
Supplement: Protocol [file mmc2.pdf]

PANTHER

(The SBG2004-1/ABCSG25/GBG53 Protocol)

A randomized phase III study

Comparing biweekly and tailored epirubicin + cyclophosphamide followed by biweekly tailored docetaxel (dtEC→dtT) (A-arm)

versus

three weekly epirubicin + cyclophosphamide, 5-fluorouracil followed by docetaxel(FEC→T) (B-arm)

in lymph node positive or high risk lymph node negative breast cancer patients – a continuation of the feasibility part of the SBG 2004-1 study

EudraCT 2007-002061-12

-- ----------------------------
Investigator Date

Amended protocol 4.10, 27 Dec 2012

Confidentiality Agreement

We respectfully request that you indicate your agreement to treat such information in a confidential manner by signing and returning the duplicate copy of this letter.

I agree to treat information about the Panther protocol in a confidential manner.

Investigators signature Date

Signatures

Prof Jonas Bergh Date
Principal investigator

Principal Investigator / Sponsor 7
Country Study Chairs: 7
Steering Committee 7
List of Investigators SBG (Scandinavian Breast Cancer Group) 7
Study Statisticians 8
Randomization centres 8
Central Data Centre 8
Study offices 9
National Co-ordinating Investigator ABCSG (Austrian Breast & Colorectal Cancer Study Group) . 9
National Co-ordinating Investigator GBG (German Breast Group) 9
1 SUMMARY 10
2 BACKGROUND 11
2.1. Introduction 11
2.2 Dose-escalated and tailored FEC 11
2.3 High-dose chemotherapy with stem cell support 13
2.4 Dose response - epirubicin 13
2.5 Dose response – cyclophosphamide 13
2.6 Taxanes – docetaxel and paclitaxel 14
2.7 Dose-dense therapy 16
2.8 Data from the feasibility part of the SBG 2004-1 study 16
2.9. Motives for the present design of this phase III study 19
2.10. G-CSF (Granulocyte colony stimulating factor) 19
3 OBJECTIVES 20
3.1 Phase III 20
3.1.1 Primary objective 20
3.1.2 Secondary objectives 20
3.2. Additional aims/ Biological markers 20
4 STUDY DESIGN 21
4.1. Arm A: Two weekly dtEC→dtT (tailored and dose escalated dtEC x 4 + tailored and dose-escalated docetaxel x 4) 22
4.2. Arm B: Three weekly FEC→T (FEC x 3 followed by docetaxel x 3) 22
5 STATISTICAL CONSIDERATIONS 22
5.1 Phase III 22
5.2.1 Endpoints 22
5.2.2 Choice of treatment arms and number of patients 23
5.3 Analysis sets 24
5.4 Statistical analyses 24
6 PATIENT ELIGIBILITY 24

6.1 Inclusion criteria 24
6.2 Exclusion criteria 25
7 PATIENT INCLUSION AND RANDOMIZATION 25
7.1. Patient information and consent 25
7.2 Randomization 26
8 THERAPY AND STANDARD PROCEDURES BEFORE RANDOMIZATION 26
8.1 Baseline investigations 26
8.2 Surgical treatment 26
8.3 Histopathology and immunohistochemistry 27
9 DOSAGE AND ADMINISTRATION OF THERAPY 27
9.1 Arm A: biweekly dtEC→dtT - Dose escalated and tailored EC x 4 (three weeks break) followed by biweekly dose-escalated and tailored docetaxel x 4 27
9.1.1 G-CSF 28
9.1.2 Dose-modifications - dtEC 28
9.1.3 Dose dense and tailored docetaxel x 4 (after dtEC x 4) 29
9.2 Arm B: Three weekly fixed dosed FEC→T – $FE_{100}C$ x 3 followed by three weekly docetaxel x 3 (100 mg/m^2 without escalation) 31
9.2.1 Dose-modifications - FEC→T 32
9.3 Administration of cytotoxic drugs 33
9.4.1. dtEC→dtT (A arm) 33
9.4.2. FEC→T (B arm) 34
9.5 Auxiliary therapy 34
9.6 Postoperative radiation 34
9.7 Hormonal therapy 34
9.8 Trastuzumab 35
9.9 Other anti-cancer agents 35
9.10 Assessment of health related quality of life 35
10. PHARMACOLOGICAL INFORMATION 35
10.1. Pharmacological information 35
10.1.1. Epirubicin 35
10.1.2 Cyclophosphamide 36
10.1.3 5-fluorouracil 37
10.1.4 Docetaxel 37
10.1.5 Filgrastim (Neupogen®), pegfilgrastim (Neulasta®) 38
11 EVALUATION AND MONITORING 38
11.1 Case report forms 38
11.2 Assessments before treatment 38
11.3 Assessments during treatment 39
11.4 Toxicity evaluation 39
11.5 Follow up 39
11.6. Safety 39

11.7. Safety measurements 39
11.8. Adverse event reporting 40
11.9 Monitoring 41
12. COLLECTION OF DATA AND CONFIDENTIALITY 41
12.1. Collection of data 41
12.2. Confidentiality of trial documents and patients records 41
13 ETHICS 41
14 PUBLICATION POLICY 42
14.1. Publication 42
15. INDEPENDENT DATA SAFETY AND MONITORING COMMITTEE 42
16. REFERENCES 42
17. APPENDIX 46
Appendix 1. Investigations 47
Appendix 2. Recommendations for grading of acute and subacute toxicity 49
Appendix 3. New York Heartt Association Classification of Congestive Heart Failure 50
Appendix 4. Declaration of Helsinki 51
Appendix 5 ASSESSMENT OF HEALTH RELATED QUALITY OF LIFE 54

	Department	Telephone/ *Fax* and Email

Principal Investigator / Sponsor

Jonas Bergh<br>MD, PhD, Professor	Department of Oncology,<br>Karolinska University Hospital,.<br>SE-17176 Stockholm	+46 8 51776279/+46 8 51779524<br>jonas.bergh@ki.se

Country Study Chairs:

Jonas Bergh<br>MD, PhD, Professor	Department of Oncology,<br>Karolinska University Hospital, SE-17176 Stockholm.	+46 8 51776279/+46 8 51779524<br>jonas.bergh@ki.se
Michael Gnant<br>MD, Professor	Department of Surgery, Medical University<br>Vienna, A-1090 Vienna, Austria	+43 1 40 400 5646<br>michael.gnant@meduniwien.ac.at
Gunter von Minckwitz<br>MD, PhD, Professor	University Womens' Hospital Frankfurt/Main<br>c/o GBG Forschungs GmbH<br>Schleussnerstrasse 42,<br>D-63263 Neu-Isenburg, Germany	+49 6102 7480 0<br>gunter.vonminckwitz@germanbreastgroup.de

Steering Committee

Jonas Bergh, head	Karolinska University Hospital, Stockholm, Sweden
Per Malmström	Lund University Hospital, Lund, Sweden
Nils-Olof Bengtsson	Norrlands University Hospital, Umeå, Sweden
Michael Gnant	Medical University, Wien, Austria
Richard Greil	Paracelsus University, Salzburg, Austria
Gunter von Minckwitz	University Womens' Hospital Frankfurt/Main, Germany
Volker Möbus	Städt. Kliniken, Frankfurt-Höchst, Germany

List of Investigators SBG (Scandinavian Breast Cancer Group)

Investigators

Henrik Lindman<br>MD, PhD	Department of Oncology, Uppsala University<br>Hospital, SE-51785 Uppsala	+ 46 18 611 00 00
Lena Carlsson<br>MD	Department of Oncology<br>Central Hospital, Sundsvall	+ 46 60 18 10 00<br>lena.carlsson@vnl.se
Barbro Linderholm<br>MD, PhD,	Department of Oncology, Karolinska<br>University Hospital, Stockholm.	+ 46 8 517 762 79/+46 8 517 751 96<br>barbro.linderholm@ki.se
Sam Rotstein<br>MD, PhD,	Department of Oncology, Karolinska<br>University Hospital/Danderyd Hospital	+ 46 8 655 50 00
Per Malmström<br>MD, PhD	Department of Oncology, University Hospital<br>Lund, Sweden	+46 46 177 520 /+46 46 176 080<br>per.malmstrom@onk.lu.se
Martin Söderberg<br>MD	Department of Oncology, University Hospital<br>Malmö	+46 40 331 000/<br>martin.soderberg@skane.se
Nils-Olof Bengtsson<br>MD	Department of Oncology, University Hospital<br>Umeå, Sweden	+ 46 90 785 00 00

Stig Holmberg<br>MD, PhD	Department of Surgery, Sahlgrenska University/<br>Mölndal Göteborg	+ 46 31 342 10 00
Per Karlsson<br>MD, PhD	Department of Oncology, Sahlgrenska University<br>Göteborg	+ 46 31 342 10 00
Zacharia Einbeigi<br>MD, PhD	Department of Oncology, Sahlgrenska University<br>Göteborg	+ 46 31 342 10 00
Tommy Fornander<br>MD, PhD	Department of Oncology, Karolinska University<br>Hospital/ Södersjukhuset Stockholm	+ 46 8 616 10 00
Charlotta Dabrosin<br>MD, PhD	Department of Oncology, University Hospital<br>Linköping	+ 46 13 22 20 00
Kenneth Villman<br>MD	Department of Oncology, University Hospital<br>Örebro	+ 46 19 602 10 00
Johan Ahlgren<br>MD, PhD	Department of Oncology, Central Hospital<br>Gävle-Sandviken	+ 46 26 15 40 00
Per Edlund<br>MD, PhD	Department of Oncology, Central Hospital<br>Gävle-Sandviken	+ 46 26 15 40 00
Eva Karlsson<br>MD	Department of Onclolgy, Central Hospital<br>Karlstad	+ 46 54 61 50 00

Study Statisticians

Harald Anderson<br>PhD.	Oncology Center, Lund University Hospital,<br>SE-22185 Lund, Sweden.	+46 46 17 75 61 /+46 46 18 81 43<br>harald.anderson@onk.lu.se
Christian Fesl<br>PhD.	Trial Office, ABCSG<br>A-1090 Vienna, Austria	+43 1 408 92 30<br>ernst.ruecklinger@abcsg.at

Randomization centres

Scandinavia	Clinical Trials Unit, Dept of Oncology<br>Karolinska University Hospital, SE-17176 Stockholm	Fax + 46 8 30 69 89
Austria	https://www.muw.ac.at/randomizer/web/login.php	
Germany	GBG Forschungs GmbH<br>Schleussnerstrasse 42<br>D-63263 Neu-Isenburg<br>Germany	+49 6102 7480-0<br>Fax: +49 6102 7480-440

Central Data Centre

Scandinavia	Central Data Centre/ Clinical Trials Unit,<br>Dept of OncologyFax + 46 8 30 69 89<br>Karolinska University Hospital, SE-17176 Stockholm	+46 8 51 77 36 77

Study offices

Sweden	Kliniska prövningsenheten<br>Dept of Oncology, Karolinska University Hospital<br>SE-171 76 Stockholm, Sweden	+46 8 +51 77 36 77
Austria	Austrian Breast & Colorectal Cancer Study Group<br>Nussdorfer Platz 8<br>AT-1190 Wien, Austria	+43 1 408 92 30
Germany	German Breast Group Forschungs GmbH<br>Martin-Behaim-Str. 12<br>DE-63263 Neu-Isenburg, Germany	+49 6102 7480-0

National Co-ordinating Investigator ABCSG (Austrian Breast & Colorectal Cancer Study Group)

	**Department**	**Telephone/Email**
Richard Greil<br>MD, Professor	Department of Internal Medicine III<br>Paracelsus University<br>A-5020 Salzburg, Austria	+43 662 4482 2881<br>r.greil@salk.at

National Co-ordinating Investigator GBG (German Breast Group)

	**Department**	**Telephone/ *Fax* and Email**
Volker Möbus<br>MD, Professor	Städt. Kliniken Frankfurt-Höchst<br>Frauenklinik<br>Gotenstrasse 6-8<br>D-65929 Frankfurt/Höchst, Germany	+ 49 69 3106-2355 o. -2339<br>studien-frauenklinik@skfh.de

Protocol writing: Jonas Bergh, Barbro Linderholm, Nils Wilking, Thomas Linné (feasibility and phase II). Jonas Bergh, Michael Gnant (phase III).

1 SUMMARY

This is an adjuvant, open, prospective, randomized study to compare

A. Individually tailored and two weekly dosed epirubicin + cyclophosphamide followed by a three weeks break followed by biweekly and tailored docetaxel (dtEC→dtT) given every second week to
B. Fixed dosed and three weekly epirubicin, cyclophosphamide and 5-fluorouracil, followed by fixed dosed and three weekly docetaxel (FEC→T)

G-CSF support is used to allow the two weekly strategy.

Patients with primary node-positive or high risk lymph node negative breast cancer will be eligible for the study.

Duration of enrolment is planned to be 4.5 years, starting January/February 2007 for this phase III part.

The primary objective of the phase III study is to compare breast cancer relapse-free survival (BCRFS) between the dtEC→dtT and $FE_{100}C$→T. To detect a five-year BCRFS difference of 0.710 to 0.790 about 762 patients per arm will be needed. They will be recruited during three years and followed another two years for breast cancer events.

In August 2010 the planned interim analysis of the average event-rate was performed, based on about 1200 patients included until May 2010. The observed event-rate was somewhat lower than assumed in the dimensioning of the study. Thus it was decided to increase the number of patients to n=2000, and this number will be reached in mid 2011. According to the interim analysis the chance is then good to reach the goal of 225 primary events in December 2012.

Secondary objectives are to compare

1: Distant disease-free survival (DDFS)
2: Event-free survival and
3: Overall survival
4: Health-related quality of life and toxicity analyses according to CTC
5: Outcome in relation to tumour biological factors and polymorphism patterns
   a. RFS in relation to the Sorlie classes using immunohistochemical markers and/or gene expression profiling comparing A vs B arm.
   b. RFS with receptor positive disease (analyzed in the local laboratories as described in the CRFs and also analyzed as continuous variables) in the comparison between the A- and B-arms.
   c. RFS with high and low proliferation, respectively, (analyzed in the local laboratories as described in the CRFs and also analyzed as a continuous variable, or centrally analyzed), in the comparison between the A- and B-arms.
   d. RFS in relation to HER-2/neu status (analyzed in the local laboratories as described in the CRFs) in the primary cancers in the comparison between the A- and B-arms and anlyzed whether trastuzumab was given in sequence or concurrently.
   e. RFS analyzed in relation to other molecular markers (e.g. gene expression profiling/ sequencing) in the primary cancers and SNPs signatures in normal DNA (related to toxicities for EC/FEC and docetaxel components, respectively, and given dose levels and outcome in relation to these factors and in relation QoL) to outcome per arm.

f. RFS analyzed in relation to tumour associated lymphocytes and Y-box binding protein in the comparison between the A- and B-arms.

Tumour tissue will be obtained and stored for studies of prognostication and therapy prediction.

2 BACKGROUND

2.1. Introduction

Adjuvant poly chemotherapy improves overall survival. The absolute benefit is 8% to 12%, corresponding to a relative mortality reduction of 16% to 24%, at 10 years (1). The addition of tamoxifen to chemotherapy in patients with receptor positive tumours, further enhances the absolute effect with 4% to 8% (1, 2). The use of anthracycline based regimens in comparison with CMF (cyclophosphamide, methotrexate, 5 fluorouracil) regimens result in absolute survival benefit of 3% for the former (1). The effect by chemotherapy was essentially maintained at the longer follow-up (3). The addition of postoperative radiotherapy added a small but statistically significant survival gain even for patients receiving radiotherapy after breast-conserving surgery (4).

In the present study, it will take about 6 years, or more, from study design until analysis and presentation, and a high-rank aim is to design a study that answers research questions that are of interest at that time. Ideally there is a standard control arm and an experimental arm in a clinical study. This type of classical design is also much more attrative in research areas where the end-point(s) can be reached in a shorter time frame. Due to the rapid development in breast cancer medical oncology research it is impossible to know what will be considered standard treatment in 6 years time. At present, many therapy options can be considered as reasonable standards, in different parts of the world.

Taxane based adjuvant regimens have resulted in fewer breast cancer relapses, in four studies statistically significant survival improvements have been recorded (5-8). This is the motive for inclusion of a taxane in both therapy arms (A, B). However, these studies may not have included the most optimal anthracycline based regimens as comparators, e.g. four courses of AC (doxorubicin, cyclophosphamide) was compared with four courses of AC followed by four additional courses of paclitaxel (5).

The design of the present study is aiming at having therapy options, which also should be modern, and up-to date also at time of analysis of the phase III part of the study.

2.2 Dose-escalated and tailored FEC

Higher doses of chemotherapy in the conventional dose range for patients with metastatic breast cancer are almost invariable associated with a higher response rate (9), while the efficacy on survival is marginal (10). Too low doses in the adjuvant setting are associated with a worse outcome (11-14).

Marked inter patient variation has been documented for the compounds in the FEC regimen, despite that doses had been adjusted to the patients body surface area (15-17). Six retrospective studies have revealed an inferior outcome for patients receiving adjuvant chemotherapy without toxicity, compared with those who experienced haematological toxicity, in one study moderate (grade 2-3) granulocyte toxicity (14, 18-21). These data were not confirmed in one study (22).

This concept, increasing chemotherapy dose to patients without toxicity, has recently been tested in the SBG 2000-1 study comparing tailored FEC without G-CSF versus standard Scandinavian $F_{600}E_{60}C_{600}$ for seven courses. 1535 patients became included before the closure of the study in August 2003. Randomised data, as described below, however indicate that this $F_{600}E_{60}C_{600}$ regimen may be suboptimal, with reference to the used epirubicin dose.

Initial and uncontrolled pilot and phase I/II marrow supported high dose chemotherapy studies strongly indicated a survival gain compared with historical controls (9). The SBG 9401 study was initiated for women with a very high risk for relapse using conventional adjuvant chemotherapy. The study compared individually tailored and dose escalated FEC (5-fluorouracil, epirubicin, cyclophosphamide) regimen for 9 courses versus standard FEC x 3 followed by high dose therapy with CTCb (carboplatin, thiotepa, cyclophosphamide) (9). Patients who were included had an estimated 70% risk or more for relapse within 5 years, derived from previous Scandinavian breast cancer registries. Six dose levels were used in the dose escalated FEC arm, epirubicin (38 mg/m^2 to 120 mg/m^2) and cyclophosphamide (450 mg/m^2 to 1800 mg/m^2) while 5-fluorouracil was given in a standard dose of 600 mg/m^2. Patients received the first FEC course at the doses 600 mg/m^2, 75 mg/m^2 and 900 mg/m^2. Based on the haematological toxicity day 8, 11/12 and 15 at each course the following courses were adjusted; escalated, unchanged dose or reduced (9). Patients in both treatment arms received loco-regional radiotherapy and tamoxifen for 5 years.
Five hundred and twenty-five patients were randomised during 4 years from 1994 to 1998. All analyses were run according to the intention to treat principle. With a median follow-up of 34.3 months 81 breast cancer relapses were recorded in the dose escalated FEC arm, compared to 113 breast cancer relapses in the CTCb arm. This difference was significant according to the double triangular model (p=0.0439) (23). With respect to overall survival, with a median follow-up of 38.3 months, 60 deaths were recorded in the dose escalated FEC arm and 82 in the CTCb arm, this difference was not significant (p=0.12).
In a retrospective analysis we calculated the event-free survival. As an event we included breast cancer relapse, contra lateral breast cancer, and all other malignancies as an event. Ninety-five and 118 events were recorded in the dose escalated FEC arm and CTCb arm, respectively (9). The down side for the dose escalated FEC arm was that 10 patients developed AML (acute myeloid leukaemia) and/or MDS (myelodysplastic syndrome), respectively (9, 24). The dose escalated FEC regimen was given with G-CSF support from day 2 to 14 for 9 courses. Six of the 10 patients developed AML/MDS within 24 months from start of chemotherapy.
The estimate for AML/MDS in the SBG 9401 study is within the same confidence interval as for the Canadian study using an intensified FEC regimen, 60 mg/m^2 of epirubicin day 1 and 8, for 6 courses without use of G-CSF (25). The SBG 9401 study has been updated, still revealing significantly fewer breast cancer-relapses in the tailored and dose escalated FEC arm, but with no differences in event-free survival or overall survival (26). During the follow-up time 2000 to the spring of 2003 one further patient was recorded with AML/MDS (24). A tailored and dose escalated FEC was one of the available FEC therapy options in the recently closed EORTC/p53 study, by November 2006 121 patients have been randomised to this regimen since 2002. The total number of courses has been reduced to 6 and the maximal cyclophosphamide dose per course has been reduced to 1200 mg/m^2 and G-CSF is only delivered from day 5-12. By November 2006 we have so far no report of AML/MDS in the Swedish part of this study, although one patient has been diagnosed with a mantle cell lymphoma.
This could indicate that the risk of AML/MDS with a modified and tailored and dose escalated FEC regimen would be in the same ratio as studies using anthracyclins in higher doses giving a better breast cancer outcome compared with CMF or lower doses of anthracyclins (25, 27).

The tailored and dose escalated EC regimen in this study has been slightly further modified compared with the EORTC/p53 study. The starting dose of cyclophosphamide has decreased from 900 mg/m^2 to 600 mg/m^2. The starting dose of epirubicin has been increased from 75 mg/m^2 to 90 mg/m^2. 5-flurouracil has also been removed from the regimen based on most recently published data (28).

2.3 High-dose chemotherapy with stem cell support

Around 6000 patients have been randomised into studies using marrow supported high dose therapy compared with standard chemotherapy or other comparators like dose escalated and tailored FEC (29). One study has so far demonstrated a survival gain for the marrow-supported high dose procedure (30). Subgroup analyses have revealed that patients with HER-2/neu negative tumours may have benefit from this type of strategy, at least compared with five courses of standard $F_{500}E_{90}C_{500}$ (31).

These studies have had marrow-supported high dose therapy related mortality ranging from 0.7% in the SBG 9401 study (9) to 4% (<40 years) to 14% (32). In a recently reported adjuvant randomised study consisting of 540 patients, 9 patients died of the high dose procedure and 9 also developed AML/MDS in the marrow supported high dose arm (33).
In conclusion, these data further underline the importance of randomised data; uncontrolled (pilot/phase I-II) data strongly indicated the superiority of adjuvant marrow-supported high dose therapy (9, 34). Finally, the present data are not supporting further exploration of the marrow-supported high dose strategy for breast cancer.

2.4 Dose response - epirubicin

The French adjuvant study group has demonstrated an absolute five year survival gain of 12% for patients receiving epirubicin at 100mg/m^2, compared with 50 mg/m^2 in the FEC regimen (27). This study consisted of 585 primary breast cancer patients with 4 or more positive lymph-nodes, or 1-3 positive lymph-nodes and Scarff Bloom Richardson > 2 and hormone receptor negativity. The benefit in this study was restricted to patients with 4 or more lymph-node metastasis. The benefit of a higher epirubicin dose has also been demonstrated in another randomised epirubicin containing adjuvant study (35).

2.5 Dose response – cyclophosphamide

Conventional dose-escalation of cyclophosphamide up to single doses of 2400 mg/m^2 has not resulted in overall survival gains (36, 37). However, subgroup analyses have revealed potential positive effects for younger patients with 4 to 9 positive axillary nodes. Moreover the results from these studies indicate that higher doses of cyclophosphamide may increase the risk of development of secondary leukaemia, whereby in the present protocol, escalation of cyclophosphamide is only permitted to a dose of 1200 mg/m^2 per course.

2.6 Taxanes – docetaxel and paclitaxel

Docetaxel is an effective chemotherapeutic agent for advanced breast cancer with significant activity as first-line therapy as well as second-line therapy in patients refractory to anthracyclines (38-42).
The results from a study, which accrued 1 491 patients, investigated the effect of docetaxel in the adjuvant setting was recently published, demonstrating statistically significantly fewer breast cancer relapses for docetaxel, doxorubicin and cyclophosphamide (TAC) compared with fluorouracil, doxorubicin and cyclophosphamide (FAC) in primary node-positive breast cancer (7). This was the 2nd interim analysis at a medium follow-up of 55 months with 399 DFS events. For the primary endpoint, DFS, there was a statistically significant advantage for TAC over FAC at this time (p=0.001). OS also favoured TAC over FAC (p=0.008). Exploratory sub-group analyses were also performed at the time of the interim analysis; although these analyses are powered for 590 events. However, with only 399 events observed thus far, DFS advantages with TAC were observed for patients with 1-3 positive lymph nodes (p=0.0009), hormone receptor positive (p=0.0076) and negative tumours (p=0.0297), and HER-2 positive (p=0.0088) and negative tumours (p=0.046). DFS for patients with 4+ nodes favoured TAC (p=0.17). An interaction test did not suggest that there was a statistically meaningful difference in DFS comparing patients with 1-3 nodes and those with 4 or more nodes. The hazard ratio for those patients with 1 – 3 positive nodes was 0.61 (95% confidence interval 0.46 – 0.82) or for those with 4 or more positive nodes the relative hazard ratio was 0.83 (95% confidence interval 0.63 – 1.08). The hazard ratio for patients with hormone receptor negative and hormone receptor positive disease was in the same range, 0.69 (95% confidence interval 0.49 – 0.97) and 0.72 (95% confidence interval 0.56 – 0.92), respectively. Taken together these data indicate potentially a better benefit for those with 1 – 3 positive nodes and similar effect for those with receptor negative and receptor positive disease.

However the primary endpoint of difference of DFS (adjusted for N status in the ITT-population) between the TAC arm and the FAC arm are strongly significant with a p-value of 0.0010.
The standard dose and administration of docetaxel is 100 mg/m^2 as a one-hour infusion every third week. The dose-limiting toxicity is myelosuppression which is dose but not schedule-dependent (43). In most published studies, a 90 to 95% incidence of grade 3 or 4 neutropenia is reported when docetaxel have been administered at 100 mg/m^2 every third week. Other toxicities include asthenia, skin reactions, nail-disorders, stomatitis, hypersensitive reactions, and a fluid-retention syndrome, which rather effectively can be prevented by administration of corticosteroids. Recently a randomized phase III study was presented demonstrating a statistically significantly survival benefit for docetaxel at 100 mg/m^2 compared with paclitaxel at 175 mg/m^2 for patients with advanced and anthracycline resistant breast cancer (44). The median survival was improved from 12.7 to 15.4 months (p=0.03). However, docetaxel treatment was accomplished with statistically significantly more grade 3 – 4 toxicity; particularly neutropenia, febrile neutropenia, fever and asthenia.

Docetaxel and paclitaxel have been compared in an adjuvant study using one and three weekly schedules, respectively, in combination with doxorubicin/cyclophosphamide without significant differences between the schedules (45). However, in this study there was a non significant trend for a better outcome using the 3-weekly docetaxel schedule scheme and the weekly paclitaxel scheme (45).

The PACS 01 study, comparing three courses of $F_{500}E_{100}C_{500}$ followed by three courses of docetaxel at 100 mg/m^2, revealed an overall survival gain (p=0.017) compared with six courses of $F_{500}E_{100}C_{500}$ (46). The multivariate hazard ratio was 0.82 (95% confidence interval 0.69 – 0.99, p=0.34). The absolute survival gain was improved from 86.7% to 90.7% at five years for the

docetaxel arm. Patients younger than 65 years were included and the survival gain was mainly seen in patients older than 50 years. The hazard ratio was 0.81 for patients Er and/or Pgr positive cancers, the corresponding value for the receptor negative group was 0.79 (46). For patients receiving tamoxifen was the hazard ratio 0.89 (95% confidence interval 0.69 – 1.14), while it was 0.72 (95% confidence interval 0.55 – 0.93), for those without tamoxifen the chemotherapy induced amenorrhea was 72.4% in the FEC arm and 68.4% in the FEC-docetaxel arm respectively, (p=0.13). Febrile neutropenia was more common in the docetaxel arm (p=0.03), while the non-taxane arm had more cardiac side effects (p=0.03) (46).

The sequential design in the present study is also supported by the first analysis of the BIG-2 98 trial demonstrating a more favourable disease-free survival for patients receiving doxorubicin and docetaxel in sequence compared with concurrent use of these compounds (47).

Paclitaxel has shown to be active as a single drug, or in combination, in the treatment of metastatic breast cancer as first line therapy, as well as in patients with relapsed or refractory disease, even those who have failed to respond to prior anthracycline therapy (42, 48-50). The most common side effects are hypersensitivity reactions, neutropenia and peripheral neurotoxicity. The allergic reactions can be effectively controlled by standard pre-medication schedules containing corticosteroids.

In the adjuvant setting was paclitaxel studied in 3170 patients with node-positive breast cancer who had previously undergone mastectomy or segmental resection with auxiliary dissection CALGB 9344 (5). The patients were stratified according to number of involved lymph nodes (1-3, 4-9 or ≥10), with 46%, 42% and 12% of the patients respectively. The patients were randomly allocated to cyclophosphamide (600mg/m^2) and one of three doxorubicin doses, 60 mg/m^2, 75 mg/m^2 or 90 mg/m^2. Higher doses of doxorubicin (75 and 90 mg/m^2) were divided and delivered over 2 consecutive days to avoid dose intensity-related toxicity. Patients that received 90 mg/m^2 were given G-CSF and prophylactic ciprofloxacin. No benefit could be demonstrated in this study by escalating doxorubicin above 60 mg/m^2 (5). Following AC (doxorubicin, cyclophosphamide) therapy, patients received either paclitaxel or no further systemic therapy. Paclitaxel was given at a dose of 175mg/m^2 in a three-hour infusion every third week. Local irradiation was recommended to follow the completion of chemotherapy for patients that were subjected to breast conservative surgery. Patients with receptor positive disease were treated with daily tamoxifen 20 mg for 5 years after completion of chemotherapy.

The patients who received four courses of AC followed by four courses of paclitaxel had a statistically significant survival advantage by receiving 8 courses, versus four courses without paclitaxel. The absolute survival gain was 6% at seven years, 74% compared with 68% (5). In unplanned sub-group analyses, there was a DFS advantage for patients with ER negative tumours but not for those with ER positive tumours.

Paclitaxel after doxorubicin + cyclophosphamide has also been studied in the NSABP B28 study (51). Three thousand-sixty patients were randomised. This study also demonstrated a statistically significantly improved disease-free survival, an absolute improvement at 4% after 5 years. However, the overall survival was not statistically significantly improved. The authors claimed no interaction between the observed therapy effect and receptor status or the use of tamoxifen, however one can notice that tamoxifen for a long time has been given concurrently with chemotherapy by the NSABP-group.

2.7 Dose-dense therapy

Chemotherapy has traditionally been given at a maximum tolerated dose with intervals necessary for recovery from side effects i.e. mainly bone marrow recovery. This has been based on preclinical studies showing a relationship between the maximum tolerated dose and a higher percentage of cure rates. As a result, chemotherapy is given in most regimens every third or four weeks. However, this leads to an extended treatment-free period when tumour cells and endothelial cells necessary for the increased blood supply within the tumour also may re-grow. A more frequently delivery of chemotherapy reduces the non-exposure interval during which re-growth and neo-angiogenesis may occur.

The dose dense strategy with chemotherapy delivered every 2nd week instead of every 3rd week is a new and biologically highly interesting concept. The delivery of chemotherapy every 3rd or 4th week has been guided by this side effects rather than tumour biological considerations. The use of the cytokine G-CSF has facilitated the delivery of chemotherapy every 2nd week and surprisingly the data from this study did not demonstrate increased side effects by this procedure.

A recently presented large phase III study showed a benefit for patients treated with a dose-dense regimen with AC followed by paclitaxel given every second week, compared with the same treatment given every third week (6). The dose-dense therapy improved DFS (Risk Ratio 0.74, p=0.01) and OS (RR= 0.69, p=0.013) compared with therapy given every third week. The absolute survival gain was 2% at 3 years, 92% vs. 90%, in favour of the dose dense strategy. The relative reduction in hazard for breast cancer relapse was calculated to be 50% at 3 years and 52% at 4 years (52). Therapy was given with G-CSF support and in general well-tolerated, indeed severe neutropenia was less frequent in the dose-dense arms, probably in part due to the administration of G-CSF [2].

At ASCO 2004 with follow-up in San Antonio 2006 a dose intense and dose dense epirubicin-, paclitaxel- and cyclophosphamide-containing combination was demonstrated to be superior compared with AC followed by paclitaxel using conventional doses and scheduling (53, 54). At the median follow-up of 62 months 226 patients in the control arm had relapse compared with 182 in the dose dense and sequential arm (p=0.0016, hazard ratio 0.72, 95% confidence interval 0.59 – 0.87). Hundred and fourteen patients had died in the experimental arm versus 139 in the control arm (p=0.0285, hazard ratio 0.76, 95% confidence interval 0.59 – 0.97) (53). These data should be considered as a further support for the present study proposal.

In the present study proposal we have replaced paclitaxel with docetaxel while the latter taxane agent in indirect comparison and more recently in a direct comparison demonstrates efficacy superiority but also increased toxicity. The increased efficacy is the major motive to use docetaxel instead in the Citron regimen. The original Citron regime describes $A_{60}C_{600}$ followed by paclitaxel times 4. We have replaced doxorubicin to epirubicin 90 mg/m^2. It is accepted that epirubicin 90 mg/m^2 corresponds to doxorubicin 60 mg/m^2.

2.8 Data from the feasibility part of the SBG 2004-1 study

From November 2004 until May 2006 we randomised 124 patients in the feasibility study between the A (dtEC→dtT) arm in the present randomised phase III study, 2/3 of the patients were randomly allocated to either a tailored (A-arm), non-tailored (B-arm) dEC→dT, respectively, or 1/3 to a modified TAC-regimen (C-arm), given with G-CSF support (7).

In the phase II part of this protocol it was stated "The feasibility of treatment will be evaluated both according to side effects and dose intensity. The following criteria will serve as guidance". The prospectively decided criteria to evaluate were: delay in delivery of the courses, grade 3 and grade 4 infectious/febrile complications and requirement of hospitalisation.

This preliminary analysis is based on all given courses. 42, 41 and 39 patients started chemotherapy the A-, B- and C-arm, respectively. In total 305 courses were given in the A-arm (tailored and dose dense), 315 in the B-arm (non-tailored and dose dense) and 222 in the C-arm (3 weekly TAC with G-CSF). Cumulative mean doses in mg/m^2 per patient of delivered courses were: A-arm: Epirubicin 380,6 mg/m^2, cyclophosphamide 3118 mg/m^2 and docetaxel 322,5 mg/m^2. B-arm: Epirubicin 356,8 mg/m^2, cyclophosphamide 2395 mg/m^2 and docetaxel 276,3 mg/m^2. C-arm cyclophosphamide 2838 mg/m^2, doxorubicin 283 mg/m^2 and docetaxel 416,3 mg/m^2.

Accepting a 20% delay per course, i.e. treatment interval more than 16 days in arm B and more than 25 days in arm C. In arm A 16.7% had delays due to side effects, in the B-arm 19.0% delay due to side effects and in the C-arm 2.5% due to side effects. The courses were delayed due to other reasons in 3 patients, 3 patients and 1 patient in the A-, B- and C-arm respectively. In total 10 patients had delayed courses in the A-arm, 9 in the B-arm and 2 in the C-arm. Based on the pre-defined criteria that at least 90% of the patients shall receive the courses with less than 20% delay only the C-arm fulfilled this criterion. However, if calculated as events in relation to the total number of possible events the percentage in the A-arm is 4.3%, in the B-arm 3.5%, in the C-arm 0.9%.

Regarding clinical grade 3 and 4 side effects we have only recorded one patient with recorded grade 4 toxicity; 1 patient is described with grad 4 fatigue in the A-arm. For laboratory grade 3 and 4 toxicities we recorded the following:

		Arm A	Arm B	Arm C
Hemoglobin,	grade 3:	1	0	1
WBC,	grade 3:	33	17	27
	grade 4:	24	4	24
	grade 3 and 4:	35	17	34
Neutrophils,	grade 3:	29	18	14
	grade 4:	30	5	34
	grade 3 and 4:	34	20	35
Platelets,	grade 3:	1	0	2
	grade 4:	0	0	1
	grade 3 and 4:	1	0	2
ALAT	grade 3:	0	0	1

No patients in none of the arms have experienced a grade 4 infectious complication, that was stipulated to be ≤2 out of 40 planned patients. With reference to this criterion all arms were feasible.

Febrile neutropenia with granulocytes <1, fever ≥38.5°C. Four patients in the A-arm, none in the B-arm and 3 in the C-arm had this side effect recorded. Grade 3 infection combined with granulocytes <1: 2 in the A-arm, no patient in the B-arm and 3 in the C-arm had this recorded grade 3 side effect. Four patients in the A-arm also had a grade 3 infection with normal granulocyte values, 2 also had this grade 3 side effect recorded in the B-arm.

In the feasibility protocol it was stated that the grade 3 infections/febrile neutropenia was to be recorded in 20% or less of the patients. Based on this criterion 23.8% of the patients in the A-arm experienced this side effect, 4.7% in the B-arm and 15% in the C-arm experienced this side effect. If the grade 3 infection with normal ANC would be excluded the values would be 15% in the A-arm, 0% in the B-arm and 15% in the C-arm. We also, retrospectively, calculated as events in relation to the number of possible events (courses) and the figures would be 3.8% in the A-arm, 0.6% in the B-arm and 2.8% in the C-arm.

Regarding the pre-defined criterion of 20% or less of the patients should require hospitalisation: in the A-arm 12 (28.6%) patients have been hospitalised in the A-arm, 12 (28.6%) in the B-arm, and 8 (20%) in the C-arm. In addition 2 (4.8%), in the A-arm, 3 (7.1%) in the B-arm and 1 (5%) in the C-arm were hospitalized due to other reasons. Based on these data none of the treatment arm would according to the pre-defined criterion be considered as feasible. However, retrospectively we calculated each event in relation to the total number of possible events (number of courses) accordingly we recorded 6% of events due to hospitalisation in the A-arm, 4.4% in the B-arm and 4.1% in the C-arm due to side effect. In addition 0.7% in the A-arm, 1.0% in the B-arm and 0.9% in the C-arm were hospitalised for other reasons.

An amendment 5.11 was introduced after 124 courses in the A- and B-arm due to that 4 grade 3 hand- foot- skin-reactions have been recorded in the A-arm and 2 in the B-arm during the docetaxel part. After the addition of 1 week of extra pause between the EC- and docetaxel part 496 courses were delivered with 1 reported grade 3 hand- foot- skin-reactions in the EC-part of the A-arm and 1 event in the docetaxel part of the A-arm.

It was stipulated in the feasibility part of the protocol that “The following criteria will serve as guidance”, relating to pre-stipulated delays of courses, infectious complication and hospitalisation. As described above these very strict criteria were mainly not met but based on number of events in relation to number of exposures the therapies in all arms can then be considered to be manageable. One can also note that despite that we gave the TAC-arm with G-CSF it could according to our very likely to strict criteria not be considered to be feasible, despite that this was given with G-CSF in our feasibility part of the study. One should observe that the TAC-regimen is approved without G-CSF with a 25% risk of neutropenic complications. The fatigue item is very marked with total of 18 grade 3 reports in the A-arm, thereof 13 during the docetaxel part. The protocol has accordingly been modified, recommended that patients with grade 2 or more should consider not to be escalated to higher doses even if the other parameters would allow this escalation.

The first 93 patients received lenograstim (Granocyte®) while the remaining patients received pegfilgrastim (Neulasta®). Preliminary data demonstrated higher mean white cell values in all three arms on day 8 for pegfilgrastim compared with lenograstim. The opposite finding was noticed day 11/12. At day 1, corresponding to day 15 in the previous cycle, patients on pegfilgrastim tended to have higher mean values, especially in the non-tailored B-arm.

Based on this preliminary analysis of the feasibility part of the study we consider the dose dense and tailored EC→T arm manageable and to be the experimental arm in this randomised and prospective phase III study. We have replaced the previous TAC control arm with a $FE_{100}C$→T arm from the PACS 01 study (46). This arm should be considered as a well established standard arm which much more closely resembles the experimental arm in the present phase III study.

2.9. Motives for the present design of this phase III study

Three adjuvant chemotherapy studies have demonstrated a survival gain by the addition of a taxane, in two further studies a dose dense and sequential design was used (5-8, 53). In addition the first analysis of the BIG-2 98 trial demonstrated a more favourable disease-free survival outcome for patients receiving doxorubicin and docetaxel in sequence (47).
We have previously in the feasibility part of this study demonstrated that the use of dose dense and tailored EC followed by dose dense and tailored docetaxel is manageable, if an extra week of intermission is introduced between the EC- and docetaxel part. We have as well demonstrated an even better feasibility with fixed dose EC followed by fixed dose docetaxel using a dose dense strategy, with an extra week of intermission between the EC- and docetaxel part. This regimen is very similar to the regimen published by Citron demonstrating a survival gain by the dose dense strategy using the drugs doxorubicin, cyclophosphamide and paclitaxel. We initially planned to use the fixed dose EC→T regimen as the control and comparator arm to the tailored regimen. However, there have been questions regarding the acceptance of the EC→T regimen as an accepted control arm despite that it is very similar to the Citron regimen. We have therefore decided to use the recently published $FE_{100}C$→T (46) as control, which also more closely resembles our experimental arm compared with the TAC-arm.
The present experimental arm, tailored and dose dense EC→T, should be seen as a concept using as many as possible strategies of so far reported gains by adjuvant chemotherapy; polychemotherapy in sequence, a dose dense strategy, the regimen should have a duration which should be within the window of so far described maximal effect and the aim is to provide each individual an optimally adapted dose.
We therefore propose that the PACS 01 regimen will be used as a control arm, (46), instead of the TAC-arm due to a likely similar improvement in outcome but with less neutropenic side effect and therefore G-CSF is not mandatory in the FEC/docetaxel part of the PACS 01 study. This is also a fixed dose regimen without tailoring delivered on a three weekly basis. The total dose of docetaxel if given without dose modification is 300 mg/m^2 and the total dose of docetaxel in the tailored arm if no dose escalation is performed is also 300 mg/m^2. The duration of both the tailored EC followed by tailored docetaxel is 15 weeks which is also the duration for the PACS 01 regimen, three courses of FEC100 followed by three courses of docetaxel at 100 mg/m^2.
With the present study design our aim is to investigate whether tailoring and dose densification is superior to a fixed dose strategy without dose intensification.

2.10. G-CSF (Granulocyte colony stimulating factor)

G-CSF has previously in several studies been demonstrated to reduce the depth and duration of granulocytopenia and in some studies been demonstrated to reduce the risk of granulocytopenic fever and infections. G-CSF is mandatory for the use of dose-dense regimens. Previous randomised and comparative studies between G-CSF and pegfilgrastim have demonstrated similar effects by these two compounds (55-58). In the ASCO presentation from 2005 pegfilgrastim was demonstrated to statistically significantly reduce the risk for febrile neutropenia in patients receiving the TAC-regimen as part of the GEPARTRIO-study (59). The risk of febrile neutropenia was with G-CSF 17.1%, for pegfilgrastim 6.4% and pegfilgrastim combined with ciprofloxacin 4.3% ($p<0.001$ A versus B versus C) (59). These data indicate that pegfilgrastim is potentially a superior product compared with filgrastim. In the present phase III study, however, patients can be treated by either pegylated or non-pegylated G-CSF.

3 OBJECTIVES

3.1 Phase III

3.1.1 Primary objective

Compare breast cancer recurrence-free survival (BCRFS) (local-, regional-, distant breast cancer relapse or death due to breast cancer), in the tailored therapy arm (dtEC x 4→dtT x 4, A-arm) compared with the fixed dosed arm (FEC x 3→T x 3, B-arm).

3.1.2 Secondary objectives

1. Compare distant disease free survival (DDFS) (distant metastases or death due to breast cancer)
2. Compare event-free survival (breast cancer relapse, contra-lateral breast cancer, other malignancies, or any cause of death.
3. Compare overall survival (OS)
4. Health-related quality of life and toxicity analyses according to CTC
5. Outcome in relation to tumour biological factors and polymorphism patterns
    a. RFS in relation to the Sorlie classes using immunohistochemical markers and/or gene expression profiling comparing A vs B arm
    b. RFS with receptor positive disease (analyzed in the local laboratories as described in the CRFs and also analyzed as continuous variables) in the comparison between the A- and B-arms.
    c. RFS with high and low proliferation, respectively, (analyzed in the local laboratories as described in the CRFs and also analyzed as a continuous variable, or centrally analyzed), in the comparison between the A- and B-arms.
    d. RFS in relation to HER-2/neu status (analyzed in the local laboratories as described in the CRFs) in the primary cancers in the comparison between the A- and B-arms and anlyzed whether trastuzumab was given in sequence or concurrently.
    e. RFS analyzed in relation to other molecular markers (e.g. gene expression profiling/ sequencing) in the primary cancers and SNPs signatures in normal DNA (related to toxicities for EC/FEC and docetaxel components, respectively, and given dose levels and outcome in relation to these factors and in relation QoL) to outcome per arm
    f. RFS analyzed in relation to tumour associated lymphocytes and Y-box binding protein in the comparison between the A- and B-arms

Tumour tissue will be obtained and stored for studies of prognostication and therapy prediction.

3.2. Additional aims/ Biological markers

One important goal of the study is to evaluate gene- and protein based factors as well as other biological markers, which can be used for group and individual prognostication and therapy prediction. Therefore it is highly important that fresh frozen and paraffin embedded tumour material is saved for future analyses within the framework of this study. Via a separate consent, a separate tumour paraffin block and fresh frozen a part of the primary tumour together with normal cells (white blood cells) should be saved and stored from each patient for studies of prognostic, therapy predictive markers and if possible markers related to toxicity profiles. The patients will sign a separate consent for this part. It is thus strongly recommended that a fresh tumour biopsy, as large as possible, (at least 5-10 mm^3) divided into smaller parts (4 to 5 parts) to facilitate optimal freezing (to prevent degradation) should be frozen in liquid nitrogen. If liquid is not available, immediate

freezing on dry ice or immediate transport to a -70C° or -80C° freezer may be used. The coded and frozen tumour material must be stored in a -70C° or -80C°, ideally equipped with safety and alarm systems. DNA, cDNA (RNA as source) and protein will be analysed from the tumour tissues, with the most optimal techniques e.g.; PCR based studies, micro arrays, sequencing, proteomics using best available techniques and immunohistochemistry, aiming at identifying prognostic and predictive factors.
For white blood cells: Two 5 ml EDTA test tubes are taken with peripheral blood, at time of obtaining the standard laboratory test before starting chemotherapy. One tube is centrifuged; serum/EDTA plasma and blood cells are stored in minus -70°C. One tube is frozen in -70°C. Genomic DNA will be retrieved for potential single nucleotide polymorphism studies being of potential importance for studies of side-effects, therapy prediction and outcome. The samples will be stored at each centre and collected by the study monitor for storage at CCK, Radiumhemmet, Karolinska University Hospital. Material not used will be returned or destroyed according the Swedish Biobank regulations.

Biological sample collection is not mandatory for the Austrian sites. In case a site decides to collect and forward biological samples to Karolinska University Hospital, described procedure for separate patient consent and sample storage will be followed and samples will be delivered via special courier services.

In Germany a tumour paraffin block should be collected from each patient and sent to the central Pathology (Pathologisches Institiut der Charité/Berlin) for further analysis. For evaluation of predictive markers from biological samples peripheral blood (in total about 20 ml) should be taken from each patient. For isolation of plasma 5ml blood will be sampled in an EDTA test tube and inverted for several times (5x). After centrifugation (13000, 10-15 min), plasma can be collected from the supernatant. The plasma should be aliquoted into 3x2 ml test tubes and stored at -20°C to -80°C. For white blood cells 5 ml blood will be sampled in an EDTA test tube, inverted for several times (5x) and directly frozen at -20°C. For sampling of serum a S-Monovette (9 ml) test tube should be used. After coagulation (30 min. RT) and centrifugation (1500g/15 min) the serum is dispersed to 5x2ml test tubes and is also stored at -20°C to -80°C. Material for blood sampling and aliquotation will be made available in the laboratory kit that can be ordered at the GBG Forschungs GmbH (TraFo@germanbreastgroup.de). All frozen biological samples will be collected and transported to the GBG central Cryobank under the auspices of the GBG Forschungs GmbH. All biomaterials will be hold available for translational projects of the PANTHER study.

4 STUDY DESIGN

This is an adjuvant, open, prospective and randomised study. The results from our feasibility study together with other data are the basis for this randomized phase III study.

Table 1 Treatment arms

Dose dense and tailored therapy, every second week<br>One week extra between EC and T	A	dtEC x 4 → dtT x 4
Non-tailored every third week	B	FEC x 3→T x 3

4.1. Arm A: Two weekly dtEC→dtT (tailored and dose escalated dtEC x 4 + tailored and dose-escalated docetaxel x 4)

Dose escalated E $_{38\text{-}120\ mg/m2}$ C $_{450\text{-}1200\ mg/m2}$ will be given intravenously for 4 courses with G-CSF support. Courses should be given with a biweekly interval followed by 4 courses docetaxel 75-100 mg/m^2 with G-CSF support. The first course will be started at EC step 1, epirubicin 90 mg/m^2 and cyclophosphamide 600 mg/m^2 (Table 6). After completion of the **EC-part docetaxel is to be started the first time after three weeks for the fifth course**, followed by repeated two weekly courses for the remaining three courses. Total white blood cells (WBC), neutrophils and platelet nadir values at day 1, 8, and 11/12 will be measured at each course. Transaminases, alkaline phosphatase (ALP) and bilirubin will be measured day 1 each course. Dose modifications will be performed at each course based on the haematological toxicity (Table 4). If toxicity measured by CTC-NCI criteria are grade 2 or less (except haematological toxicity) it will be possible to escalate docetaxel to 100 mg /m^2. For dose modifications see Table 6.

4.2. Arm B: Three weekly FEC→T (FEC x 3 followed by docetaxel x 3)

Three courses of $F_{500}E_{100}C_{500}$, given with a 3-week interval, will be followed by three courses of docetaxel 100 mg/m^2 given with a 3-week interval. Neutrophil and platelet nadir values at day 1, 8, 11/12 and 14/15 will be measured during each cycle. Transaminases, ALP and bilirubin day 1 of each course.

5 STATISTICAL CONSIDERATIONS

5.1 Phase III

The primary objective of the phase III study is to compare breast cancer relapse-free survival (BCRFS) between the dtEC→dtT and FEC→T.

5.2.1 Endpoints

The primary endpoint is breast cancer recurrence free survival (BCRFS) defined as time from randomization to the first of the events; local-, regional- or distant breast cancer recurrence or death due to breast cancer or last date of follow-up if no event has occurred.

Secondary endpoints are:

- Distant disease free survival (DDFS) defined as time from randomization to the first of distant metastases or death due to breast cancer,
- Event-free survival (EFS) defined as time from randomization to the first of the events breast cancer recurrence (any type), contra-lateral breast cancer, other malignancy or any cause of death.
- Overall survival (OS), defined as time from randomization to any death.
- Health related quality of life and toxicity analyses according to CTC.
- Outcome in relation to tumour biological factors and polymorphism patterns

Dose intensity and side effects are tertiary endpoints.

The patients will be allocated to respective treatment arm using block randomization with varying block size. The randomization will be stratified per site and by receptor status. A patient is receptor negative if both estrogen- and progesterone receptors are negative; otherwise she is receptor positive.

5.2.2 Choice of treatment arms and number of patients

In the present study it will take about 6 years, or more, from study design until analysis and presentation, and a high-rank aim is to design a study that answers research questions that are of interest at that time. Ideally there is a standard control arm and an experimental arm in a clinical study, but due to the rapid development it is impossible to know what will be considered standard treatment in 6 years time and hence both the treatment arms are experimental. Our aim is that the present phase III design will fulfil this.

The original dimensioning aims at detecting a 10% absolute increase in 5-year BCRFS from 0.70 to 0.80 with 80% power, and for this 150 events are necessary. See Section 5.2.2. "Choice of treatment arms and number of patients" in the protocol from December 2006. From a clinical point of view it is important to be able to detect a smaller difference, and then more events are necessary and thus more patients should be recruited.

Assume that 13 patients per month are recruited by SBG (Sweden) from January 2007 until January 2010 (468 patients), and that 13 patients per month are recruited by ABCSG (Austria) from January 2008 until January 2010 (456 patients) and that GBG (Germany) will recruit 40 patients per month from October 2008 until January 2010 (600 patients). The Finnish inclusion is to be included either in the Austrian, German or Swedish cohort, in total 1524 patients. All patients will be followed two extra years, i.e. until January 2012. Then about 225 events will have occurred (based on Monte Carlo simulations assuming exponential survival distributions with average 5-year BCRFS of 0.75, as above), and this gives 80% power to detect an absolute difference in 5-year BCRFS of 8.0%, from 0.710 to 0.790 (corresponding to a hazard ratio of 1.46). See page 170 in ref. 60 (Piantodosi S, Clinical trials, a methodological perspective. Wiley, New York 1997).

There is a large uncertainty concerning the average 5-year BCRFS and thus the average event rate. In the dimensioning, 5-year BCRFS is assumed to be in average 0.75, and this should be checked with a blinded interim analysis after including about 80% of the planned number of patients. This analysis will make it possible to predict how many events will have occurred at the planned closing date, and thus to adjust number of patients per participating group and/or extra follow-up.

In August 2010 the planned interim analysis of the average event-rate was performed, based on about 1200 patients included until May 2010. The observed event-rate was somewhat lower than assumed in the dimensioning of the study. Thus it was decided to increase the number of patients to n=2000, and this number will be reached in mid 2011. According to the interim analysis the chance is then good to reach the goal of 225 primary events in December 2012.

If data from upcoming studies like the SBG 2000-1 study will reveal a less marked difference between tailored doses versus fixed doses, the patient number need also to be re-examined by the independent Data Monitoring and Safety Committee together with the study statistician.

5.3 Analysis sets

The main analyses will be done according to the intention to treat principle and thus all randomised patients will be included in this analysis. Only patients clearly known to be non-eligible at the time of randomization will be excluded.

The data will also be analysed per protocol. For instance, patients not treated according to randomization and patients with serious hypersensitivity against any cytotoxic treatment will be excluded.

5.4 Statistical analyses

Demographic data, and clinical, pathological and other prognostic variables as well as toxicity, safety and treatment parameters such as dose intensity will be presented by treatment arm in descriptive tables.
The time to event endpoints will be illustrated by treatment arm using Kaplan-Meier curves and cumulative incidence curves in case of competing risks. The primary treatment comparisons will be done using log-rank tests stratified in the same way as the randomization. Using Cox regression, analyses adjusted for important prognostic factors will also be done. Hazard ratios with 95% confidence intervals calculated with the score statistic will be provided.

Various biological factors will also be studied (*e.g. PCR based studies, micro arrays, sequencing, proteomics using best available techniques and immunohistochemistry)*, either directly for all patients or later for all patients or a sub sample. The collection of the biological samples are prospective in a randomized study, results derived should be interpreted accordingly. The co-variation between these factors and histopathological factors will be analysed. The prognostic/predictive effect of the biological factors on BCRFS and overall survival will be analysed by means of Cox regression, conditional logistic regression or other more specialized statistical techniques. The analyses should also, when possible, take the causal order between the factors into account.

Adverse events of at least CTC grade 3 will be presented by event status and time-to-event by a Kaplan-Meier curve with a logrank test and hazard ratio with 95% confidence interval assessed with the score statistic in a Cox regression analysis.

6 PATIENT ELIGIBILITY

Patients should be offered this chemotherapy study based on previous results using taxane based chemotherapy, dose dense chemotherapy, tailored dosage and therapy recommendations described in international and national guidelines.

6.1 Inclusion criteria

1: Histological proven invasive primary breast cancer, with at least 5 (recommended 10) removed axillary lymph nodes **OR** negative sentinel node biopsy performed for the node negative cohort. Interval between definitive surgery that includes axillary lymph node dissection and registration must be less than 60 days. Paraffin block from the primary tumour must be retained (not mandatory for Austrian sites).
Frozen tumour tissue is strongly recommended to be stored.

2. Receptor negative or positive tumours with 1 or more positive axillary lymph nodes (more than 0.2 mm) **OR** axillary node negative breast cancers if the primary tumour is larger than 20 mm and receptor negative (Er and Pgr with no receptor content) and being Elston grade III.
In Germany high risk node negative breast cancer patients are not eligible until labelling for docetaxel includes node-negative disease.
3. A primary breast cancer patient being 35 years or younger considered suitable for adjuvant chemotherapy (may be receptor negative or positive, HER-2/neu negative or positive, with or without axillary lymph node metastases).
4. Macroscopically and microscopically free margins after radical surgery (no cancer cells at borders of resection).
5. No proven distant metastases (negative chest/pulmonary X-ray, bone scintigram (when clinical signs of skeletal metastases or elevated ALP) supplemented with normal conventional X-ray of hot spots, normal liver function test and haematological function tests; when abnormal values, CT or ultrasound of the liver, patient can be included if no metastases are demonstrated.
6. Female age 18-65.
7. Ambulant patients (ECOG 1 or less).
8. No major cardiovascular morbidity NYHA I or II. (Appendix 3).
9. Written informed consent according to the local ethics committee requirements.
10 Patients of childbearing potential should have a negative pregnancy test within seven days of registration. (In Austria, pregnancy tests have to be repeated monthly during the treatment phase).

6.2 Exclusion criteria

1. Previous neo-adjuvant treatment.
2. Non-radical surgery (histopathological positive margins).
3. Proven distant metastases.
4. Pregnancy or lactation.
5. Other serious medical condition.
6. Previous or concurrent malignancies at other sites, except basal cell carcinoma and/or squamous cell carcinoma in situ of the skin or cervix. Patients with previous breast cancer (invasive and/or ductal carcinoma in situ) in the other breast without loco-regional (large lung volumes) radiotherapy, without objective findings for relapse, with > 5 years since diagnosis can be included.
7. Abnormal laboratory values precluding the possibility to safely deliver the used cytotoxic agents in the study.
8. Hypersensitivity to drugs formulated in polysorbate 80.
9. Peripheral neuropathy grade ≥ 2.

7 PATIENT INCLUSION AND RANDOMIZATION

7.1. Patient information and consent

Patients who fulfil the inclusion criteria will receive written and oral information, and approve participation in the study by signing the written patient information. Patients who also approve participation in the biological marker study will receive written and oral information and sign the written patient information. The consent forms, which contain the patient's signature and date, signature of informer and date, must be completed and retained, as part of the investigator file. The patient should receive copies of the patient information and the signed consent forms. For Austria it is sufficient to retain all original duly signed informed consents in the investigator files and to

provide one copy to the patients. As a part of the required content of the informed consent, the patient must be informed that authorised monitors and/or health authorities may review relevant parts of the patient record and medical notes. No study procedures may start before obtaining signature.

7.2 Randomization

Sweden: The inclusion form will be faxed to the randomization office located at the Clinical Research Unit, Karolinska University Hospital, fax number +46-8-306989. A copy of the inclusion form, together with treatment allocation, will be returned by fax. The form is generally returned within one hour during working hours, or at the latest the next working day. The patients will be allocated using block randomization with varying block size.

Austria: The Randomization will be executed with a web-based system (https://www.muw.ac.at/randomizer/web/login.php) via direct entry. The confirmation of randomization will be automatically generated by the system and will be sent back to the specified recipient via e-mail.

Germany: The registration form and baseline CRF pages will be faxed to the GBG Forschungs GmbH, Germany, fax number +49-7480-440, and the randomization result will be faxed back to the participating centres within one working day.

Finland: The inclusion form will be faxed to the randomization office located at the Clinical Research Unit, Karolinska University Hospital, Stockholm Sweden, fax number +46-8-306989. A copy of the inclusion form, together with treatment allocation, will be returned by fax

8 THERAPY AND STANDARD PROCEDURES BEFORE RANDOMIZATION

8.1 Baseline investigations

Routine blood tests including neutrophils will be done 2 weeks prior randomization. Chest X-ray or chest-CT must be performed within 6 weeks prior randomisation. ECG, ECHO or MUGA scan shall be performed within 4 weeks prior to randomization. No predefined cut-off levels are given, but the laboratory values must be adequate for considering therapy within the study. If clinical signs of skeletal metastases or elevated ALP occur, a bone scintigram must be performed. If hot spots on bone scintigram can be identified, x-ray or MRI shall be done; if abnormal liver enzymes, ultra sound or CT scan of the liver/abdomen shall be done within 4 weeks prior to randomization. Pregnancy test shall be done within 1 week prior to randomization in women of childbearing age. In Austria, pregnancy tests will be performed monthly during treatment.

8.2 Surgical treatment

All patients must be radically operated with mastectomy or segmental resection and axillary exploration, with recommended 10 removed and histopathological investigated lymph nodes. **An absolute minimum for inclusion in the study is 5 examined axillary lymph nodes**. A patient with performed sentinel node biopsy or axillary dissection, being negative upon histopathological examination, is allowed for inclusion in the node negative cohort.

8.3 Histopathology and immunohistochemistry

The histopathological diagnosis of an invasive breast cancer must be established. The tumour must be graded using a well-established system, e.g. Elston-Ellis.
Oestrogen and progesterone receptor status must be determined with an established technique. The cut-off between receptor negative (hormone unresponsive) and receptor positive (hormone responsive) should be performed according to local guidelines.
It is recommended that the HER-2/neu status in the primary tumours should be determined with immunohistochemistry, supplemented with FISH verification of immunohistochemical grade 2+ or 3+ positivity (in Germany only for grade 2+).

9 DOSAGE AND ADMINISTRATION OF THERAPY

9.1 Arm A: biweekly dtEC→dtT - Dose escalated and tailored EC x 4 (three weeks break) followed by biweekly dose-escalated and tailored docetaxel x 4

Four courses of dEC are given at day 1, repeated day 15. The first course is given at step 1 (E 90 mg/m^2 C 600 mg/m^2) (Table 3). Control of WBC, neutrophils and platelets counts on day 1, 8, and 11/12 is done during each course, and dose-modifications are done according to haematological toxicity (Table 4). In case one blood sample has not been taken, the dose should be considered not to be escalated, but repeated the at same step as in the previous cycle unless the values deteriorate making a delay or dose reduction necessary.

Table 3. Treatment arm A – dtEC - dosages (mg/m^2)

	Epi	Cyclo	Mesna (mg/m^2)
Step-3	38	450	
Step-2	60	600	
Step-1	75	600	
**(Starting dose level= Step 1)**			
Step 1	90	600	
Step 2	105	900	
Step 3	120	1200	240, hours 0, 4, 8 iv∗

All drugs are given day 1, length of cycle 2 weeks. Acceptable time window to give treatment is ±1 day. Total treatment period four courses.
Course 1 is given according to step 1, courses 2-4 according to table 4. ∗Note that mesna also can be given orally at hours 2 and 6 (480 mg/m^2) after cyclophosphamide infusion.
Dosing should always be based on the actual BSA calculation at each course (NB no cut-off above 2.0 m^2 BSA). It is recommended to use the method after Dubois for BSA-calculation.
Chemotherapy preparation should be performed according to institutional procedures.

Mesna is given if cyclophosphamide doses are higher than 1g/m^2. Mesna should be given intravenously at a dose of 20% (240 mg/m^2) of the cyclophosphamide dose at 0 h, 4h, and 8 h. Following iv administration of mesna at 0h, it can be given orally at doses of 40% of the cyclophosphamide dose at 2h and 6h after start of cyclophosphamide infusion.

Ciprofloxacin should be given 0.5 g x 2 days 5 to 12 after each **EC** course as prophylactic antibiotic treatment. Neutropenic fever will be treated according to local practice. Ciprofloxacin should not be given during the docetaxel part of the study but will be used at secondary prophylaxis. This is a change compared with the feasibility study.

Anti-emetics: Serotonin (5HT3) receptor antagonist therapy combined with repeated high doses of beta-/dexametasone must be used to prevent acute vomiting and delayed emesis, to be used when need together with other anti-emetic drugs, according to local practice.

9.1.1 G-CSF

G-CSF can be used as filgrastim (using approved dosages) given subcutaneously day 4-11 during the EC part, day 4-10 during the docetaxel part. If pegfilgrastim is used the starting day should be day 2, both during the EC and docetaxel part.

9.1.2 Dose-modifications - dtEC

Course 2-4 are given as follows:

NADIR:

- **Leukocytes (WBC) >1.0 (nadir), platelets >50 (nadir) and previous (day 8 or 11/12) /later (day 8 or 11/12) WBC >1.5 and platelets >75 and at the start of the following course (day 14/15) WBC ≥3.0 and platelets ≥100. Dose escalation will be done with one step per course according to table 3.**
- If prolonged and/or deeper NADIR values according to above, but normal values at day 14/15 (WBC ≥3.0 and platelets ≥100), next course should be given unchanged.
- Day 14/15: If WBC 2.0 – 2.9 and/or platelets 75 – 99 give the next cycle as scheduled but reduce the dose with two steps. If the patient tolerates this, escalate the following dose according to the scheme again.
- Day 14/15: If WBC <2.0 and or platelets <75 delay therapy one week. If normal values the next week, give the course reduced by 2 dose steps compared with the previous course, however not lower than step 1. If the patient tolerates this, escalate according to the scheme again.
- Day 14/15: On previous step 1 or step -1 with the following values: WBC 2.0 – 2.9 and/or platelets 75 – 99: Give 50% of EC step -2 (= step -3).
- Day 14/15: If (neutropenic) fever during the latest course, (<38.5 C°), also if no haematological toxicity occur reduce the next course with one dose-step.
- Day 14/15: If uncomplicated neutropenic-fever CTC grade 3 (≥38.5 C°) reduce the next course with one dose-step. If more complicated neutropenic-fever CTC grade 3 or 4 (Life threatening consequencies, e.g. septic shock, hypotension, acidosis, necrosis) discuss with study co-ordinator and reduce with two dose-steps.

Table 4. Dose-modification schedule - arm A – dtEC→T (dtEC arm for the first four courses)

Nadir day: 8 or 11/12			Day 8 or 11/12 (except nadir value)			Day 14/15			Action
**WBC**	**Platelets**		**WBC**	**Platelets**		**WBC**		**Platelets**	
> 1.0	> 50	And	≥ 1.5	≥ 75	And	≥ 3.0	and	≥ 100	Dose escalation. Continue to next step
≤ 1.0	≤ 50	and/or	< 1.5	< 75	and	≥ 3.0	and	≥ 100	Repeat same step for next cycle
Any	≤ 15	and/or	Any	Any	and	≥ 3.0	and	≥ 100	Reduce 1 step
> 1.0	> 50	and	≥1.5	≥75	and	2.5-2.9	and	≥100	Repeat same step for next cycle
Any	Any	and	Any	Any	and	2.0-2.9	and/ or	75-99	Reduce 2 steps; if tolerated then escalate according to schedule
Any	Any	and	Any	Any	and	< 2.0	and/ or	< 75	Delay cycle by 1 week; reduce dose by 2 steps when normal values return

In addition; Diarrhoea grade ≥3 or stomatitis grade ≥3, reduce the dose the next course, discuss with study co-ordinator. For neutropenic fever, please see previous page, 9.1.2.

9.1.3 Dose dense and tailored docetaxel x 4 (after dtEC x 4)

After completion of the EC-part in the A-arm docetaxel is to be started the first time after three weeks for the fifth course, followed by repeated two weekly courses for the remaining three courses. The reason for the insertion of an extra week between course four and five is due to experiences in a French study and partly supported by our own experiences in the previous feasibility part of the SBG 2004-1 study.

All patients in the docetaxel part are to be started at a dose at 75 mg/m^2. The following courses are to be delivered every second week, with G-CSF support, using tailoring based on haematological toxicity as outlined below. Acceptable time window to give treatment is ±1 day. Dosing should always be based on the actual BSA calculation at each course (NB no cut-off above 2.0 m^2 BSA). It is recommended to use the method after Dubois for BSA-calculation. Chemotherapy preparation procedure should be performed according to institutional procedures.

All patients in this arm should start at 75 mg/m^2: If patients have less than or equal to grade 2 toxicity (excluding alopecia and neurotoxicity), *dose-escalate to next level.* If this results in feasibility problems or higher than grade 2 toxicity, reduce the dose for the next course to 75 mg/m^2. For this group of patients, do not then re-escalate again.

Dose modification schedule of docetaxel

Table 5

**Dose level**	**Dose of docetaxel**
-1	60 mg/m^2
**0 (starting dose level)**	75 mg/m^2 >1.5 x 10^9granulocytes/l and WCB ≥3.0 x 10^9
1	85 mg/m^2
2	100 mg/m^2

G-CSF: Filgrastim should be started on day 4 through day 10. Alternatively, pegfilgrastim should be administrated on day 2 after chemotherapy at the dose of 6 mg subcutaneously.

Ciprofloxacin should not be given as primary prophylaxis during the docetaxel part. This is a change compared with the feasibility part. However, ciprofloxacin should be used as 0.5 g x 2 daily days 5 to 12 as secondary prophylaxis after an eventual neutropenic/infectious complication.

Recommended premedication for docetaxel is
Betamethason 0.5 mg, 8 mg twice daily for three days –1, 0 and 1 (in Germany: Dexamethasone 20 mg i.v. before start of docetaxel infusion, 4 mg p.o. twice daily on day 2 and 3 and once daily on day 4 after chemotherapy).

Table 6. Dose-modification schedule - arm A –docetaxel for the last four courses

Nadir day: 8 or 11/12			Day 8 or 11/12 (except nadir value)			Day 14/15			Action
**WBC**	**Platelets**		**WBC**	**Platelets**		**WBC**		**Platelets**	
>1.0	>50	and	≥1.5	≥75	and	≥3.0	and	≥100	Dose escalation. Continue to next step
≤1.0	≤50	and/or	<1.5	<75	and	≥3.0	and	≥100	Repeat same step for next cycle
Any	≤15	and/or	Any	Any	and	≥ 3.0	and	≥ 100	Reduce 1 step
>1.0	>50	and	≥1.5	≥75	and	2.5-2.9	and	≥100	Repeat same step for next cycle
Any	Any	and	Any	Any	and	2.0-2.9	and/or	75-99	Reduce 2 steps; if tolerated then escalate according to schedule
Any	Any	and	Any	Any	and	< 2.0	and/or	<75	Delay cycle by 1 week; reduce dose by 2 steps when normal values return

Patients with fatigue grade 2 or more consider not escalating further, re-evaluation at each course.

In addition; **Diarrhoea grade ≥3 or stomatitis grade ≥3, reduce the dose** the next course, discuss with study co-ordinator. **Neutropenic fever: Discuss with study co-ordinator.** Emesis and vomiting higher than grade 2 should be treated with best anti-emetic therapy including high doses of corticosteroids 8-20 mg betametasone iv and 5HT^3-blocking agents.

Table 7

ASAT/ALAT values	Alkaline phosphates values	Dose Modification
$\leq$ 1.5 x UNL	$\leq$ 5 x UNL	No dose modification
> 1.5 x UNL to $\leq$ 2.5 x UNL	$\leq$ 2.5 x UNL	No dose modification
> 2.5 x UNL to $\leq$ 5 x UNL	< 2.5 x UNL	Reduce dose of docetaxel one dose level
> 1.5 x UNL to < 5 x UNL	> 2.5 x UNL to < 5 x UNL	Reduce dose of docetaxel one dose level
> 5 x UNL	> 5 x UNL	Dose delay by a maximum of 2 weeks. If then no recovery to the above figures, patient should go off study

UNL= Upper normal limit.
If a patient experiences several toxicities and there are conflicting recommendations, please follow the most conservative dose adjustment recommended and discuss with study co-ordinator.
Neurotoxicity NCI grades 1 or 2 - give the next course according to haematological toxicity.
Neurotoxicity NCI grade 3 - reduce next course two dose steps, regardless if haematological toxicity. Keep this dose at remaining courses.
Neurotoxicity NCI grade 4- stop further treatment with docetaxel.

9.2 Arm B: Three weekly fixed dosed FEC→T – $FE_{100}C$ x 3 followed by three weekly docetaxel x 3 (100 mg/m^2 without escalation)

Three courses of $F_{500}E_{100}C_{500}$ mg/m^2, given with a 3-week interval followed by three courses of T (docetaxel 100 mg/m^2) as a 1-hour infusion given with a 3-week interval. Dosing should always be based on the actual BSA calculation at each course (NB no cut-off above 2.0 m^2 BSA). It is recommended to use the method after Dubois for BSA-calculation. Chemotherapy preparation procedure should be performed according to institutional procedures.

Anti-emetics for the FEC part: Serotonin (5HT3) receptor antagonist therapy combined with repeated high doses of beta-/dexametasone must be used to prevent acute vomiting and delayed emesis, to be used when need together with other anti-emetic drugs, according to local practice.

Recommended premedication for docetaxel is
Betamethason 0.5 mg, 8 mg twice daily for three days –1, 0 and 1 (in Germany: Dexamethasone 20 mg i.v. before start of docetaxel infusion, 4 mg p.o. twice daily on day 2 and 3 and once daily on day 4 after chemotherapy).

9.2.1 Dose-modifications - FEC→T

Control of white blood cells, neutrophils and platelets counts on day 1, 8, 11/12 and 14/15 is performed during each course**.**

In the event of bone marrow suppression:
On day 21 (next course day 22), if the neutrophil count is ≤1.5 x 10^9/l or platelet count ≤100 x10^9/l, the treatment will be delayed for eight days and resumed with co-administration G-CSF (filgrastim or pegfilgrastim) in all the remaining cycles using standard dosage strategies. If for the next course this was not sufficient and the patient on day 21 has neutrophils ≤1.5 x 10^9/l or platelet count ≤100 x 10^9/l reduce epirubicin to 75 mg/m^2, remember to give the course with G-CSF. If that is not sufficient reduce 5-fluorouracil, epirubicin and cyclophosphamide with 25% the following course, remember to give this course also with G-CSF.

In arm B, the full docetaxel (100 mg/m^2) dose will always be given in the first cycle. At each course, docetaxel should be administered only when neutrophil count is >1.5 x 10^9/l.

On day 28, if the neutrophil or platelet count precludes treatment, the patient will be withdrawn from the study.

In the event of bone marrow aplasia with fever the following can serve as guidance:
Definition: fever ≥38°C in a period of bone marrow hypoplasia **(granulocytes < 0.5 x 10^9/l) requiring antibiotic therapy or lasting more than 24 h.**

- Consider hospital admission
- pre-antibiotic collection of specimens for bacteriology
- complete blood count with differential and blood culture should be performed every other day until recovery of ANC ≥ 0.5 or temperature < 38.1°C
- start of an antibiotic therapy if ANC< 0.5 X 109/L preferably according to an antibiogram
- The treatment will be continued at the same doses but with concomitant administration of filgrastim/pegfilgrastim in all the remaining cycles

If another episode of fever occurs despite filgrastim treatment, the doses of all the products must be decreased by 25% for all the remaining cycles. If this problem occurs in arm B before the docetaxel cycles, docetaxel must be initiated at the full dose without administration of filgrastim.

In the situation of low neutrophil count or low platelet counts during the docetaxel part, as not described in the text here dose reductions and delay of course is and/or administration of G-CSF **must be discussed with the study co-ordinator.**

Any other toxicity above grade 2 (excluding alopecia and neurotoxicity), shall result in dose reduction of 25% until recovery to grade 1 or less. Emesis and vomiting higher than grade 2 should be treated with best anti-emetic therapy including high doses of corticosteroids 8-20 mg betametasone iv) and $5HT^3$-blocking agents.

If neutropenic fever, (≥38.5°C), combined with other serious clinical condition during the latest course, grade 4 toxicity, e.g. septic chock, **discuss with the study co-ordinator** and reduce the doses with 50%.

Table 8. Dose modification schedule of docetaxel

Dose reduction	Dose of docetaxel
40%	60 mg/m^2
25%	75 mg/m^2
0 (starting dose level)	100 mg/m^2 (>1.5 x 10^9 granulocytes/l and platelets >100 x 10^9/l)

In the event that ASAT and or/ALAT alkaline phosphates levels are abnormal in the absence of relapse, dose modification are recommended as described in Table 9.

Table 9

ASAT/ALAT values	Alkaline phosphates values	Dose Modification
>1.5 x UNL to ≤2.5 x UNL	≤2.5 x UNL	No dose modification
>2.5 x UNL to ≤5 x UNL	≤2.5 x UNL	Dose reduction to 75 mg/m^2
>5 x UNL	>2.5 x UNL	Stop docetaxel treatment definitely

UNL= Upper normal limit.

If total bilirubin is above upper normal limit, docetaxel should also be discontinued.
If a patient experiences several toxicities and there are conflicting recommendations, please follow the most conservative dose adjustment recommended, and discuss with study co-ordinator.
Neurotoxicity NCI grades 1 or 2 - give the next course according to haematological toxicity.
Neurotoxicity NCI grade 3 - reduce next course with 25%, regardless if haematological toxicity. Keep this dose at remaining courses.
Neurotoxicity NCI grade 4- stop further treatment with docetaxel.

9.3Administration of cytotoxic drugs

The use of a subcutaneous venous access port is recommended. The used cytotoxic agents must be administered according to the schedules in the tables 10and 11.

9.4.1. dtEC→dtT (A arm)

Table 10 **EC**

Administration order	Drug	Administration time
1	Epirubicin	30-60 min. infusion
2	Cyclophosphamide	15-30 min. infusion
3	Mesna (for the 1200 mg/m^2 Cyclophosphamide dose)	10' infusion (240 mg/m^2) h 0, 4 and 8 *or* iv infusion h 0 and oral capsules at a dose of 480 mg/m^2 at h 2 and 6

Docetaxel

Administration order	Drug	Administration time
1	Docetaxel	1 hour infusion

9.4.2. FEC→T (B arm)

Table 11 FEC

Administration order	Drug	Administration time
1	5-fluorouracil	Bolus injection <5 minutes
2	Epirubicin	30-60 min. infusion
3	Cyclophosphamide	15-30 min. infusion

Docetaxel

Administration order	Drug	Administration time
1	Docetaxel	1 hour infusion

9.5 Auxiliary therapy

Optimal anti-emetic treatment according to local practice is recommended. Anti-emetic therapy shall also include $5HT^3$-blocking agents. Anti-emetic treatment with corticosteroids in adequate doses (8-20 mg beta-/dexametasone iv on treatment day, followed by oral beta-/dexametasone twice daily) is strongly recommended, but should not be used beyond day 5 of the chemotherapy cycle. In case of neutropenic fever; hospitalisation and appropriate antibiotic treatment according to local practice is done. Other anti-neoplastic treatment, than specified in this protocol, is not allowed during the trial.

9.6 Postoperative radiation

Additional anti tumour treatment may only start, at the earliest two weeks after last dosing of study drug or after recovery from any toxicity.

Postoperative radiotherapy should be given at a total dose of 45-50 Gy/1.8-2 Gy/day to the scar or remaining breast parenchyma *and* regional lymph nodes according to local guidelines. However, patients operated with breast conserving surgery must receive radiotherapy to the remaining breast parenchyma. Three-dimensional dose planning is strongly recommended, aiming at avoiding radiation doses to heart, larger vessels and lung tissue. For women younger than 40 (50) years operated with breast conserving surgery, the addition of a boost dose of 16 Gy is strongly recommended (2.0 Gy fractions/day or brachytherapy). Radiotherapy should be started as soon as possible after completion of all chemotherapy courses, however not later than 6 weeks from the last chemotherapy course.

9.7 Hormonal therapy

Only patients with verified hormone receptor positive disease would be recommended adjuvant hormonal therapy. The hormonal therapy ***must not be given concurrently with*** *chemotherapy*. The starting date should be recorded in the CRFs and patient records. Tamoxifen 20 mg/day for 5 years has for many years been standard adjuvant hormonal therapy for endocrine responsive disease. Based on recent data it is very reasonable that patients in the present study will be offered either an upfront aromatase inhibitor or tamoxifen for 2-3 years followed by an aromatase inhibitor, or potentially the reversed sequence if later demonstrated to be superior. Type and duration of adjuvant therapy as well as menstruatial status before start of chemotherapy and start of adjuvant endocrine therapy should be recorded in the CRFs.

Premenopausal patients who continue to menstruate after completion of adjuvant chemotherapy can be considered for LHRH analogue therapy, in particular younger than ≤ 35 years age. The menopausal status must be recorded in the CRF (using menstrual data, if uncertain please determine FSH) before start of chemotherapy and after completion (using menstrual data, if uncertain please determine FSH) of chemotherapy. The strategies for choice of adjuvant hormonal therapy should be predefined for each institution before start of the study.

9.8 Trastuzumab

Patients with a breast cancer with FISH verified amplification of HER-2 (or validated 3+ immunohistochemical overexpression) are recommended to receive adjuvant trastuzumab. This therapy should be started 4 to 8 weeks after completion of docetaxel chemotherapy, irrespective of the start of radiotherapy. Another alternative is to start trastuzumab therapy concurrently with docetaxel. This may increase the efficacy, but at the expense of increased cardiac toxicity (62-65). The discontinuation of trastuzumab therapy was 4.3% in the HERA while it was 15.6% in NSABP B-31. Each institution must predefine which trastuzumab strategy they aim to use, must be the same for both therapy arms for each institution. Trastuzumab should be given using the three weekly schedule for one year. If the ejection fraction is decreased with 10% or more or if the ejection fraction is below 50%, the therapy should be stopped or postponed until an improvement is recorded. Ejection fraction should be evaluated by ECHO- cardiography or MUGA scan before start of trastuzumab infusions, to be repeated at 6 months and at completion of trastuzumab therapy, and repeated in the follow up according to national guidelines – SPC-text or as described in the previous version of this study protocol, 3 and 5 years after completion of trastuzumab therapy.
If the PANTHER study is accepted as a feeder trial to the ALTTO study, participation of a patient in both studies is allowed. This is not applicable for patients in Sweden and Finland.

9.9 Other anti-cancer agents

Other drugs in this category are not allowed. However, bisphosphonates can be used if it is part of a predefined institutional policy.

9.10 Assessment of health related quality of life

Health related quality of life will be assessed, at least in all Swedish patients entered in the study. Base line assessment will be performed after informed consent but before randomisation. The EORTC QLC-C30 is the instrument which will be used. The detail for the quality of life evaluation is described in appendix 5. The responsible person for this part will be Professor Yvonne Brandberg at Radiumhemmet, Karolinska Institutet, 171 76 Stockholm, Sweden.

10. PHARMACOLOGICAL INFORMATION

10.1. Pharmacological information

10.1.1. Epirubicin

Haematological
The dose limiting side effect is myelosuppression. Leukopenia is more frequent than thrombocytopenia.

Fever and Infection
Fever is commonly reported and at times associated with severe neutropenia. Febrile neutropenia is reported in 6.6% of patients treated with epirubicin one day every third week in the adjuvant setting. The most frequently reported infectious complications included urinary tract infections, upper respiratory tract infections and sepsis.

Cardiovascular
Two types of cardiotoxicity may occur. The acute cardio toxicity is almost always reversible, and consists of arrhythmias and/or ECG changes. The second is a chronic cumulative and dose dependent congestive heart failure (CHF) with disruption of myofibrils and vacuolisation of myocardial cells. The clinical manifestation of CHF is seen at doses over 1000mg/m^2. Previous cardiac irradiation, anthracycline or anthracene therapy lowers the cumulative dose of epirubicin that can be tolerated before the onset of CHF.

Local necrosis with extravasation
Leakage of small amounts of drug into surrounding tissue may result in pain, necrosis and ulceration of the area. The affected area in general heals slowly. There is at present no specific therapy to prevent such necrosis. The current recommendation management consists of application of ice packs to the area with elevation of the extremity. If pain persist or increase, inflammation and oedema develops, early consultation with a plastic surgeon shall be taken. **It is recommended that all patients included in the present study have a permanent venous access before chemotherapy is started.**

Gastrointestinal
Nausea and vomiting may occur, but to a lesser degree than observed with doxorubicin. Sufficient anti-emetic therapy including corticosteroids may alleviate much of these symptoms.

Secondary leukaemias
Patients earlier treated with epirubicin in combination with other antineoplastic agent have a risk of developing a secondary acute myeloid leukaemia. This risk is estimated as below 1% for patients earlier subjected to adjuvant chemotherapy for breast cancer.

Mucositis
Mucositis may occur, above all at higher doses.

10.1.2 Cyclophosphamide

Urotoxicity and nephrotoxicity
Hemorrhagic cystitis is a dose-dependent complication of cyclophosphamide. Cystitis occurs in approximately 10% of patients, above all after high doses or longer therapy. Maintenance of fluid balance, concomitant Mesna (Uromitexan®) or fractionated doses can markedly reduce the frequency and severity of hemorrhagic cystitis.

Myelosuppression
Different degrees of myelosuppression may occur and is dose-dependent. The lowest WBC counts are most frequently seen 9 to 12 days after therapy.

Gastrointestinal

Mild or moderate nausea, vomiting and diarrhoea may occur, properly anti-emetic therapy may alleviate much of this symptoms.

Skin
Erythematous rashes and urticaria is reported in >1 of 100 patients. Premedication with corticosteroids reduces this risk.

Mesna
Mesna (Uromitexan®) is fairly well tolerated. Sometimes pain at the injection side is experienced. This side effect can be controlled by further dilution of the drug solution.

10.1.3 5-fluorouracil

Gastrointestinal
Mucositis, diarrhoea, nausea, and vomiting are reported. Stomatitis is often the first sign of toxicity.

Haematological:
Haematological toxicity is dose limiting. Leukopenia is seen mostly 9 to 14 days after treatment, but can be delayed up till 25 days. Platelets count is lowest 7 till 17 days after therapy.

Neurological toxicity
Ataxia is infrequently reported, this is more frequently seen after higher doses or dose-intense regiments.

10.1.4 Docetaxel

Detailed information on the adverse events during phase I/II trials with Taxotere is provided in the Taxotere® Product Resumé. The most commonly reported toxicities at treatment 100mg/m^2 every third week is:

Haematological:
Neutropenia is the most commonly reported side effect, (97%, severe in 76%). Anaemia (<110g/L) was commonly seen, 90% out off which 8.9% was severe.

Fever and Infection:
Febrile neutropenia is reported in 20% and infections in 12% out off which 5.7% was severe. The most frequently reported infectious complications included urinary tract infections, upper respiratory tract infections and sepsis.

Hypersensitivity reactions
The most common symptoms are erythematous rashes, urticaria, back pain and dyspnoea. Severe symptoms as dyspnoea with bronchospasm or angio oedema are rare and reversible after immediately stop of infusion and adequate therapy. Cutaneous side-effects, in most cases as erythemas at hands, feet, face and bowel, together with rash is seen in most cases within 1 week after delivery of docetaxel, and reversible after 21 days.

Fluid retention
The incidence and grade of fluid retention is cumulative. Delivery of high doses of corticosteroids together with administration of docetaxel delay the onset and reduces the magnitude of these

symptoms. Fluid retention is correlated with peripheral oedema, and in fewer cases with pleural/pericardial effusions. Fluid retention is reported to disappear after stop of docetaxel administration (median time 16.4 weeks, range 0-42).

Gastrointestinal
Nausea (41%, severe 4%), vomiting (25%, severe 3%), diarrhoea (41%, severe 4%) and mucositis (42%, severe 5.3%) have been reported. In patients with normal base-line liver function increase in liver enzymes were reported in approximately 10-20% of the patients.

Musculoskeletal
Myalgia (20%, severe in 1.4%) and arthralgia (8.6%).

Nail disorders
Nail disorders, onycholysis is reported in 28%, severe in 2.6%.

Neurological toxicity
Asthenia is reported in 63%, (severe in 11%). Neurosensoric symptoms including paresthesia, dysesthesia and pain (50%, severe in 4.1%). Neuromotor symptoms including weakness (14%, severe in 4%).

Alopecia
Alopecia is frequently reported in patients treated with docetaxel given 75 mg/m^2.

10.1.5 Filgrastim (Neupogen®), pegfilgrastim (Neulasta®)

The most commonly side effect attributed to G-CSF products is musculoskeletal pain, usually controlled by mild analgesics.

11 EVALUATION AND MONITORING

11.1 Case report forms

Clinical Research Unit, Karolinska University hospital Stockholm, will supply the Case Report Forms. The base-line inclusion form should be filled out before registration and sent to the national randomization office, by fax or web-based at the day of registration. CRF pages concerning during chemotherapy, end of treatment and follow up will be transferred to the Central Data Centre at the Clinical Research Unit, Karolinska University hospital, Stockholm.

11.2 Assessments before treatment

Investigations are done according to flow sheet in appendix 1. Staging investigations, which should be performed before inclusion, include chest x-ray, and liver enzymes, alkaline phosphatase and total bilirubin. If the patient reports bone pain or if serum alkaline phosphatase is elevated, a bone scintigram should be performed, including x-ray/MRI of eventual hot spots. Ultrasound or CT of liver is necessary only in case of abnormal transaminases and/or bilirubin or if clinical symptoms indicating presence of metastases. These investigations are considered to be normal clinical routine. Baseline X-Ray must be done 6 weeks before randomisation and ECG, MUGA-scintigram or

ECHO-cardiogram should be done within 4 weeks before registration. Blood-samples must be done within two weeks before registration. For women with child bearing potential, pregnancy test should be done within one week before registration.

11.3 Assessments during treatment

Haematological laboratory values, transaminases, alkaline phosphatase and bilirubin and non-haematological side effects are collected from every patient before each course of chemotherapy is given. It will be recorded according to the NCI CTC version 3 for side effects during each course of treatment. Haematological nadir values (leukocytes, neutrophils, platelets) will also be recorded during each cycle, for all patients.

11.4 Toxicity evaluation

Toxicity will be assessed through clinical Adverse Events and Common Toxicity Criteria (CTC) laboratory and non-laboratory toxicities. NCI CTC version 3 (appendix 2) will be used. Prior to enrolment, a baseline status must be documented to note the occurrence and nature of each patients medical condition. A CTC toxicity rating will be performed before each cycle for any adverse events that were experienced during the previous cycle.

11.5 Follow up

Follow-up data are provided by the clinician with a simple form recording the disease-status of the patient yearly up to ten years after treatment.
After completion of chemo- and radiotherapy, the patients shall be clinically examined every three to four months for 2 years, thereafter every six month up to five years, followed by yearly examinations. This consists of clinical examination (general appearance, lymph nodes, skin, breast and scar areas, heart- and lung auscultation, abdomen) and routine blood samples (haemoglobin, total white blood cells, differential count, platelets, transaminases, alkaline phosphatase, kreatinine, calcium and albumin) (No blood samples will be taken in Germany). ECHO cardiography or MUGA scan should be performed and repeated in the follow up according to national guidelines – SPC-text or as described in the previous version of this study protocol, 3 and 5 years after completion of trastuzumab therapy.

X-ray (conventional, CT, MRI, PET etc) investigations or biopsies should be carried out when clinical signs and symptoms indicate the need. Biopsy verification of relapses is strongly recommended.

11.6. Safety

The investigator is responsible for the monitoring of the safety of patients who have entered the trial and for appropriate medical care during study participation. The investigator will follow up all adverse events, regardless of severity, until satisfactory resolution.

11.7. Safety measurements

- Safety will be assessed through clinical Adverse Events and Common Toxicity Criteria (CTC) laboratory and non-laboratory toxicities. NCI CTC version 3 will be used.

- Prior to enrolment, a baseline status must be documented to note the occurrence and nature of each patients medical conditions
- A CTC toxicity rating will be performed before each cycle for any adverse events that were experienced during the previous cycle

11.8. Adverse event reporting

Patients will be instructed by the investigator to report the occurrence of any adverse event.

An **adverse event** (AE) is any undesirable reaction associated with the use of a drug, whether or not considered drug related, and includes any side effect, injury, toxicity, or sensitivity reactions. It also includes any undesirable clinical or laboratory change which does not commonly occur in the patients treated with the described drugs in this study. An adverse event that is judged to be related to study treatment is called an **adverse reaction**. An adverse reaction, the nature, or severity of which is not consistent with the applicable product information (e.g. investigator´s brochure for an unapproved investigational medicinal product or summary of product characteristics for an authorized product) is called unexpected. A pre-existing disease that worsens during the study must be notified as an AE. Symptoms of the targeted cancer (if applicable) should not be reported as adverse events.

A **serious adverse event** (SAE) is any reaction that is fatal, life-threatening, requires or prolongs hospitalization, results in persistent or significant disability or incapacity, a congenital anomaly or birth defect, an important medical event. Important medical events are those which may not be immediately life-threatening, but are clearly of major clinical significance. They may jeopardize the subject, and may require intervention to prevent one of the other serious outcomes. Cancer and drug overdose or abuse will normally be considered as serious.

Expected serious adverse reactions are listed in the Summary of Product Characteristics (SmPC). All serious unexpected adverse events judged by either the investigator or the sponsor as having a reasonable suspected causal relationship to an investigational or an accompanying medicinal product qualify as **suspected unexpected serious adverse reactions** (SUSARs).

All adverse events occurring after enrolment must be documented in the clinical Case Report Form (CRF).
Expedited reporting of Unexpected Serious Adverse Drug Reactions
Study site personnel must report an unexpected adverse drug reaction occurring during the study that results in any of the following outcomes:

- Death
- Hospitalization
- A life threatening experience
- Severe or permanent disability
- Congenital anomaly
- Medically important event

All unexpected serious adverse drug reactions have to be reported within 24 hours after notification by the study personnel using the country-specific Serious Adverse Event form to the trial offices in respective country:

- Swedish Trial Sites: SAEs are reported to the Central Data Centre/Clinical Trial Unit.
- Austrian Trial Sites: SAEs are initially reported to ABCSG Trial Office.

- Germany: SAEs are initially reported to GBG Forschungs GmbH.
- Finland: SAEs are initially reported to Central Data Centre/Clinical Trial Unit, Karolinska University Hospital, Stockholm, Sweden.

Each country centre has at the same time to send copies of each report to the Central Data Centre/Clinical Trials Unit, Karolinska University Hospital, Stockholm, Sweden. In case an SAE is regarded as a SUSAR it will be reported by the Sponsor to the Swedish Medical Product agency for Swedish patients, in Austria according to Austrian regulations for Austrian patients, in Germany according to German regulations for German patients and in Finland according to Finnish regulation for Finnish patients.
Events not considered to be serious adverse events are a progression of the breast cancer, grade 3 and 4 uncomplicated leucopenia / neutropenia and / or hospitalisations occurring under the following circumstances:
• being part of the normal treatment or monitoring of the studied treatment;
• elective surgery;

Taxotere related SUSAR events will be reported to Sanofi-Aventis, Paris, by the sponsor.

11.9 Monitoring

The study will be monitored according to GCP by regular site visits and calls. During site visits, the monitor should review original patient records and document retention. Additionally, the monitor should observe study procedure and will discuss any problem with the investigator. The investigator will provide direct access to source data/documents for trial related monitoring audits, EC review and regulatory inspections. Monitoring will be provided by the Clinical Research Unit, Karolinska University Hospital, Stockholm for Sweden , by the ABCSG Trial Office, Vienna, for Austria, by the GBG Forschungs GmbH, Neu-Isenburg, for Germany and by the FBCG for Finland.

12. COLLECTION OF DATA AND CONFIDENTIALITY

12.1. Collection of data

In order to allow the sequential statistical analysis, the Case report Forms (CRFs) should be sent, on continuous basis to the Clinical Trial Unit, as specified in the CRF. Data from the CRFs should be transferred to the Central Data Centre, at least four times per year or according to local standard.

12.2. Confidentiality of trial documents and patients records

The investigator must assure that patients' anonymity is maintained. This of course also mandatory for the studies of prognostic- and therapy predictive factors as well as toxicity studies in relation to markers present in the tumour cells or normal cells. On case report forms patients should be identified only by their initials, and by the unique randomization number.

13 ETHICS

The local ethical committee before start of the trial should approve the study protocol. The study should be performed according to the Helsinki declaration (Appendix 5), GCP and all appropriate local laws and regulations. All included patients should be informed on the design and objectives of the study and patient informed consent according to the local requirements obtained before start of treatment. The personal integrity of patients included in this study will be secured by recording only patient initials, date of birth and an unique randomization number as identification for each patient.

14 PUBLICATION POLICY

14.1. Publication

The Vancouver declaration (Br Med J: 296, 401-405, 1988) should be followed in all publication based on this trial. However, patient recruitment and management are also important facts for co-authorship. The results of the study must be published in an international scientific journal according to the publication rules of SBG together with the rules for ABCSG, GBG and FBCG.

15. INDEPENDENT DATA SAFETY AND MONITORING COMMITTEE

A committee consisting of Professor Erik Wist, Ullevåls University Hospital, Oslo, Norway, professor Jan G M Klijn, Erasmus Medical Centre,-Erasmus University, Rotterdam, The Netherlands, professor Anthony Howell, Christie Hospital NHS Foundation Trust, Manchester , Great Britain and professor Peter Bauer, Institut für Medizinische Statistik, Wien, Austria, has been established. They should receive regular updates of the inclusion status and safety status by the Central Data Centre.

16. REFERENCES

1. EBCTCG. Polychemotherapy for early breast cancer: an overview of the randomised trials. Lancet 1998;352:930-942.
2. EBCTCG. Tamoxifen for early breast cancer: an overview of the randomised trials. Lancet 1998;351:1451-1467.
3. EBCTCG. Favourable and unfavourable effects on long-term survival of radiotherapy for early breast cancer: an overview of the randomised trials. Lancet 2000;355:1757-70.
4. Clarke M, Collins R, Darby S, Davies C, Elphinstone P, Evans E, et al. Effects of radiotherapy and of differences in the extent of surgery for early breast cancer on local recurrence and 15-year survival: an overview of the randomised trials. Lancet 2005;366(9503):2087-106.
5. Henderson IC, Berry DA, Demetri GD, Cirrincione CT, Goldstein LJ, Martino S, et al. Improved outcomes from adding sequential Paclitaxel but not from escalating Doxorubicin dose in an adjuvant chemotherapy regimen for patients with node-positive primary breast cancer. J Clin Oncol 2003;21(6):976-83.
6. Citron ML, Berry DA, Cirrincione C, Hudis C, Winer EP, Gradishar WJ, et al. Randomized trial of dose-dense versus conventionally scheduled and sequential versus concurrent combination chemotherapy as postoperative adjuvant treatment of node-positive primary breast cancer: first report of Intergroup Trial C9741/Cancer and Leukemia Group B Trial 9741. J Clin Oncol 2003;21(8):1431-9.
7. Martin M, Pienkowski T, Mackey J, Pawlicki M, Guastalla JP, Weaver C, et al. Adjuvant docetaxel for node-positive breast cancer. N Engl J Med 2005;352(22):2302-13.
8. Roché H, Fumoleau P, Spielmann M, Canon J, Delozier T, Kerbrat P, et al. Five years analysis of the PACS 01 trial: 6 cycles of FEC100 vs 3 cycles of FEC100 followed by 3 cycles of docetaxel (D) for the adjuvant treatment of node positive breast cancer. In: 27th Annual San Antonio Breast Cancer Symposium; 2004; San Antonio: Breast Cancer Research and Treatment; 2004. p. Abstract 27.
9. Bergh J, Wiklund T, Erikstein B, Lidbrink E, Lindman H, Malmstrom P, et al. Tailored fluorouracil, epirubicin, and cyclophosphamide compared with marrow-supported high-dose chemotherapy as adjuvant treatment for high- risk

breast cancer: a randomised trial. Scandinavian Breast Group 9401 study [In Process Citation]. Lancet 2000;356(9239):1384-91.
10. Fossati R, Confalonieri C, Torri V, Ghislandi E, Penna A, Pistotti V, et al. Cytotoxic and hormonal treatment for metastatic breast cancer: a systemic review of published randomized trial involving 31,510 women. J Clin Oncol 1998;16:3439-3460.
11. Bonadonna G, Valagussa P. Dose-response effect of adjuvant chemotherapy in breast cancer. N Engl J Med 1981;304:10-15.
12. Wood W, Budman D, Korzun A, Cooper M, Younger J, Hart R, et al. Dose and dose intensity of adjuvant chemotherapy for stage II, node-positive breast carcinoma [published erratum appears in N Engl J Med 1994;331:139]. N Engl J Med 1994;330:1253-1259.
13. Budman D, Berry D, Cirrincione C, Henderson I, Wood W, Weiss R, et al. Dose and dose intensity as determinants of outcome in the adjuvant treatment of breast cancer. The Cancer and Leukemia Group B. J Natl Cancer Inst 1998;90:1205-1211.
14. Colleoni M, Pricer K, Catiglione-Gertsch M, Goldhirsch A, Coates A, Lindtner J, et al. Dose-response effect of adjuvant cyclophosphamide, methotrexate, 5-fluorouracil (CMF) in node-positive breast cancer. Eur J Cancer 1998;34:1693-1700.
15. Gurney H. Dose calculation of anticancer drugs: A review of the current practice and introduction of an alternative. J Clin Oncol 1996;14:2590-2611.
16. Gurney H, Ackland S, Gebski V, Farrell G. Factors affecting epirubicin pharmacokinetics and toxicity: evidence against using body-surface area for dose calculation. J Clin Oncol 1998;16:2299-2304.
17. Sandström M, Freijs A, Larsson R, Nygren P, Fjällskog M-L, Bergh J, et al. Lack of relationship between systemic exposure for the component drugs of the fluorouracil, epirubicin, and 4-hydroxycyclophosphamide regimen in breast cancer patients. J Clin Oncol 1996;14:1581-1588.
18. Saarto T, Blomqvist C, Rissanen P, Auvinen A, Elomaa I. Haematological toxicity: a marker of adjuvant chemotherapy efficacy in stage II and III breast cancer. Br J Cancer 1997;75:301-305.
19. Poikonen P, Saarto T, Lundin J, Joensuu H, Blomqvist C. Leucocyte nadir as a marker for chemotherapy efficacy in node-positive breast cancer treated with adjuvant CMF [see comments]. Br J Cancer 1999;80(11):1763-6.
20. Cameron DA, Massie C, Kerr G, Leonard RC. Moderate neutropenia with adjuvant CMF confers improved survival in early breast cancer. Br J Cancer 2003;89(10):1837-42.
21. Paridaens R, Wildiers J, Dumez H, Thomas J, Weltens C, Van den Bogaert W, et al. Impact of dose-intensity of adjuvant CMF on disease-free (DFS) and overall survival (OS) in breast cancer (BC): A retrospective analysis. In: Rowett L, editor. 27th ESMO Congress; 2002 18-22 October; Nice: Annals of Oncology, Oxford university press; 2002. p. 45, abstract 161.
22. Szutowicz E, Radecka B, Dziadziuszko R, Szwiec M, Jassem J. Peripheral blood count nadirs during adjuvant CMF chemotherapy for breast cancer - lack of prognostic relevance. In: 3rd European Breast Cancer Conference; 2002 19-23 March; Barcelona: European Journal of Cancer; 2002. p. S93, abstract 205.
23. Whitehead J. The design and analysis of sequential clinical trails. 2-nd edition ed. New York, London, Toronto, Sydney, Tokyo, Singapore: Ellis Horwood; 1992.
24. Praga C, Bergh J, Bliss J, Bonneterre J, Cesana B, Coombes RC, et al. Risk of acute myeloid leukemia and myelodysplastic syndrome in trials of adjuvant epirubicin for early breast cancer: correlation with doses of epirubicin and cyclophosphamide. J Clin Oncol 2005;23(18):4179-91.
25. Levine MN, Pritchard KI, Bramwell VH, Shepherd LE, Tu D, Paul N. Randomized trial comparing cyclophosphamide, epirubicin, and fluorouracil with cyclophosphamide, methotrexate, and fluorouracil in premenopausal women with node-positive breast cancer: update of National Cancer Institute of Canada Clinical Trials Group Trial MA5. J Clin Oncol 2005;23(22):5166-70.
26. Wilking N, Lidbrink E, Wiklund T, Erikstein B, Lindman H, Malmstrom P, et al. Long-term follow-up of the SBG 9401 study comparing tailored FEC-based therapy versus marrow-supported high-dose therapy. Ann Oncol 2007;18(4):694-700.
27. French Epirubicin Study Group. Benefit of a high-dose epirubicin regimen in adjuvant chemotherapy for node-positive breast cancer patients with poor prognostic factors: 5- year follow-up results of French Adjuvant Study Group 05 randomized trial. J Clin Oncol 2001;19(3):602-11.
28. Dang CT, D'Andrea GM, Moynahan ME, Dickler MN, Seidman AD, Fornier M, et al. Phase II study of feasibility of dose-dense FEC followed by alternating weekly taxanes in high-risk, four or more node-positive breast cancer. Clin Cancer Res 2004;10(17):5754-61.
29. Nieto Y. The verdict is not in yet. Analysis of the randomized trials of high-dose chemotherapy for breast cancer. Haematologica 2003;88(2):201-11.
30. Nitz UA, Mohrmann S, Fischer J, Lindemann W, Berdel WE, Jackisch C, et al. Comparison of rapidly cycled tandem high-dose chemotherapy plus peripheral-blood stem-cell support versus dose-dense conventional chemotherapy for adjuvant treatment of high-risk breast cancer: results of a multicentre phase III trial. Lancet 2005;366(9501):1935-44.

31. Rodenhuis S, Bontenbal M, Beex LV, Wagstaff J, Richel DJ, Nooij MA, et al. High-dose chemotherapy with hematopoietic stem-cell rescue for high-risk breast cancer. N Engl J Med 2003;349(1):7-16.
32. Peters WP, Rosner GL, Vredenburgh JJ, Shpall EJ, Crump M, Richardson PG, et al. Prospective, randomized comparison of high-dose chemotherapy with stem-cell support versus intermediate-dose chemotherapy after surgery and adjuvant chemotherapy in women with high-risk primary breast cancer: a report of CALGB 9082, SWOG 9114, and NCIC MA-13. J Clin Oncol 2005;23(10):2191-200.
33. Tallman MS, Gray R, Robert NJ, LeMaistre CF, Osborne CK, Vaughan WP, et al. Conventional adjuvant chemotherapy with or without high-dose chemotherapy and autologous stem-cell transplantation in high-risk breast cancer. N Engl J Med 2003;349(1):17-26.
34. Peters W, Ross M, Vredenburgh J, Meisenberg B, Marks L, Winer E, et al. High-dose chemotherapy and autologous bone marrow support as consolidation after standard-dose adjuvant therapy for high-risk primary breast cancer. J Clin Oncol 1993;11:1132-1143.
35. Piccart MJ, Di Leo A, Beauduin M, Vindevoghel A, Michel J, Focan C, et al. Phase III trial comparing two dose levels of epirubicin combined with cyclophosphamide with cyclophosphamide, methotrexate, and fluorouracil in node-positive breast cancer. J Clin Oncol 2001;19(12):3103-10.
36. Fisher B, Brown A, Mamounas E, Wieand S, Robidoux A, Margolese R, et al. Effect of preoperative chemotherapy on local-regional disease in women with operable breast cancer: findings from NSABP B-18. J Clin Oncol 1997;15:2483-2493.
37. Fisher B. National Surgical Adjuvant Breast and Bowel Project breast cancer prevention trial: a reflective commentary. J Clin Oncol 1999;17(5):1632-9.
38. Chan S, Friedrichs K, Noel D, Pinter T, Van Belle S, Vorobiof D, et al. Prospective randomized trial of docetaxel versus doxorubicin in patients with metastatic breast cancer. The 303 Study Group. J Clin Oncol 1999;17(8):2341-54.
39. Ravdin P, Valero V. Review of docetaxel (taxotere), a highly active new agent for the treatment of metastatic breast cancer. Semin Oncol 1995;22:17-21.
40. Nabholtz JM, Senn HJ, Bezwoda WR, Melnychuk D, Deschenes L, Douma J, et al. Prospective randomized trial of docetaxel versus mitomycin plus vinblastine in patients with metastatic breast cancer progressing despite previous anthracycline-containing chemotherapy. 304 Study Group. J Clin Oncol 1999;17(5):1413-24.
41. Sjostrom J, Blomqvist C, Mouridsen H, Pluzanska A, Ottosson-Lonn S, Bengtsson NO, et al. Docetaxel compared with sequential methotrexate and 5-fluorouracil in patients with advanced breast cancer after anthracycline failure: a randomised phase III study with crossover on progression by the Scandinavian Breast Group. Eur J Cancer 1999;35(8):1194-201.
42. Biganzoli L, Cufer T, Bruning P, Coleman R, Duchateau L, Calvert AH, et al. Doxorubicin and paclitaxel versus doxorubicin and cyclophosphamide as first-line chemotherapy in metastatic breast cancer: The European Organization for Research and Treatment of Cancer 10961 Multicenter Phase III Trial. J Clin Oncol 2002;20(14):3114-21.
43. Cortes JE, Pazdur R. Docetaxel. J Clin Oncol 1995;13(10):2643-55.
44. Jones SE, Erban J, Overmoyer B, Budd GT, Hutchins L, Lower E, et al. Randomized phase III study of docetaxel compared with paclitaxel in metastatic breast cancer. J Clin Oncol 2005;23(24):5542-51.
45. Sparano J, Wang M, Martino S, Jones V, Perez E, Saphner T, et al. Phase III study of doxorubicin-cyclophosphamide followed by paclitaxel or docetaxel given every 3 weeks or weekly in patients with axillary node-positive or high-risk node-negative breast cancer: results of North American Breast Cancer Intergroup Trial E1199. In: 28th Annual San Antonio Breast Cancer Symposium; 2005; San Antonio, TX: Breast Cancer Research and Treatment; 2005. p. S5.
46. Roche H, Fumoleau P, Spielmann M, Canon JL, Delozier T, Serin D, et al. Sequential Adjuvant Epirubicin-Based and Docetaxel Chemotherapy for Node-Positive Breast Cancer Patients: The FNCLCC PACS 01 Trial. J Clin Oncol 2006.
47. Crown J, Francis P, Di Leo A, Buyse M, Balil A, Anderson A, et al. Docetaxel (T) given concurrently with or sequentially to anthracycline-based (A) adjuvant therapy (adjRx) for patients (pts) with node-positive (N+) breast cancer (BrCa), in comparison with non-T adjRx: First results of the BIG 2-98 Trial at 5 years median follow-up (MFU). In: ASCO Annual Meeting; 2006; Atlanta, GA: Journal of Clinical Oncology, 2006 ASCO Annual Meeting Proceedings Part I; 2006. p. June 20 Supplement, abstract LBA519.
48. Fountzilas G, Athanassiades A, Giannakakis T, Bafaloukos D, Karakousis K, Dombros N, et al. A phase II study of paclitaxel in advanced breast cancer resistant to anthracyclines. Eur J Cancer 1996;32A(1):47-51.
49. Gianni L, Munzone E, Capri G, Fulfaro F, Tarenzi E, Villani F, et al. Paclitaxel by 3-hour infusion in combination with bolus doxorubicin in women with untreated metastatic breast cancer: high antitumor efficacy and cardiac effects in a dose-finding and sequence-finding study. J Clin Oncol 1995;13(11):2688-99.
50. Seidman AD, Tiersten A, Hudis C, Gollub M, Barrett S, Yao TJ, et al. Phase II trial of paclitaxel by 3-hour infusion as initial and salvage chemotherapy for metastatic breast cancer. J Clin Oncol 1995;13(10):2575-81.

51. Mamounas EP, Bryant J, Lembersky B, Fehrenbacher L, Sedlacek SM, Fisher B, et al. Paclitaxel after doxorubicin plus cyclophosphamide as adjuvant chemotherapy for node-positive breast cancer: results from NSABP B-28. J Clin Oncol 2005;23(16):3686-96.
52. Citron M, Berry D, Cirrincione C, Carpenter J, Hudis C, Gradishar W, et al. Superiority of dose-dense (DD) over conventional scheduling (CS) and equivalence of sequential (SC) vs combination adjuvant chemotherapy (CC) for node-positive breast cancer. In: Lippman ME, editor. 25th Annual San Antonio Breast Cancer Symposium; 2002 December 11-14; San Antonio, USA: Kluwer Academic Publishers; 2002. p. 32, abstract 15.
53. Moebus V, Lueck H, Thomssen C, Kuhn W, Kurbacher C, Nitz U, et al. Dose-dense sequential chemotherapy with epirubicin (E), paclitaxel (T) and cyclophosphamide (C) (ETC) in comparison to conventional dosed chemotherapy in high-risk breast cancer patients (4+ LN). Mature results of an AGO-trial. In: 29th Annual San Antonio Breast Cancer Symposium; 2006; San Antonio, Tx: Breast Cancer Research and Treatment; 2006. p. S20, abstract 43.
54. Möbus V, Untch M, Du Bois A, Lueck H-J, Thomssen C, Kuhn W, et al. Dose-dense sequential chemotherapy with epirubicin(E), paclitaxel (T) and cyclophosphamide (C) (ETC) is superior to conventional dosed chemotherapy in high-risk breast cancer patients (= 4 +LN). First results of an AGO-trial. In: ASCO Annual Meeting; 2004; New Orleans; 2004.
55. Green MD, Koelbl H, Baselga J, Galid A, Guillem V, Gascon P, et al. A randomized double-blind multicenter phase III study of fixed-dose single-administration pegfilgrastim versus daily filgrastim in patients receiving myelosuppressive chemotherapy. Ann Oncol 2003;14(1):29-35.
56. Holmes FA, Jones SE, O'Shaughnessy J, Vukelja S, George T, Savin M, et al. Comparable efficacy and safety profiles of once-per-cycle pegfilgrastim and daily injection filgrastim in chemotherapy-induced neutropenia: a multicenter dose-finding study in women with breast cancer. Ann Oncol 2002;13(6):903-9.
57. Holmes FA, O'Shaughnessy JA, Vukelja S, Jones SE, Shogan J, Savin M, et al. Blinded, randomized, multicenter study to evaluate single administration pegfilgrastim once per cycle versus daily filgrastim as an adjunct to chemotherapy in patients with high-risk stage II or stage III/IV breast cancer. J Clin Oncol 2002;20(3):727-31.
58. Johnston E, Crawford J, Blackwell S, Bjurstrom T, Lockbaum P, Roskos L, et al. Randomized, dose-escalation study of SD/01 compared with daily filgrastim in patients receiving chemotherapy. J Clin Oncol 2000;18(13):2522-8.
59. von Minckwitz G, Blohmer J, Löhr A, Raab G, Eidtmann H, Gerber B, et al. Primary prophylaxis with 3 weekly pegfilgrastim and ciprofloxacin effectively prevent (febrile) neutropenia and infection during neoadjuvant chemotherapy with docetaxel/doxirubicin/cyclophosphamide (TAC) in breast cancer patients. In: ASCO Annual Meeting; 2005; 2005. p. abstract 8008.
60. Piantodosi S. Clinical trials, a methodological perspective. New York: Wiley; 1997.
61. Dupont WD, Plummer WD, Jr. Power and sample size calculations. A review and computer program. Control Clin Trials 1990;11(2):116-28.
62. Romond EH, Perez EA, Bryant J, Suman VJ, Geyer CE, Jr., Davidson NE, et al. Trastuzumab plus adjuvant chemotherapy for operable HER2-positive breast cancer. N Engl J Med 2005;353(16):1673-84.
63. Smith I, Procter M, Gelber RD, Guillaume S, Feyereislova A, Dowsett M, et al. 2-year follow-up of trastuzumab after adjuvant chemotherapy in HER2-positive breast cancer: a randomised controlled trial. Lancet 2007;369(9555):29-36.
64. Suter TM, Procter M, van Veldhuisen DJ, Muscholl M, Bergh J, Carlomagno C, et al. Trastuzumab-associated cardiac adverse effects in the herceptin adjuvant trial. J Clin Oncol 2007;25(25):3859-65.
65. Tan-Chiu E, Yothers G, Romond E, Geyer CE, Jr., Ewer M, Keefe D, et al. Assessment of cardiac dysfunction in a randomized trial comparing doxorubicin and cyclophosphamide followed by paclitaxel, with or without trastuzumab as adjuvant therapy in node-positive, human epidermal growth factor receptor 2-overexpressing breast cancer: NSABP B-31. J Clin Oncol 2005;23(31):7811-9.

17. APPENDIX

Appendix 1. Investigations

Appendix 2. NCI common toxicity criteria VERSION 3

Appendix 3. New York Heart Association classification of congestive heart failure

Appendix 4. Declaration of Helsinki

Appendix 5 ASSESSMENT OF HEALTH RELATED QUALITY OF LIFE

Appendix 1. Investigations

**Parameter**	**Prestudy[1]**	**Day 8, 11/12 and 14/15**	**Day 1 all courses**	**Off treatment**	**Follow Up[7]**
History	X				
Physical examination	X		X[10]	X	X[7]
Pregnancy test[2]	X				
Body surface area	X		X		
Body weight	X		X		
Hemoglobine	X	X	X[10]	X	X[7]
Leukocytes including neutrophils	X	X	X[10]	X	X[7]
Platelets	X	X	X[10]	X	X[7]
Alkaline Phosphatase	X		X[10]	X	X[7]
ASAT, ALAT	X		X[10]	X	X[7]
S-bilirubin	X		X[10]	X	X[7]
Creatinine	X			X	X[7]
Calcium	X			X	X[7]
Albumin	X			X	X[7]
Performance status	X				
ECG	X				
Chest X-ray or chest-CT	X				
Bone scintigraphy/ X-ray/MRI	X[3]				X[7]
Liver CT or US	X[4]				X[4]
Receptor status	X				
Biological markers	X				
Toxicity assessment[6]	X		X	X	X[5]
MUGA	X[8]				
ECHO	X[8]				
**Quality of Life[9]**	**X[9]**		**X[9]**		**X[9]**

1.Informed consents prior to prestudy investigations. Radiological procedures (X-ray) within six weeks and blood tests within two weeks prior randomisation.
2. Women of child bearing potential must have a negative pregnancy test performed within 1 week prior randomisation. In Austria, further pregnancy tests have to be performed monthly during the treatment phase.
3. Necessary only if serum alkaline phosphatase is elevated or if the patients have symptoms indicating skeletal metastases. All abnormal regions on scan should be verified by bone x-ray. Instead of scintigraphy x-ray of the spine and pelvis is allowed performed.

4. Necessary only if liver enzymes or serum bilirubin are abnormal or if signs suggesting metastases are present
5. Regularly until toxicities have resolved or are deemed irreversible by the investigator
6. All relevant and/or serious adverse events occurring during the chemotherapy period or within 30 days following the last infusion of chemotherapy must be reported and followed until resolved. All items in the CRF must be checked at base line and after each course. Any late SAE (occurring after this 30 day period) possibly or probably related to the study chemotherapy should follow the same reporting.
7. The patients shall be clinically examined every three to four month for 2 years, thereafter every sixth monthsin the years 3,4 and 5, followed by yearly examinations during the years 5,6,7,8, 9 and 10from randomisation. This consists of clinical examination (general appearance, lymph nodes, skin, breast and scar areas, heart- and lung auscultation, abdomen) and routine blood samples (haemoglobin, total white blood cells, differential count, platelets, transaminases, alkaline phosphatase, bilirubin, creatinine, calcium and albumin) (No blood samples to be taken in Germany).
X-ray (conventional, CT, MRI, PET etc) investigations or biopsies should be carried out when clinical signs and symptoms indicate the need. Biopsy verification of relapses is strongly recommended.
8. Either or. ECG, ECHO or MUGA within 4 weeks prior to randomization
9. Sweden and Austria: Quality of Life questionnaires EORTC QLQ-C30, QLQ-BR23 and FACT-An+F (Austria only EORTC QLQ-C30 and QLQ-BR23) should be used. Baseline assessment will be performed after informed consent has been obtained, but *before* randomisation and then at week 6 after start of treatment, week 15 after start of treatment, and at follow-up 4 months ± 1 week, 8 months ± 1 week and 12 months ±1 week. See Appendix 5 for more details.
10. It is acceptable to perform physical examination and required blood tests the day before the chemotherapy is administered.

Appendix 2. Recommendations for grading of acute and subacute toxicity

CTCAE v.3

Appendix 3. New York Heartt Association Classification of Congestive Heart Failure

Class I **No limitation of physical activity. Ordinary physical activity does not cause undue fatigue, palpitation or dyspnea.**

Class II **Slight limitation of physical activity. Comfortable at rest, but ordinary physical activity results in fatigue, palpitation or dyspnoea.**

Class III **Marked limitation of physical activity. Comfortable at rest, but less than ordinary activity causes fatigue, palpitation or dyspnoea.**

Class IV Unable to carry out any physical activity without discomfort. Symptoms of cardiac insufficiency may be present even at rest. If any physical activity is undertaken, discomfort is increased

Appendix 4. Declaration of Helsinki

World Medical Association Declaration of Helsinki
Ethical Principles for Medical Research Involving Human Subjects
Adopted by the 18th WMA General Assembly, Helsinki, Finland, June 1964
and amended by the 29th WMA General Assembly, Tokyo, Japan, October 1975
35th WMA General Assembly, Venice, Italy October 1983
41st WMA Gernal Assembly, Hong Kong, September 1989
48th WMA General Assembly, Somerset West, Republic of South Africa, October 1996 and the
42nd General Assembly, Edinburgh, Scotland, October 2000

II A. Introduction

1. The World Medical Association has developed the Declaration of Helsinki as a statement of ethical principles to provide guidance to physicians and other participants in medical research involving human subjects. Medical research involving human subjects includes research on identifiable human material or identifiable data.
2. It is the duty of the physician to promote and safeguard the health of the people. The physician´s knowledge and conscience are dedicated to the fullfilllment of this duty.
3. The Declaration of Geneva of the World Medical Association binds the physician with the words, "The health of my patient will be my first consideration" and the International Code of Medical Ethics declares that. "A Physician shall act only in the patient´s interest when providing medical care which might have the effect of weakening the physical and mental condition of the patient."
4. Medical progress is based on research which ultimately must rest in part on exprementation involving human subjects.
5. In medical research on human subjects, considerations related to the well-being of the human subject should take precedence over the interest of science and society.
6. The primary purpose of medical research involving human subjects is to improve prophylactic, diagnostic and therapeutic procedures and the understanding of the aetiology and patheogenesis of disease. Even the best proven prophylactic, diagnostic, and therapeutic methods must continuously be challenged through research for their effectiveness, efficiency, accessibility and quality.
7. In current medical practice and in medical research, most prophylactic, diagnostic and therapeutic procedures involve risks and burdens.
8. Medical research is subject to ethical standards that promote respect for all human beings and protect their health and rights. Some research populations are vulnerable and need special protection. The particular needs of the economically and medically disadvantaged must be recognized. Special attention is also required for those who cannot give or refuse consent for themselves, for those who may be subject to giving consent under duress, for those who will not benefit personally from the research and for those for whom the research is combined with care.
9. Research investigators should be aware of the ethical, legal and reulatory requirements for resarch on human subjects in their own countries as well as applicable international requirements. No national ethical, legal or regulatory requirement should be allowed to reduce or eliminate any of the protections for human subjects set fourth in this Declaration.

II B. Basic principles for all medical research

10. It is the duty of the physician in medical research to protect live, health, privacy and dignity of the human subject.
11. Medical research involving human subjects must conform to generally accepted scientific principles, be based on a thorough knowledge of the scientific literature, other relevant sources of information and on adequate laboratory and, where appropriate, animal experimentation.

12. Appropriate caution must be exercised in the conduct of research which may affect the environment, and the welfare of animals used for research must be respected.
13. The design and performance of each experimental procedure involving human subjects should be clearly formulated in an experimental protocol. This protocol should be submitted for consideration, comment, guidance, and where appropriate, approval to a specially appointed ethical review committee, which must be independent of the investigator, the sponsor or any other kind of undue influence. This independent committee should be in conformity with the laws and regulations of the country in which the research experiment is performed. The committee has the right to monitor ongoing trials. The researcher has the obligation to provide monitoring information to the committee, especially any serious adverse events. The researcher should also submit to the committee, for review, information regarding funding, sponsors, institutional affiliations other potential conflicts of interest and incentives for subjects.
14. The research protocol should always contain a statement of the ethical considerations involved and should indicate that there is compliance with the principles enunciated in this Declaration.
15. Medical research involving human subjects should be conducted only by scientifically qualified persons and under the supervision of a clinically competent medical person. The responsibility for the human subject must always rest with a medically qualified person and never rest on the subject of the research, even though the subject has given consent.
16. Every medical research project involving human subjects should be preceded by careful assessment of predictable risks and burdens in comparison with foreseeable benefits to the subject or to others. This does not preclude the participation of healthy volunteers in medical research. The design of all studies should be publicly available.
17. Physicians should abstain from engaging research projects involving human subjects unless they are confident that the risks involved have been adequately assessed and can be satisfactorily managed. Physicians should cease any investigation of the risks are found to outweigh the potential benefits or if there is conclusive proof of positive and beneficial results.
18. Medical research involving human subjects should only be conducted if the importance of the objective outweighs the inherent risks and burdens to the subject. This is especially important when the human subjects are healthy volunteers.
19. Medical research is only justified if there is a reasonable likelihood that the populations in which the research is carried out stand to benefit from the results of the research.
20. The subjects must be volunteers and informed participants in the research projct.
21. The right of research subjects to safeguard their integrity must always be respected. Every precaution should be taken to respect the privacy of the subject, the confidentiality of the patient´s information and to minimize the impact of the study on the subj4ect´s physical and mental integrity and on the personality of the subject.
22. In any research on human beings, each potential subject must be adequately informed of the aims, methods, sources of funding, any possible conflicts of interest, institutional affiliations of the researcher, the anticipated benefits and potential risks of the study and the discomfort it may entail. The subject should be informed of the right to abstain from participation in the study or to withdraw consent to participate at any time without reprisal. After ensuring that the subject has understood the information, the physician should then obtain the subject´s freely-given informed consent, preferably in writing. If the consent cannot be obtained in writing, the non-written consent must be formally documented and witnessed.
23. When obtaining informed consent for the research project the physician should be particularly cautious if the subject is in a dependent relationship with the physician or may consent under duress. In that case the informed consent should be obtained by a well-informed physician who is not engaged in the investigation and who is completely independent of this relationship.
24. For a research subject who is legally incompetent, physically or mentally incapable of giving consent or is a legally incompetent minor, the investigator must obtain informed consent from the

legally authorized representative in accordance with applicable law. These groups should not be included in research unless the research is necessary to promote the health of the population represented and this research cannot instead be performed on legally competent persons.

25. When a subject deemed legally incompetent, such as a minor child, is able to give assent to decisions about participation in research, the investigator must obtain that assent in addition to the consent of the legally authorized representative.

26. Research on individuals from whom it is not possible to obtain consent, including proxy or advance consent, should be done only if the physical/mental condition that prevents obtaining informed consent is a necessary characteristic of the research population. The specific reasons for involving research subjects with a condition that renders them unable to give informed consent should be stated in the experimental protocol for consideration and approval of the review committee. The protocol should state that consent to remain in the research should be obtained as soon as possible from the individual or a legally authorized surrogate.

27. Both authors and publishers have ethical obligations. In publication of the results of research, the investigators are obliged to preserve the accuracy of the results. Negative as well as positive results should be published or otherwise publicly available. Sources of funding, institutional affiliations and any possible conflicts of interest should be declared in the publication. Reports of experimentation not in accordance with the principles laid down in this Declaration should not be accepted for publication.

II C. Additional principles for medical research combined with medical care.

28. The physician may combine medical research with medical care, only to the extent that the research is justified by its potential prophylactic, diagnostic or therapeutic value. Then medical research is combined with medical care, additional standards apply to protect the patients who are research subjects.

29. The benefits, risks, burdens and effectiveness of a new method should be tested against those of the best current prophylactic, diagnostic, and therapeutic methods. This does not exclude the use of placebo, or no treatment, in studies where no proven prophylactic, diagnostic or therapeutic method exists.

30. At the conclusion of the study, every patient entered into the study should be assured of access to the best proven prophylactic, diagnostic and therapeutic methods identified by the study-

31. The physician should fully inform the patient which aspects of the care are related to the research. The refusal of a patient to participate in a study must never interfere with the patient-physician relationship.

32. In the treatment of a patient, where proven prophylactic, diagnostic and therapeutic methods do not exist or have been ineffective, the physician, with informed consent from the patient, must be free to use unproven or new prophylactic, diagnostic and therapeutic measures, if in the physician´s judgement it offers hope of saving life, re-establishing health or alleviating suffering. Where possible these measures should be made the object of research, designed to evaluate their safety and efficacy. In all cases, new information should be recorded and, where appropriate, published. The other relevant guidelines of this Declaration should be followed.

Appendix 5 ASSESSMENT OF HEALTH RELATED QUALITY OF LIFE

Rationale
A number of studies have shown effects on health related quality of life (HRQOL) of adjuvant chemotherapy in breast cancer patients (Brandberg et al., 2003). In addition, fatigue has been reporter as a major concern for cancer patients during treatment (Servaes et al., 2002). It is not known if the two regimens tested in the present trial result in differences in HRQOL and fatigue. Thus, HRQOL evaluation including assessment of fatigue will be included as a secondary end-point.

Design
HRQOL will be evaluated in a longitudinal design in all patients entered in the study. Baseline assessment will be performed after informed consent has been obtained, but before randomisation. This is extremely important while the information to the patient about the result of the randomisation might have impact on HRQOL. Follow-up assessments will be conducted at five points: week 6 after start of treatment (at course 4 in the tailored and dose dense arm (during the EC-part), Arm A, and at course 3 in the FEC-part of the PACS 01 arm, Arm B, week 15 after start of treatment (at course 8 in Arm A and at course 6 in Arm B, both during the docetaxel part), and at follow-up 4 months ± 1 week, 8 months ± 1 week months ± 1 week and 12 months ±1 week after end of treatment.

Questionnaires
EORTC QLQ-C30 (version 3.0) is a HRQOL instrument designed to be cancer specific, multidimensional in structure, appropriate for self-administration and applicable across various cultural settings (Aaronson et al., 1993). It was developed for use in cancer clinical trials reflecting the multidimensional concept of HRQOL. It includes nine multi-item scales and six single items. Five functioning scales involve physical (PF), role (RF), emotional (EF), social (SF) and cognitive (CF) functioning, as well as a global health (QL). Three symptom scales pertain to nausea and vomiting (NV), fatigue (FA) and pain (PA). Additional symptoms measured by single items include dyspnoea (DY), sleep disturbance (SL), appetite loss (AP), constipation (CO) and diarrhoea (DI). One single item asks about financial difficulties (FI) due to disease and/or treatment.

EORTC QLQ Breast cancer module, QLQ-BR23 (version 1.0) comprises 23 items, constituting five multi-item scales assessing disease symptoms such as arm and breast symptoms, side effects of treatment (surgery, chemotherapy, radiotherapy and hormonal treatment), body image and sexual functioning (Sprangers et al., 1996). In addition, single items assess sexual enjoyment, hair loss and future perspectives.

The Functional Assessment of Cancer Therapy-Fatigue (FACT-F) and FACT-Anemia (FACT-An) address by 20 items fatigue (13 items) and anemia related symptoms (7 items). The total scores of both scales have been shown to differentiate patients by hemoglobin level and patient rated performance status (Yellen et al., 1997).

Data collection procedure
The physician including the patient in the study introduces the HRQOL-evaluation and the patient receives the baseline questionnaire together with an information sheet and an envelope. After completion, the questionnaire in the envelope is collected by the physician who gives it to the research nurse. The reason for using an envelope at this assessment point is to prevent that the treating physician has access to the questionnaire. This enables the patient to respond more freely, minimizing bias due to social desirability. At subsequent HRQOL assessments, the patient is given

the questionnaire by the research nurse. The questionnaire should be completed before any examination or treatment in order to avoid the responses to be influenced by medical information given. The research nurse collects the questionnaire. No staff responsible for the treatment of the patient will have access to the questionnaires and it is important that the patient is informed about this procedure. The questionnaires are sent to the study coordinating centre. Analysis of questionnaire data will be performed according to the recommendations by the original authors. Differences between the randomisation arms and over time will be evaluated.

References

Aaronson NK, Ahmezai S, Bergman B, et al. The European Organization for Research and Treatment of Cancer QLQ-C30: A quality of life instrument for use in international clinical trials in oncology. J Natl Cancer Inst 1993; 85: 365-376.

Brandberg Y, Michelson H, Nilsson B et al. Quality of life in women with breast cancer during the first year after random assignment to adjuvant treatment with marrow-supported high-dose chemotherapy with cyclophosphamide thiotepa, and carboplatin or tailored therapy with flourouracil, epirubicin, and cyclophophamide: Scandinavian Breast Group Study 9401. JCO 2003; 21: 3659-64.

Servaes P, Verhagen S, Bleijenberg G. Determinants of chronic fatigue in disease-free breast cancer patients: a cross-sectional study. Ann Oncol 2002; 13. 589-598.

Sprangers MAG, Groenvold M, Arraras JI, et al. The European Organization for Research and Treatment of Cancer breast cancer-specific quality-of-life questionnaire Module: first results from a three-country field study. J Clin Oncol 1996; 14: 2756-2768.

Yellen SB, Cella DF, Webster K et al. Measuring fatigue and other anemia-related symptoms with the Functional Assessment of Cancer Therapy (FACT) measurement system. J Pain Symptom Manage 1997; 13: 63-74.
